# An innovative platform for quick and flexible joining of assorted DNA fragments

**DOI:** 10.1038/srep19278

**Published:** 2016-01-13

**Authors:** Henrique Cestari De Paoli, Gerald A. Tuskan, Xiaohan Yang

**Affiliations:** 1 Oak Ridge National Laboratory, Biosciences Division, Oak Ridge, 37831, USA

## Abstract

Successful synthetic biology efforts rely on conceptual and experimental designs in combination with testing of multi-gene constructs. Despite recent progresses, several limitations still hinder the ability to flexibly assemble and collectively share different types of DNA segments. Here, we describe an advanced system for joining DNA fragments from a universal library that automatically maintains open reading frames (ORFs) and does not require linkers, adaptors, sequence homology, amplification or mutation (domestication) of fragments in order to work properly. This system, which is enhanced by a unique buffer formulation, provides unforeseen capabilities for testing, and sharing, complex multi-gene circuitry assembled from different DNA fragments.

Synthetic biology encompasses conceptual design, construction, analysis, evaluation, tuning and remodeling of genetic circuits. Genetic circuits, in this context, consist of the systematic interactions between various molecular components (e.g., DNA activation/repression, RNA secondary structure, protein-dependent signaling, (in)organic molecules gradients) that are responsible for controlling and adjusting function and behavior in an organism. These principles have been developed and deployed in several organisms^1^,^2^,^3^,^4^,^5^,^6^,^7^,^8^,^9^,^10^,^11^. Such studies and progresses however would never have been possible without the advances in the cornerstone of synthetic biology: DNA synthesis and assembly.

A plethora of cloning methods is available for handling genes and/or gene parts, gene pathways and even subgenomes. These methods are typically based on either sequence homology (e.g., isothermal assembly^12^, recombination^13^) or sequence signatures (also known as prefix and suffix) left by restriction digestion followed by ligation of DNA (e.g., BioBricks^14^, GoldenGate^15^) (for a review, see^16^). Inevitably, each method has its own disadvantages, and so far, a platform capable of uniting flexibility, fidelity, efficiency and universality for unbiased handling of multiple DNA segments has yet to be developed. The homology-based methods require sequence overlap, which limit the type and order of fragment cloning. Some strategies, as designing adaptors that allow for sequences to be part of alternate libraries, only partially surpasses this limitation and in the process create scars and intermediary products are often incompatible with future assembling units^17^. Moreover, PCR-based methods are error prone and the restriction enzyme-based methods require specific recognition sequences to be present at specific sites and will in turn limit the number of fragments based on the number of restriction sites that can be used^6^,^14^. Alternatively, *type IIS* restriction enzymes, which recognize sequences outside the cleavage sites, allow a programmable signature^15^ and two sets of such enzymes can be used in an alternating pattern, within a proprietary vector, to form a ‘cloning loop’. Such principle was recently revealed in the GoldenBraid (GB) method, which introduced the term *endless assembly*^18^,^19^. Upon creation of different gene collections, carrying an user-defined 4 nucleotides signature, the GB method provides an alternative to homology-based methods by building some transcriptional units and joining them together *in vitro*. On the other hand, all currently available *type IIS*-based cloning systems require multiple libraries, use linkers/adaptors to produce functional parts, involve software to assist the construct design^20^ and leave non-standard signatures making it difficult to stablish a common platform for different laboratories. All obstacles aforementioned would be surpassed if a pre-defined three nucleotides signature could be adopted; however, a pair of such enzymes that uniquely recognize two different restriction sites is currently not available^21^.

Still, restriction enzyme-based methods often obligate a mutation step to be performed within the fragment of interest (FOI) at the enzyme recognition sequence in order to properly manipulate the DNA segment, a process called *domestication*. The prescribed need to use overlap from homology-based methods and the domestication from restriction enzymes-based methods strongly restricts or even excludes several FOI (e.g., regulatory regions) in multigene assemblies. Therefore, to properly support synthetic biology and genetic circuit engineering, within the framework of screening and analyzing many alternative and sharable network designs experimentally, these hurdles at the cloning level must be overcome. In this context, we engineered an innovative cloning system, which adopts a pre-defined three nucleotides (TNT) signature, an optimized buffer system for quick *one-pot* (i.e., digestion and ligation) reactions, as well as a method for alleviating the domestication process, creating a clean, ultra-flexible and all-inclusive system. We demonstrate its worth by readily assembling functional constructs formed from different DNA fragments present in a single universal library to create a high-fidelity platform. The TNT-cloning system will properly support synthetic biology and genetic circuit engineering particularly facilitating the modification of plants for food and energy or microbes for chemicals, drugs and vaccines production.

## Results

### The framework of TNT-cloning system

**Figure 1:**
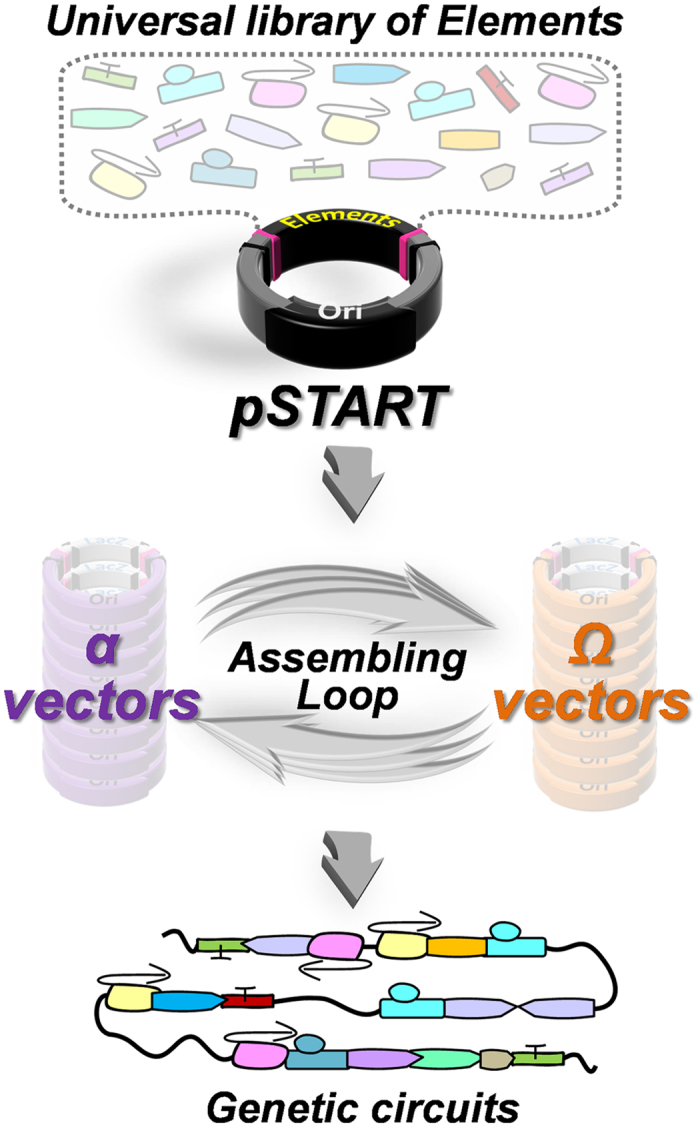
TNT-Cloning principle. One universal library in pSTART carries all DNA elements (Synthetic Biology Open Language, SBOL compliant^36^) to produce multi-gene constructs by alternating use of two separate families of vectors, *alpha (α*; purple) and *omega* (*Ω*; orange) through the assembling loop.

**Figure 2:**
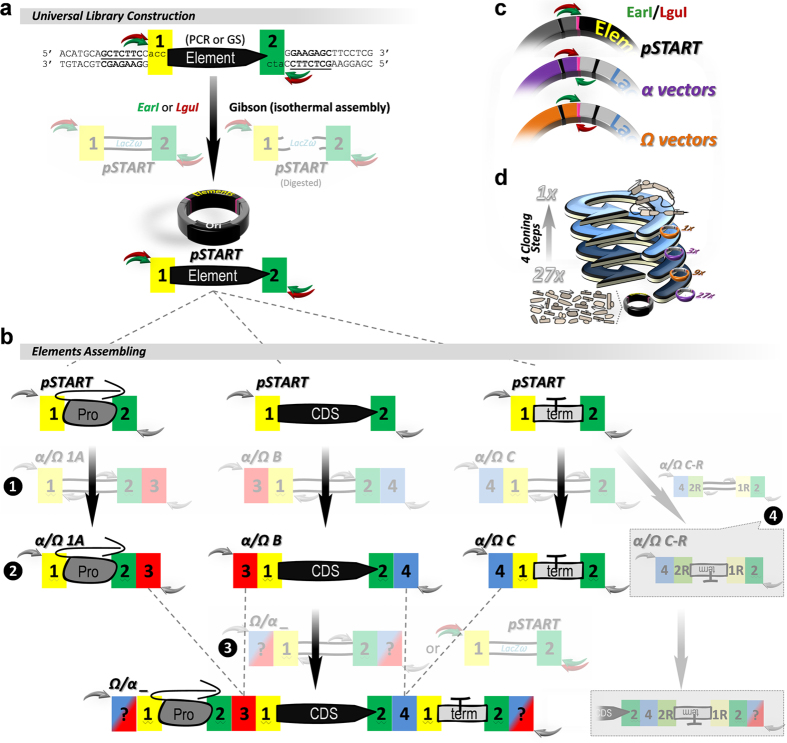
Universal library construction and assembling steps. (**a**) Fragments of interest (element) can be produced by gene synthesis (“GS”) or amplified by PCR using the sequence shown (plus the three nucleotide code for signatures 1 and 2) to be inserted in the pSTART vector by either restriction enzymes (EarI/LguI) or “Gibson” isothermal assembly (which requires previous linearization of pSTART). pSTART carries signatures 1 (yellow) and 2 (green) used to transfer the fragment from the library to any member of either *α* family (using EarI, green arrow) or *Ω* family (using LguI, red arrow), (see also **c**). (**b**) Exemplification of a three fragment assembly after cloning the elements *Pro, CDS* and *term* in the library (pSTART) as shown in **a**. Either *α* or *Ω* versions 1A, B and C are individually (➊) used as destination vectors for *Pro, CDS* and *term*, respectively, generating the constructs *Pro(α/Ω1A), CDS(α/ΩB)* and *term(α/ΩC)* (➋). Following, all three constructs are now used as entry clones and combined with a new destination vector in one single tube (➌; any Ω/any α, respectively). Signatures 3 (red) and 4 (blue) will be used to join two (only signature 3) or three (both signatures 3 and 4) fragments together. Alternatively, the destination vector can be the pSTART, in case a construct is desired as an element in the library. After two rounds of cloning, all three elements were joined without the need of adaptors/linkers or homology between sequences. Versions “R” in both families were created to allow fragment reorientation (sense or anti-sense) (e.g., ➍), no other adjustment is needed. (**c**) Detailed positioning of each enzyme in pSTART, *α* and *Ω* members. (**d**) Transfer of 27 hypothetical elements from pSTART into either *α* or *Ω* family members using the “TNT-cloning loop”. Elements are first transferred to *α* members (in this example) and combined, three at a time, to generate 9, 3 and finally 1 single insert after only 4 cloning steps (equals to 5 days if a fast growing *E. coli* strain is used, e.g. Mach1^TM^-T1^R^ or T7Express).

We conceived and developed a cloning platform that adopts a truly universal entry vector (pSTART) to carry all DNA elements to be joined by reiterative digestion/ligation steps using two families of assembling vectors, called alpha (α) and omega (Ω), which are capable of defining the order and orientation of each DNA element desired in the final construct (Fig. 1). Such element organization is determined by specific signatures (1, 2, 3, 4, 1R and 2R) left by the *type IIS* enzymes chosen, EarI and LguI, that allow, a) an ORF compatible 3 nucleotide (nt) overhang for cloning, b) up to three elements to be combined at once per round of assembly, and c) the pSTART to be used as destination vector to make new assemblies an entry element in the library, maximizing exchangeability (Fig. 2, Supplementary Fig. 1).

First, elements are either amplified or synthesized to include signatures “1” and “2” at the borders and cloned in the pSTART vector to build the universal library (Fig. 2a). The pSTART receives and releases the desired fragments with either EarI or LguI enzyme. Once elements are cloned, they are transferred and further combined in either *alpha* (*α*) or *omega* (*Ω*) vectors, which receive elements upon cleavage with EarI/LguI and release fragments upon cleavage with LguI/EarI, respectively (Fig. 2b,c). Upon digestion of each plasmid, a set of “signatures” that were specifically arranged to direct and orient the desired fragments are exposed (Fig. 2b, Supplementary Fig. 1). The signatures “1” and “2” are always flanking the inserts released from pSTART and are always used to join the final constructs into any *α* or *Ω* member. At the same time, the signatures “3” and “4” will be used by a specific member of each family (*α* and *Ω*) to join fragments between themselves, two fragments at once (binary assembly) using the members *α1A* and *α2* (or *Ω1A* and *Ω2*) and three fragments at once (tertiary assembly) using the members *α1A, αB* and *αC* (or *Ω1A, ΩB* and *ΩC*) (Fig. 2b, Supplementary Fig. 1). To change the fragment orientation (sense or anti-sense) simply switch the chosen *α* or *Ω* version for its respective “R” version during the cloning step, no adjustments are necessary (Fig. 2b, Supplementary Fig. 1). The enzyme location and the signatures were designed to permit a pre-established cloning setup and to allow each final construct to be used as an insert in case a following round of cloning is needed, creating a cloning loop that can be repeated over and over, alternating *α* and *Ω* members, in order to join multiple fragments into one larger construct. For example, 27 hypothetical fragments can be customized into one single insert at any combination through just 4 cloning rounds (Fig. 1d). The detailed representation of each vector member and the 3 nucleotides sequence of each signature followed by an ideogram (with a timeline included) are depicted on Supplementary Fig. 1 and Supplementary Fig. 2, respectively.

### Development of EarI as a useful enzyme: methylation sensitivity

**Figure 3:**
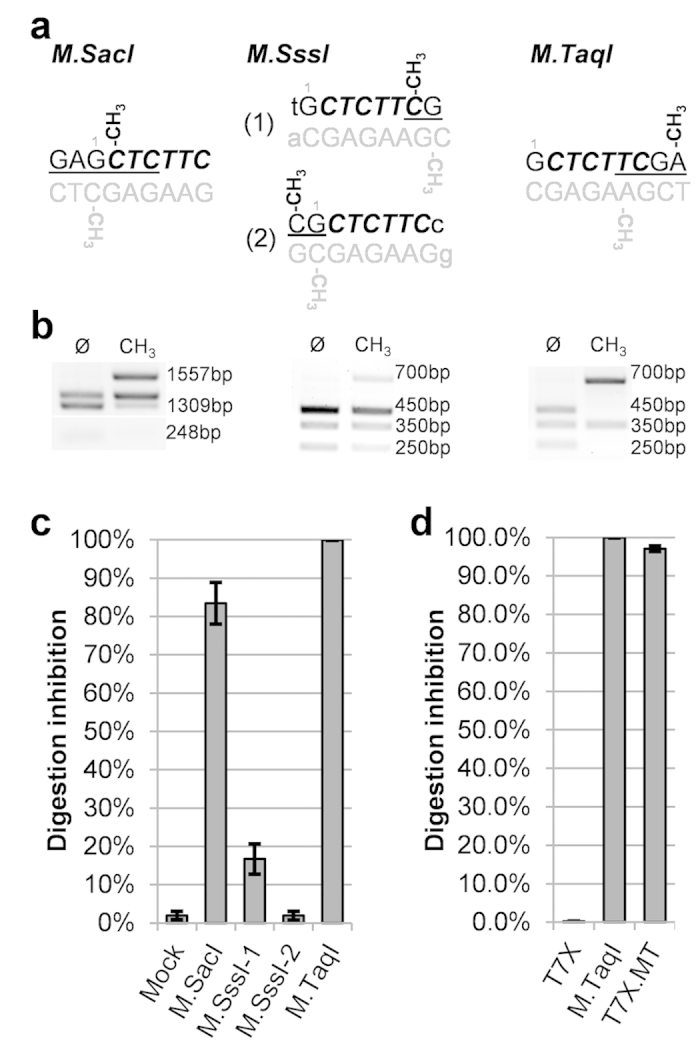
EarI sensitivity to methylation. **(a)** Methylases studied with their respective binding sites (underlined) and targeted residues (–CH_3_) on forward (black) and reverse (gray) strands. The EarI recognition site is indicated in bold/italic and position number 1 of the 5′GCTCTTC3′ site is indicated in gray numbering. **(b)** Agarose gel showing methylation-dependent inhibition of EarI activity. *Left panel:* M.SacI was expressed in *E coli* T7Express (T7X) and the expression plasmid carrying the site shown in **a** (pET28-M.SacI) was subjected to EarI digestion. Sensitivity is expressed by accumulation of the 1557 bp band (1309 bp + 248 bp). *Middle and Right panels:* distinct 1055 bp PCR products carrying each site shown in **a** (M.SssI-1 or M.TaqI, respectively) were methylated *in vitro* and subjected to EarI digestion. Sensitivity is expressed by accumulation of the 700 bp band (450 bp + 250 bp). Images are representative of duplicated experiments. **(c)** Gel bands of each replicate described in **b** were quantified and expressed as percentage: 1-[digested/(digested + linearized)] in each tube. Mock is average of non-methylated DNAs (n = 6) and bars are standard error. Non methylated sites in the same molecule showed the digestion was >97% completed in each tube. (**d**) Methylation efficiency of 5,291 bp (M.Test) vector *in vivo* by T7X.MT (which carries the TaqI methyltransferase in the genome, see Supplementary Fig. 3) compared to control (same vector in T7X) and *in vitro* data (M.TaqI; similar to **c** but new replicates). T7X.MT carrying the plasmids were selected on plates supplemented with 0.3mM IPTG and grown overnight at 37 °C in liquid media + 0.2 mM IPTG for 14–18 h before DNA extraction. Bars are standard error of four biological replicates and graph is a representative image of a duplicated experiment.

To date, all *type IIS* enzymes that leave a 3nt overhang and therefore are suitable for use in our TNT-cloning system recognize either 5′CTCTTCN^▼^NNN_▲_3′ (e.g., EarI) or 5′GCTCTTCN^▼^NNN_▲_3′ (e.g., LguI) sequences^21^ (Supplementary Table 1), leaving the EarI recognition site nested within the LguI site. To overcome this limitation we assessed EarI sensitivity to different methyl groups added either within or nearby the 5′GCTCTTCN^▼^NNN_▲_3′ sequence (EarI was chosen over Eam1104I due to previous reports on methylation sensitivity^21^). We used three methyltransferases (M), M.SacI, M.SssI (2 sites) and M.TaqI to methylate, respectively, the cytosines at the positions 2/1 (forward/reverse strand), 7/8 or -1/1 and the adenines at the positions 9/6 (Fig. 3a). For this purpose, we used a 6,435 bp plasmid (pET-28-M.SacI) and different 1055 bp PCR fragments carrying at least two sites for the restriction endonuclease where at least one site would not be subjected to methylation (except for M.SssI where both sites were addressed simultaneously). Two distinct methylation sites generated by M.SssI had little (M.SssI-1) to no (M.SssI-2) effect in EarI ability to cut the modified DNA (Fig. 3b,c). On the other hand, sensitivity tests showed that M.SacI and M.TaqI inhibited the enzyme activity by 83.4% (SE ± 5.4) and 99.9% (SE ± 0.03), respectively (Fig. 3b,c). Because M.TaqI was highly capable of inhibiting digestion of DNA by EarI, we adopted this modification to design the TNT-cloning system with, a) the first nucleotide of each signature that flanks the restriction site starting with an adenine, and; b) such modification present only when EarI is the first enzyme to be used (i.e., *α* members).

To avoid the cost and time of performing *in vitro* modifications of the *α* members before cloning, we engineered the genome of the *E. coli* strain T7Express (T7X) to be capable of expressing the M.TaqI gene during its regular life cycle (Supplementary Fig. 3). Different conditions for growing the engineered strain (T7X.MT), while keeping maximum DNA methylation, were tested (Supplementary Fig. 4) and the optimal practice is shown in Fig. 3d, where 97.1% (SE ± 0.8) of the plasmid DNA extracted from T7X.MT was unable to be cut by EarI. Our results show the use of this strain is comparable to the modification levels obtained for the *in vitro* methylation. Methylated DNA extracted from T7X.MT remains stable at -15 °C for at least 11 weeks without compromising EarI/Eam1104I inhibition (Supplementary Fig. 4). There is no methylation requirement for both the *Ω* members and downstream cloning steps in the *α* members, and therefore, any construct generated using the TNT-cloning system can be transformed in the strain of choice (T7Express must be used to allow for white/blue screening). As a consequence, it is not necessary to define LguI sensitivity to methylation; however, we report a sensitivity chart for three isoschizomers in this class: BspQI, LguI and SapI (Supplementary Fig. 5). Importantly, LguI is also sensitive to M.TaqI modification and yet this is irrelevant because M.TaqI site is not present at a critical position on *Ω* members and transformation of constructs carrying the joined fragment(s) in the *α* members is not required for T7X.MT. Consequently, the T7X.MT strain is useful for propagation of the original TNT-plasmids but problematic for downstream cloning purposes.

Our results show that we successfully engineered two distinct sites to support the assembling loop presented, with LguI recognizing and cleaving at the sequence 5′GCTCTTCN^▼^NNN_▲_3′ and EarI recognizing and cleaving at the sequence 5′CTCTTCN^▼^NNN_▲_3′ (but not at the sequence 5′GCTCTT^*^CN^▼^N^*^NN_▲_3′, where T^*^/N^*^ represent a methylation of the corresponding adenines). By using the engineered *E. coli* strain our required modification is simple to implement.

### Validation and performance of TNT-cloning system

Once we defined the specificity of the restriction sites, we built all 17 TNT-vectors described in Supplementary Fig. 1; pSTART (carbenicillin resistance), *α* members (*α1A, α2, αB, αC, α1A-R, α2-R, αB-R, αC-R*; spectinomycin resistance) and *Ω* members (*Ω1A, Ω2, ΩB, ΩC, Ω1A-R, Ω2-R, ΩB-R, ΩC-R*; kanamycin resistance - see Methods for details) based on the use of M.TaqI. Several fragments were amplified by PCR or synthesized to be cloned into pSTART, becoming an element in our universal library, e.g., regulatory regions (upstream regulatory region, URR; untranslated regions, UTRs; ribozymes; secondary regulatory sequences), coding sequences (proteins; localization signals; affinity tags; functional domains), structural sequences (replication origins; repetitive DNA) and engineering scaffolds (interfering RNA, RNAi; artificial microRNA, amiR; guided RNA, gRNA; recombination sites) (Supplementary Table 2). Importantly, we subjected some of our coding sequences (CDS) to the domestication process, i.e., to screen and synonymously mutate 5′CTCTTC3′ and 5′GAAGAG3′ sites in order to avoid internal fragment cleavage during the cloning steps (Supplementary Fig. 1). However, to domesticate a fragment is not mandatory for our system (see section “*Overcoming the domestication step*”).

**Figure 4:**
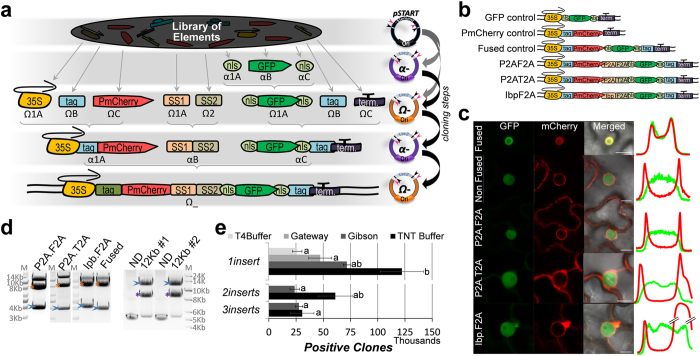
TNT-cloning system proof of concept. **(a,b)** Scheme of 10 elements joined using our approach: 35SPromoter (*35S*), lumio tag (*Tag*), PIP2mCherry (*PmChery*), different ‘self-splicing’ proteins (*SS1* and *SS2;* to be different combinations of the viral proteins P2A, F2A and T2A and the plant protein Ibp), nuclear localization signal (*NLS*), GFP (*GFP*) and 35S terminator (*Term*). Rows are assembly level where elements are first transferred from the library (pSTART) to either *α* or *Ω* vectors (gray arrows) before binary/tertiary assembly (black arrows). **(c)** Confocal image of final constructs without (*Fused*) or with different sets of SS proteins (*P2AF2A, P2AT2A* and *IbpF2A*) in tobacco leaves. Co-infiltrated PmCherry and GFP controls (*Non Fused*) represent a “maximum split” whereas fused/non-split proteins are localized in the nucleus. A representative breakdown of mCherry(red) and GFP (green) fluorescence across a nuclei section (10–18 μm) is shown: *P2AF2A* (99.7% SE ± 1.2), *P2AT2A* (94.2% SE ± 2.8), *IbpF2A* (79.7% SE ± 8.4) (see also Supplementary Fig. 6). Double bars separate a 3 fold difference zone. Scale bar, 10 μm. **(d)** Exemplification of 28 fragments from the library (pSTART) joined into 1 final 12 kb fragment after 5 cloning steps. *Fused Ω1A* (8 elements, 3.9 kb), *P2AF2A ΩB* (10 elements, 4 kb) and *IbpF2A ΩC* (10 elements, 4.1 kb) were joined by tertiary assembly (left panel) generating the 12 kb fragment in *α1A* (right panel). Asterisks, vector backbone; arrows, inserts after appropriate digestion. ND, non digested. **(e)** Ability of one and multiple fragments to be joined by different methods. Same 1 insert elements were used in T4-buffer and TNT-buffer reactions (sizes 0.25–2.4 kb). Similar 3 inserts fragments were used for the remaining reactions (T4-buffer, 0.9–1.5 kb in either *one-pot* TNT- or GoldenBraid-reactions; TNT-buffer, 1.2–2.7 kb in *one-pot* TNT-reaction; Gibson assembly, 0.8–2.4 kb). Error bars are standard error between 3 and 7 biological replicates. TNT-reactions cover both ways (*α*→*Ω* and *Ω*→*α*) and include both *aa*CTCTTC and *cc*CTCTTC *Ω*’s (see Supplementary Fig. 7). Positive clones are confirmed by PCR (16 < n < 32). Same lot of competent cells was used. Bonferroni, p < 0.01(letters).

As a proof-of-concept we used ten different DNA fragments from our library to design six final constructs expressing a set of two reporters, red (mCherry) and/or green fluorescent proteins (GFP), respectively fused to PIP2 (plasma membrane intrinsic protein^22^) and the known subcellular domains NLS (nuclear localization signal: PKKKRKVEDP^23^), with or without a “self-splicing” protein (SS) in between each reporter gene^24^,^25^ (Fig. 4a,b). To maintain maximum flexibility, the CDS cloned in the pSTART have no ‘stop codons’, which are included in the Terminators/3′UTRs. Each construct, *35S::NLS-GFP-NLS-Term (α1A)* (GFP control), *35S::tag-PmCherry-Term* (*Ω1A*) (PmCherry control), *35S::tag-PmCherry-NLS-GFP-NLS-tag-Term(Ω1A)* (Fused control) and different *35S::tag-PmCherry-SS1-SS2-NLS-GFP-NLS-tag-Term* were transformed in agrobacteria and infiltrated^26^ in tobacco leaves to confirm mCherry and GFP fluorescence (Fig. 4c, Supplementary Fig. 6). The combinations of SS1-SS2 were *P2AF2A* (*ΩB*), *P2AT2A* (*Ω1A*) or *IbpF2A* (*ΩC*) (different peptide 2A^24^; Impatiens balsamina peptide, cleaved in plants^25^; see Methods for detailed assembly description). As expected, the Fused control had the same expression pattern as *35S::NLS-GFP-NLS-Term (α1A)*, being nuclear localized, and the PmCherry control localized to the karyotheca and plasma membrane (Fig. 4c, Supplementary Fig. 6a). The constructs carrying the SS clusters should mimic the clean separation of signals observed when GFP control and PmCherry control are co-infiltrated (Fig. 4c; non-Fused control) indicating an effective split between both reporters. The most efficient split was observed when either *P2AF2A* (99.7% SE ± 1.2) or *P2AT2A* (94.2% SE ± 2.8) were used and less definitive cellular split efficiencies were observed when *IbpF2A* (79.7% SE ± 8.5) was used (Fig. 4c, Supplementary Fig. 6b). These results demonstrate the TNT-system is functional and multiple coding sequences can be coupled into one mRNA to efficiently undergo independent translation.

To evaluate the effect of fragment length on the efficacy of our system we took the Fused control (≈4 kb), the *P2AF2A* cluster (≈4 kb) and the *IbpF2A* cluster (≈4 kb) in *Ω1A, ΩB* and *ΩC*, and used a tertiary assembly to generate a ≈12 kb fragment in *α1A* (Fig. 4d). Additionally, we developed an efficient protocol along with an improved buffer system (called TNT-Buffer; 50 mM Tris-HCl pH7.5, 2 mM DTT, 10 mM MgCl_2_, 1 mM ATP and 2% PPG) that allowed EarI and LguI enzymes to work well in combination with T4 DNA ligase in a “*one-pot-reaction*” (Fig. 4e). When using 75 ng of entry plasmid DNA (75 ng each entry plasmid for multiple elements assembling) and 50 ng of destination vector (TNT-members *α, Ω* or pSTART) a variety of fragment sizes (from 36 bp to 2.7 kb) could be efficiently cloned. The average number of positive clones retrieved from 1, 2 or 3 elements cloning using the TNT-buffer were, respectively, 12.2 × 10^4^ (SE ± 16.2%), 6.1 × 10^4^ (SE ± 25.4%) and 3.0 × 10^4^ (SE ± 35.2%), if full ligation reaction is applied. Importantly, the accuracy, which is the number of positives clones among all clones retrieved in a plate, were ≈100%, ≥83.3% and ≥81.2% when 1, 2 or 3 elements were being cloned, respectively. Differently, the analogous reactions performed using the regular T4 DNA ligase buffer, the LR Reaction from the Gateway system or the isothermal (Gibson) assembly for cloning 1 element retrieved, respectively, 2.6 × 10^4^ (SE ± 16.3%), 4.7 × 10^4^ (SE ± 22.3%) and 7.1 × 10^4^ (SE ± 4.4%) positive clones, if full ligation reaction is applied. We followed the manufacturer’s instructions for each method and all three showed ≈100% accuracy. The EarI/LguI/T4 ligase enzymes concentration were very important, especially for accuracy and a standard 10 μl final volume TNT-reaction includes 40 U of T4 DNA ligase plus either 5 U of EarI or 0.5 U of LguI. Regardless of the buffer system, the LguI enzyme showed some promiscuity over the 5′*aa*CTCTTC3′ EarI site originally included in the *Ω* vectors and four point mutations upstream of the biding site (from *aa* into *tt, gt* and *cc*) were tested and finally changed to 5′*cc*CTCTTC3′ in order to achieve such efficiencies (Supplementary Fig. 1, Supplementary Fig. 7). These results show other cloning strategies available scored less efficient than the TNT-system and the TNT-buffer is up to 18-fold more efficient than the T4 DNA ligase buffer (Fig. 4e). The key component of our buffer is a branched polyethylene glycol (PPG) that appears to allow efficient digestion/ligation while maintaining efficient exchange of inserts between vectors. Since the isothermal (Gibson) assembly^12^ also allows for multiple fragments cloning, we also compared 2 and 3 elements cloning using both methodologies -- the *one-pot-reaction* in TNT-buffer (50 cycles of: 34 °C for 45 sec and 16 °C for 4.5 min) or the 1 h Gibson assembling reaction (at 50 °C) (Fig. 4e). Both methods performed well for 2 or 3 elements cloning and the TNT-buffer respectively retrieved 6.1 × 10^4^ (SE ± 25.4%; ≥83.3% accuracy) and 3.0 × 10^4^ (SE ± 35.2%; ≥81.2% accuracy) positive clones while the Gibson assembling respectively retrieved 2.3 × 10^4^ (SE ± 18.2%; ≈100% accuracy) and 2.7 × 10^4^ (SE ± 12.1%; ≈100% accuracy) positive clones, both when full ligation reaction is applied. Lastly, the regular T4 DNA ligase buffer retrieved 0.05 × 10^4^ (SE ± 5.9%) positive clones with ≥35.5% accuracy during 3 elements cloning when full ligation reaction is applied (Supplementary Fig. 7).

Taken together, our results show that the TNT-cloning system is a powerful tool for flexible, rapid and all-in-one efficient assembling of various DNA fragments requiring no homology or linker/adaptors between fragments. The ≈12-kb proof-of-principle fragment noted above is an example of how 28 fragments from the library could be easily designed and joined into a single insert using 5 cloning steps. Because each construct generated is ready to be used as an entry clone for future assembling (and as an element in the library if cloned in the pSTART, Supplementary Fig. 1), such system is also remarkably versatile and convenient, requiring minimal to no re-cloning.

### Overcoming the domestication step

**Figure 5:**
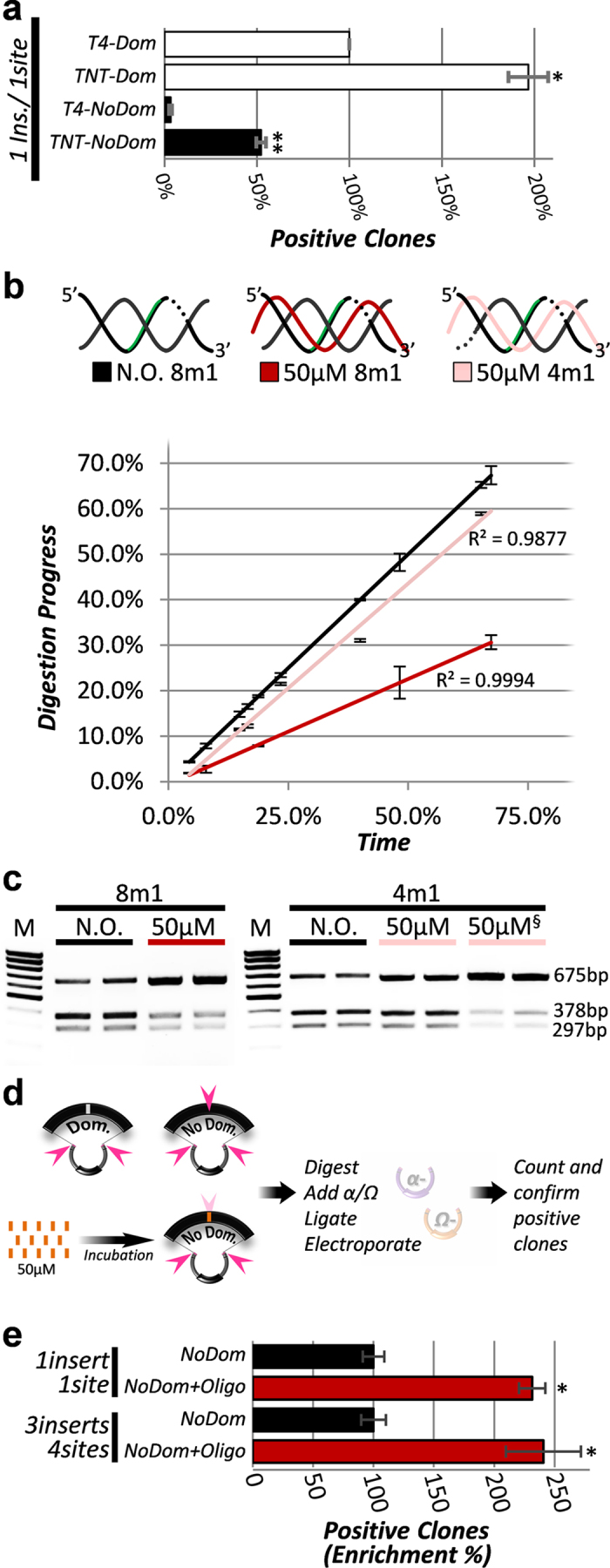
Strategies for overcoming the domestication step. (**a**) T4- and TNT-buffers ability to generate positive clones from fragment bearing (NoDom) or not (Dom) internal 5′GCTCTTC3′ site. Standard TNT-reaction was performed in both panels (50 cycles of 34 °C for 45 sec and 16 °C for 4.5 min) in duplicates and graphs are a representative image of a duplicated experiment (covering *Ω* members 1A, B and C). T4-NoDom (3.3% SE ± 0.7) and TNT-NoDom (52.3% SE ± 2.6). “Dom” Bonferroni, p < 0.05(*), “NoDom” Bonferroni, p < 0.01(**). **(b)** Digestion progress over time using the duplex 8 m1 without oligo (black line), duplex 8 m1 + 50 μM oligo (dark red line; potential triplex) or duplex 4 m1 + 50 μM oligo (pink line). The oligo specifically delays the digestion of desired 5′(G)CTCTTC3′ site. Lines are linear trend (R^2^ values shown) of ten (N.O.) and five (8 m1 and 4 m1) time points done in duplicates (see also Supplementary Fig. 8). **(c)** Gel image representative for **b** at 64.3% (SE ± 1.1%) digestion progress. Efficient oligo-dependent inhibition keeps the 675 bp PCR band intact (8 m1 inhibition, 45.4% SE ± 0.9; 4 m1 inhibition, 9.8% SE ± 0.5). 50 μM^§^ represents the 4 m1 template incubated with the 26nt-Acr oligo (inhibition = 48% SE ± 4.2). **(d)** Scheme of the “*BlindSpot”* protocol. Oligos were incubated with appropriate plasmid DNA carrying 8m1 (NoDom) fragments and subjected to partial digestion, ligation to linearized vector and transformation into *E. coli*. Colonies were then counted, confirmed for positive clones and recorded. **(e)** Enrichment for positive clones during both way cloning (EarI/LguI) of one (top) and multiple (bottom) non-domesticated fragments upon incubation with a 26nt DNA oligo (*BlindSpot* protocol). Since absolute number of clones equals number of positive clones (accuracy ≈100%) during one fragment cloning, data represent real increase in colonies in the plate. Number of internal 5′(G)CTCTTC3′ sites are indicated. Ligation was performed using T4-Buffer and error bars are from three biological replicates, Bonferroni p < 0.05(*). Graph is representative of a duplicated experiment. Note that 3ins/4sites retrieved no positive clones under a regular TNT-reaction (data not shown) but only when the “*BlindSpot”* protocol, which represent a partial digestion, was applied (see Methods).

Available *type IIS* restriction enzyme-based systems also hinder its application due to mandatory mutation steps necessary before DNA elements can be cloned and assembled. One solution already mentioned above is to domesticate an element by changing a 5′CTCTTC3′ site(s) while maintaining its functionality. However, many elements cloned are not CDS and therefore this strategy cannot be applied. The unique TNT-buffer efficiently clone elements with a internal 5′(G)CTCTTC3′ site (Fig. 5a), however, tertiary assembling involving non-domesticated inserts were complex and positive clones were not recovered. Therefore, we utilized the ability of DNA to form triplexes^27^,^28^,^29^ in an effort to change the DNA-enzyme interactivity^30^ and inhibit the *type IIS* digestion progress by masking specific 5′(G)CTCTTC3′ sites using oligonucleotides.

To design such oligos, we adopted the Reverse-Hoogsteen orientation^27^, which allows for all four nucleotides to be part of the triple helix. Initially, we combined the ability of the intercalating dye acridine (Acr) to stabilize triple helixes with the modified oligonucleotide DNA/BNA^NC^ (2′-*O*,4′-*C*-aminomethylene bridged nucleic acid), which has stronger binding affinity than DNA oligos (14 bp DNA/BNA^NC^ Tm = 82.5 °C) and is more capable of forming triplexes at physiological pH (7.0–8.3)^29^. Increasing amounts of DNA/BNA^NC^ oligo showed oligo-dependent inhibition of the digestion progress over the 675 bp PCR product template ‘8 m1’, suggesting inhibition of enzyme activity by a potential triplex formation (Supplementary Fig. 8). On the other hand, this DNA/BNA^NC^ oligo was not able to discriminate 5′mismatches (3 in total) as observed by similar inhibition over the template ‘5 m2’, showing this oligo does not differentiate small mismatch changes as those found between internal and vectorial 5′(G)CTCTTC3′ sites. Therefore, we decided to test two regular DNA oligonucleotides (26 nt and 26 nt-Acr) covering 11 nt upstream and 8 nt downstream of the 5′(G)CTCTTC3′ site. A “digestion-progression curve” using LguI on the non-domesticated templates 8 m1 (0 mismatches) and ‘4 m1’ (4 mismatches) in the absence or presence of 50 μM of the 26-nt DNA oligo were performed to understand the kinetics involved in the digestion inhibition (Fig. 5b, Supplementary Fig. 8). We demonstrate the regular unmodified 26nt DNA oligo inhibited LguI activity at the desired site by 75.9% (SE ± 0.9%; 8 m1 template) and only 8.3% (SE ± 1.6%) when 4 mismatches (4 m1 template) were present, yielding a ‘inhibition coefficient’ of 67.6% (Fig. 5b,c). EarI had a slightly smaller inhibition coefficient of 49.9%, requiring a higher number of mismatches to discriminate specific 5′CTCTTC3′-containg sequences (Supplementary Fig. 8). Expectedly, the 26-nt-Acr oligo showed stronger inhibition but intensely compromised specificity (Fig. 5c, Supplementary Fig. 8). These *in vitro* data were validated by cloning alternate single and multiple fragments containing up to 4 internal 5′(G)CTCTTC3′ sites into different *α* and *Ω* vectors. Compared to the 7 m1 domesticated (Dom) element, the cloning of a 8 m1 non-domesticated (NoDom) element reduced the number of positive clones to 32.2% (SE ± 8.9%) but to only 74.0% (SE ± 7.3%) when the 26-nt oligo was previously incubated with the template plasmid, enriching the ability to recover positive clones by 231.5% (SE ± 10.9%) (Fig. 5e). Similarly, when a tertiary assembly is performed using three elements in a total of four internal 5′(G)CTCTTC3′ sites (template 8 m1), the somehow equivalent number of positive clones had increased accuracy when previously incubated with the 26-nt oligo, going from 31.2% (SE ± 4.4) to 77.1% (SE ± 1.4), enriching the ability to recover positive clones by 240.8% (SE ± 31.1%) (Fig. 5e). Combined, these results show the oligo incubation efficiently and specifically created a manageable “blind-spot” to minimize enzyme activity over chosen 5′(G)CTCTTC3′ sites while leaving the remaining (vectorial) 5′(G)CTCTTC3′ sites reliably available for LguI/EarI to recognize and digest. Thus, the TNT-cloning system is excused from the domestication process and the probability of finding a fragment unsuitable for cloning are exceedingly rare.

## Discussion

Building the first synthetic organism leveraged a set of tools for assembling DNA fragments both *in vivo* and *in vitro* that were effective^4^,^12^. These efforts, and the construction of subsequent genomes and specific gene cassettes, have utilized a linear approach that relies on sequence homology, limiting reuse, multi-combinatorial distribution and shuffling of key fragments necessary for iterative studies of genetic circuits. An alternative would be to adopt systems based on *type IIS* enzymes^18^,^31^; however, current methods have limits in efficiency and efficacy. We associated an efficient buffer system with the placement of methyl groups in the *type IIS* enzymes binding site to generate two recognition sites for two distinct enzymes creating an innovative and flexible cloning platform, allowing for multiple elements (up to 3 at once) to be combined from a single universal library in a one-pot reaction with high efficiency and high fidelity (Figs 1, 2, 3, 4). The ability to keep ORFs in frame by using cloning signatures that bear three nucleotide tag allowed us to include all cloning fragments, as CDS pieces, into a single universal library and, therefore, simplify assembling by orderly ‘picking and mixing’ the elements of interest. In this approach inversions were, and can be, easily performed by merely swapping the destination vector with its corresponding “R” version. Similarly, relocation of fragments was easily performed by rearranging intermediate cloning products rather than starting from the beginning of the process. Such characteristics are responsible for the main advantages found in the TNT-cloning system compared to previous restriction enzyme-based methods (Table 1). Notably, the TNT-system rely on three nucleotides overhang that is finally incorporated in the constructs while some homology based methods, e.g. isothermal (Gibson) assembly, provide seamless joining of fragments. However, elements released from the universal library are always compatible with isothermal (Gibson) assembly due to a 5′ extension, rather than a 3′ extension, left by the EarI/LguI enzymes and therefore well-suited for scarless cloning.

The features found in the TNT-cloning system are key for establishing a easily transferable platform for quick determination of qualitative and quantitative gene fragment interactions that will have to be performed in studies involving gene sets and gene networks^8^. Currently, the validation of such networks and the reproducibility of data are limited by the inability of building various compatible multigene constructs from one flexible universal platform, requiring multiple methods to be adopted through a labor intensive pipeline^32^. The optimized TNT-cloning system and buffer, overcome such limitations providing a common platform for different elements from a single universal library to be orderly combined into 1 insert after a minimal number of cloning steps in a matter of days.

Within the context of synthetic biology, an important aspect for studies in regulatory networks and pathway engineering is the need of numerous regulatory sequences that may be incompatible with current cloning systems and/or limited in number, e.g. promoters. Here, we were able to provide a protocol that is greatly capable of cloning fragments bearing internal 5′(G)CTCTTC3′ sites, supporting the use of such regulatory sequences (Fig. 5). Whether the assembly efficiency is compromised when large multigene constructs involving many undomesticated internal sites are involved remains to be tested. Such factor could also limit the exchangeability of the constructs; however, our approach is affordable and straightforward allowing for use of certain elements inapt for mutagenesis.

In addition, we demonstrate the programming of polycistronic mRNAs, a valuable tool for managing bistability/hysteresis^33^ in genetic circuits as well as overcoming promoter shortage in multi-gene constructs (Fig. 4). We clustered different peptide 2As to overcome flaws found when only one sequence is used^24^ by assuming a simple probability test should be applicable (if one copy gives 20% flaw, for example, two copies should reduce such number to 4%). We showed that such clustering corroborates our predictions, as *P2AT2A* and *P2AF2A* constructs gave almost flawless split between two CDS while their sole use show imperfect split in several cellular backgrounds^24^,^34^. This observation suggests that 10 genes can be grouped into one mRNA with approximately 97% [=(0.997)^9^] efficiency of individually translated transgenes. However, the maximum number of genes capable of being practically clustered and the protein longevity due to the *N-rule* turnover^35^ (first amino acid of the nascent peptide after efficient split is a proline) remains to be addressed. Nevertheless, the advantages intrinsic to polycistronic mRNAs further support the development of a methodology that allows an *endless assembly* with CDS compatibility.

In sum total, we have developed a new cloning platform, enabling gene circuits and pathway engineering and allowing for virtually any DNA fragment to be quickly, reliably and flexibly clustered and shared. Such a platform provides essential steps required for synthetic biology studies to progress faster and with high fidelity, even as DNA synthesis costs drop. Because of the ease of transferability of the developed platform, our system also contributes to the universality expected by the synthetic biology community and highlighted by several recent papers^14^,^19^,^36^,^37^.

## Methods

### Methylation tests

Type II cytosine-5 DNA methyltransferase protein sequence from *Streptomyces achromogenes*, which recognizes and modifies the sequence 5′GAGCTC3′ (M.SacI; GenBank AAC97118.1), was reverse translated, synthesized (Supplementary Table 3), cloned in pET28 (pET28-M.SacI) by Gibson assembly (NcoI-SalI sites), transformed in T7Express and induced according to vector/strain suggested protocol (4 h, 0.5 mM IPTG). Expression of the ≈43 kDa protein was confirmed by protein gel (data not shown) and a second fraction of the same culture had the pET28-M.SacI plasmid extracted, quantified and 1 μg was subject to incubation with BspQI, LguI, SapI or EarI in duplicates on manufacturer recommended buffer. Digestion ran for 1 h at 37 °C (except for BspQI, where 50 °C were used) using 5 U of each enzyme (except SapI, where 10 U was used) in 20 μl reaction volume. The reactions were stopped and loaded in agarose gel. Bands were quantified by ImageJ software (area tool after plotting lanes) and organized using Excel. A non-methylated control was always included, and for M.SacI and M.TaqI (see below) sites non-subjected to methylation inside each tube, were also used to check full restriction enzyme activity. “Digestion inhibition” was a direct measurement of the digested bands divided by total band intensities (digested plus non-digested) and “Methylation efficiency” was calculated by 1 minus “Digestion inhibition”. For M.SssI assays, a 1055-bp PCR product, using the pET28-M.SacI plasmid as template, was amplified (using the primers TaqI-Fw and TaqI-Rw), purified, quantified and incubated with methyltransferase as manufacturer instructions (NEB). In this case, there are 92 sites for M.SssI (5′CG3′), which counts for ≈25 μM of substrate in a 20 μl reaction if 1 μg of DNA is used. In this case, to achieve complete methylation, 1 μl of enzyme (4 U) is recommended by the manufacturer to fully methylate 4 μg of such template in 20 μl reaction supplied with 640 μM SAM for at least 2 h at 37 °C; our reactions ran for 4 h under these conditions. Methylated DNA was purified and 400 ng used for *type IIS* assays in duplicates and “Digestion inhibition” and “Methylation efficiency” were assessed as described above. Both sites shown in Fig. 3 are present simultaneously in the fragment and could be addressed in the same reaction by selecting the appropriate bands for quantification. For M.TaqI assays, two PCR products using the pET28-M.SacI plasmid as template were obtained (using the primers TaqI-Fw and TaqI-Rw1.1 and TaqI-Fw1.1 and TaqI-Rw), purified, quantified, diluted at least 1000 fold and mixed together in an equimolar ratio for a secondary PCR (30 cycles) using only TaqI-Fw and TaqI-Rw to generate the 1055 bp fragment with an internal M.TaqI site as shown on Fig. 3. The 1055-bp secondary product was then purified, quantified and incubated with methyltransferase as manufacturer instructions (NEB; except we increased incubation time to 4 h). Methylated DNA was purified and 400 ng used for *type IIS* assays in duplicates and “Digestion inhibition” and “Methylation efficiency” as described above. After the screening in duplicates, the M.TaqI results were confirmed by other 4 biological replicates for EarI only (Fig. 3). For *in vivo* assays, using M.Test plasmid transformed in T7X.MT, two separate colonies were tested on each condition shown on Supplementary Fig. 4 and the best condition (cultures grown on plates with 0.3 mM IPTG right after original transformation and 0.2 mM of IPTG on liquid media overnight grown at 37 °C) were reproduced for other 4 new colonies (Fig. 3). Experiment was later reproduced once, with 3 biological replicates and M.Test DNA was then kept at −15 °C and re-accessed after 3 weeks (data not shown) and after 11 weeks, in which Eam1104I was also included.

### TNT-family of vectors

All primers, GBlocks and gene cassettes were commercially synthesized and used in this study are listed on Supplementary Table 3. Nucleic acid manipulation followed the general guidelines described in^38^. DNA preparation was performed by either traditional phenol:chloroform extraction or DNA extraction kit (5PRIME #2300010). The pSTART is a pUC19-backbone vector, which carries the ampicillin/carbenicillin resistance gene, and was built domesticating EarI sites (5′CTCTTC3′) by using Gibson assembly^12^ to join the PCR products of primers 1) pUPD-FW1 and pUPD-RW1 (188 bp), 2) pUPD-FW2 and pUPD-RW2 (149 bp), 3) pUPD-FW3 and pUPD-RW3 (301 bp), 4) pUPD-FW4 and pUPD-RW4 (1838 bp) and 5) pUPD-FW5 and pUPD-RW5 (274 bp). The “ΔM15ω-peptide” was separately amplified from *E. coli* DH5α using the primers pUPD-RW3.1 and FW_adap and assembled into domesticated pSTART linearized by PCR using the primers pUPD-FW3.1 and pUPD-RW5. For the M.Test vector, used on M.TaqI assays in T7Express and T7X.MT, the pUPD-RW5-M_Test and pUPD_adap_met.test-FW were used instead of pUPD-RW5 and FW_adap, respectively (creating the M.TaqI site 5′TCGA3′). The backbone of the binary vector pPZP200^39^ (positions 1 to 6495 bp), plus a spectinomycin resistance cluster, were domesticated at different 5′CTCTTC3′ sites using the primers αΩvector-FW and EarI-RW1 (1132 bp), EarI-FW1 and EarI-RW2 (2699 bp), EarI-FW2 and EarI-RW3 (493 bp), EarI-FW3 and EarI-RW4 (2866 bp), EarI-FW4 and EarI-RW5 (234 bp) and EarI-FW5 and αΩvector-RW (817 bp). PCR products were purified, mixed in equimolar ratio and re-amplified using the primers αΩvector-nested-FW and αΩvector-nested-RW (8080 bp band). The 8080-bp band was re-amplified with primers αΩvector-FW and αΩvector-RW to generate the α-backbone segment. The α version had the appropriate primer pairs α1A-Fw and α1A-Rw, α2-Fw and α2-Rw, αB-Fw and αB-Rw, αC-Fw and αC-Rw, α1R-Fw and α1R-Rw, α2R-Fw and α2R-Rw amplifying the reporter ΔM15ω from pSTART during a first PCR with each product followed by a secondary PCR with the primers PCR2_to_αVector-Fw and PCR2_to_αVector-Rw to create the 18-bp overlap needed for joining each segment by Gibson assembly^12^ to the α backbone. First, we built α1A, and upon sequencing of CDS present in this backbone plus the T-DNA borders, the remaining members, α2, αB, αC, α1A-R, α2-R, were assembled. Similarly, the appropriate primer pairs, Ω1A-Fw and Ω1A-Rw, Ω2-Fw and Ω2-Rw, ΩB-Fw and ΩB-Rw, ΩC-Fw and ΩC-Rw, Ω1R-Fw and Ω1R-Rw and, Ω2R-Fw and Ω2R-Rw, were used to amplify the reporter ΔM15ω from pSTART during a first PCR with each product followed by a secondary PCR with the primers PCR2_to_ΩVector-Fw and PCR2_to_ΩVector-Rw to create the 18-bp overlap needed for joining each segment, by Gibson assembly, to the α backbone creating the plasmids Ω1Aabb, Ω2abb, ΩBabb, ΩCabb, Ω1A-Rabb, Ω2-Rabb, where “abb” indicates α backbone. These Ω members then had the spectinomycin marker (aminoglycoside adenylyltransferase) switched to kanamycin (aminoglycoside phosphotransferase) by linearizing each member using the primers KStrat2_TNT-FW and KStrat2_TNT-RW (9351 bp) to be joined by Gibson assembly with fragment 1 amplified with Kan_to_O-FW2 and KStrat2_TOP-RW (1496 bp) and fragment 2 amplified with KStrat2_TOP-FW and Kan_to_O-RW1 (384 bp), both fragments from pENTR-D-TOPO. Later, the Ω’s had the point mutation, noted in Supplementary Fig. 7, adjusted by linearizing the vectors with PstI or PmeI and partially digesting with LguI for assembling with a double strand oligo (named leftCC-FW/RW or rightCC-FW/RW) covering the same sequence (positions 83–142 bp, when PstI was used, or 3328–3394 bp, when PmeI was used) with the point mutation from 5′*aa* to 5′*cc* being located at positions 108–109 bp and/or 3361–3362 bp (Ω1A versions 5′*tt* and 5′*gt* at the 3361–3362 bp positions were also created and tested, data not shown). Importantly, this change was performed on all versions, however, only at those sites that bear two signatures side-by-side (Supplementary Fig. 1). Lastly, the versions αB-R, αC-R, ΩB-R and ΩC-R were implemented by digesting the α1A and Ω1A vectors at the PstI and PmeI sites and assembling the purified backbone to three GBlock fragments, having one in common (LacZw-central-gb) and the remaining specific for each vector created (alphaBR-gb left, alphaBR-gb right, alphaCR-gb left, alphaCR-gb right, omegaBR-gb left, omegaBR-gb right, omegaCR-gb left, omegaCR-gb right) by Gibson assembly. All vectors created without exceptions had the signatures confirmed by sequencing before undergoing tests. Primers pUPD-seqFW and pUPD-seqRW (for pSTART) or primers TNT-αΩ-seqFW and TNT-αΩ-seqFW (for any α and Ω members) were used to sequence inserts and diagnose constructs by colony PCR. Entry elements relevant to this work (Supplementary Table 4) were either synthesized, amplified (green fluorescent protein, TNT-GFP-FW/RW; PIP2 fused to mCherry, TNT-PmCherry-FW/RW; 35S promoter, TNT-35SProm-FW/RW; and 35S terminator, TNT-35STerm-FW/RW) from general templates or simply dimerized (100 pmol in 50 μl of 1× PCR buffer for 95 °C 5 min and then 85 °C to 45 °C every 5 °C, 5 min each) using FW and RW primers (Lumio_tag, NLS, P2A, T2A, F2A and Ibp) before being assembled (1 μl of dimerized oligos) in the pSTART by Gibson assembly. Some primers used to clone other elements tested in our entry vector pSTART, but not used further in this work, are listed for reference (TNT-Cas9-FW/RW1-5, partial domestication; GUS reporter, rGUS-FW/RW; 35S::hygromycin-F2A-CodA-Terminator, HCC selectable marker, Hig-CodA-FW/RW; Luciferase reporter, Luc+_pUPD_FW/RW; DNA 2.0 CPB-38-441 vector, CircRep-FW/RW).

### Library construction (pSTART) and constructs diagnosis

Primers, to clone fragments by either restriction/digestion or Gibson assembly, were designed as 5′ACATGCAGCTCTTCCACCN_(20)_3′, where N is the fragment of interest sequence forward (signature 1 is underlined), and as 5′CGAGGAAGCTCTTCCATCN_(20)_3′ for reverse strand (signature 2 is underlined), as long as TM of N_(20)_ >50 °C. Otherwise, number of base pairs was increased over 20 nt until at least 50 °C of TM was reached (https://www.idtdna.com/calc/analyzer) (see Supplementary Fig. 1). Multiple PCR products were purified and combined by Gibson assembly. All PCR reactions were performed using Phusion DNA polymerase (Thermo Scientific) according to suggested protocol (DMSO was added accordingly if amplicon was longer than 1.5 kb). Qiagen TAQ DNA polymerase diluted 10 fold was used for diagnosis through colony PCR and the remaining settings were according to suggested protocol. Briefly, colonies were picked from the agar plate and diluted in 10 μl of water in 96 well plates and 1 μl was used for PCR in 10 μl final volume. TM used was always 56 °C for 20 sec and extension was always 72 °C for 1 min; always 40 cycles. Positive clones had the remaining 9 μl (5 μl if colony PCR was performed in parallel to culture growth) inoculated in appropriate media (LB + chemicals). Every insert in the library was sequenced. First levels of complex assemblies shown in Fig. 4 were fully sequenced. Clones also checked by restriction digestion are noted in the text.

### pSTART entry clones

Supplementary Table 2 includes the entry vectors (pSTART) relevant to this work. The insert sequences were grouped in the Supplementary Table 4 as follows: d35S_h-h, PmCherry, Lumio, RGR gene^40^, P2A, T2A, Cas9*, F2A, Ibp, GFP, 35SProm, 35STerm, NLS, NosProm, GUS, HCC (Hygromycin-CodA), Kan-ORF, 8 m1*, 7 m1*, 5 m2*, 4 m1*, CircRep, 8 m2*.

### Detailed assembly steps

Once an element is cloned in pSTART, which receives and releases the desired fragments with either enzyme, it is transferred and further combined in either *alpha* (*α*) or *omega* (*Ω*) members, which receive fragments upon cleavage with EarI/LguI and release fragments upon cleavage with LguI/EarI, respectively (Supplementary Fig. 1). Upon digestion of each plasmid, a set of “signatures” that were specifically arranged to direct and orient the desired fragments are exposed. The signatures “1” and “2” are always flanking the inserts released from pSTART and are always used to join the final constructs into any *α* or *Ω* member. At the same time, the signatures “3” and “4” will be used by a specific member of each family (*α* and *Ω*) to join fragments between themselves, two fragments at once (binary assembly) using the members *α1A* and *α2* (or *Ω1A* and *Ω2*) and three fragments at once (tertiary assembly) using the members *α1A, αB* and *αC* (or *Ω1A, ΩB* and *ΩC*). To change the fragment orientation (sense or anti-sense) simply switch the chosen *α* or *Ω* version for its respective “R” version during the cloning step. We first, at the *α-*level, had the GFP transferred from the library (pSTART) to *αB* and the NLS to *α1A* and *αC*. These clones were joined in a tertiary assembly in *Ω1A* generating the *NLS-GFP-NLS (Ω1A)* construct. Secondly, at the *Ω* level, the 35S promoter (35S), the Lumio tag (Tag) (Invitrogen), the PIP2 fused to mCherry (PmCherry), different clusters of P2A-Ibp (SS1-2) and the 35S terminator (Term) were transferred to *Ω1A, ΩB, ΩC, Ω1A/Ω2* and *ΩC*, respectively. Third, again at the *α* level, the 35S (*Ω1A*), Tag (*ΩB*) and PmCherry (*ΩC*) were joined in a tertiary assembly in *α1A* generating the construct *35S::tag-PmCherry (α1A)*; the SS1 (*Ω1A*) and SS2 (*Ω2*) were joined in a binary assembly in *αB* generating the construct *SS1-SS2 (αB)*; the *NLS-GFP-NLS (Ω1A), Tag (ΩB)* and *Term (ΩC)* were joined in a tertiary assembly in *αC* to generate the construct *NLS-GFP-NLS-tag-Term* (*αC*). Finally, again at the *Ω* level, the *35S::tag-PmCherry* (*α1A*), different combinations of the SS1-SS2 (*αB*) and the *NLS-GFP-NLS-tag-Term* (*αC*) were joined in a tertiary assembly in different *Ω*s generating the construct *35S::tag-PmCherry-SS1-SS2-NLS-GFP-NLS-tag-Term*, where *SS1-SS2* means *P2AF2A* (*ΩB*), *P2AT2A* (*Ω1A*) or *IbpF2A* (*ΩC*) (different peptide 2A; Impatiens balsamina peptide, cleaved in plants^25^). In parallel, the *35S::tag-PmCherry (α1A)* and *NLS-GFP-NLS-tag-Term* (*α2*) were joined in a binary assembly in *Ω1A* generating the *35S::tag-PmCherry-NLS-GFP-NLS-tag-Term* (*Fused* control). Lastly, 35S (*Ω1A*), *NLS-GFP-NLS (ΩB)* and *Term* (*ΩC*) were joined in a tertiary assembly in *α1A* generating the *35S::NLS-GFP-NLS-Term* (*α1A*) (GFP control); the *35S::tag-PmCherry* (*α1A*) and Term (*α2*) were joined in a binary assembly in *Ω1A* generating the *35S::tag-PmCherry-Term* (*Ω1A*) (PmCherry control). All assembly reactions were performed at either 1 h at 34 °C or following a standard TNT-reaction (see below). Constructs details and annotation are depicted in Supplementary File 1.

### TNT-Buffer and the standard TNT-reaction

We tested several conditions for BspQI, EarI, LguI and SapI enzymes in order to tune our *one-pot* reaction conditions. We found the 10 mM DTT from T4 DNA ligase buffer to inhibit EarI activity and that excessive amounts of NaCl (>50 mM) inhibited LguI. BSA in a reaction increased the number of false positives (data not shown). We found the best DNA concentration to be ≈75 ng (versus 100 ng and 125 ng; 75 ng each for multiple fragment assembling) insert plasmid(s) at the range of 0.25–2.5 kb and ≈50 ng (versus 15 ng, 25 ng and 50 ng) of TNT-members *α, Ω* or pSTART. Subsequently, we found PPG to increase the number of positive colonies and allowed us to reduce the incubation time for digestion/ligation while keeping higher efficiency than the T4 DNA ligase buffer. Ideal concentration of PPG is between 0.5% and 2%. Therefore, what we called the “TNT-Buffer”, used in this work has the following formulation at 1×: 50 mM Tris-HCl (pH7.5), 2 mM DTT, 10 mM MgCl_2_, 1 mM ATP and 2% PPG (which was added right before reaction setup from a 20% stock in water). 5× buffer was stored at −20 °C. The EarI/LguI/T4 ligase enzymes concentration are extremely important especially for accuracy (number of positive clones) and a standard TNT-reaction, set up on TNT-Buffer, includes 40 U of T4 DNA ligase and either 5 U of EarI or 0.5 U of LguI, followed by incubation at ‘34 °C for 45 sec and 16 °C for 4.5 min’ for 50 cycles. If only one fragment is being cloned (or linearized destination vector is used for binary/tertiary assemblies) reaction can be performed at 34 °C for 1 h, albeit number of positive clones is reduced. All reactions were performed in 10 μl final volume and diluted 1–10 fold or 1–50 fold when *α* or *Ω* members were used as destination vectors, respectively, before taking 1 μl to transform electrocompetent cells (e.g. Fig. 4 where efficiency was ≈10^9^ cfu/μg of Puc19 plasmid). Final number of positive clones, shown in graphs, was then calculated as: total number of clones in a plate times dilution times accuracy. For general reference, GoldenBraid reactions used here followed 50 cycles of: 37 °C for 2 min and 16 °C for 5 min.

### *BlindSpot* protocol for cloning non-domesticated fragments

For non-domesticated fragments, a regular TNT-reaction was used for single fragment cloning (we have not tested fragments that would leave signatures 1, 2, 3, 4, 1R or 2R upon cleavage of internal site). For binary and tertiary assemblies involving non-domesticated fragments, we developed the *BlindSpot* protocol – i.e., fragments (≈150 ng each rather than ≈75 ng each) were first incubated with 50 μM oligo (design details below) for 1 h in each temperature 45 °C to 12 °C every 3 °C, usually overnight, in an alternate buffer (50 mM Tris-HCl pH 5.8, 75 mM NaCl, 10 mM MgCl_2_, 2 mM DTT) in 4 μl final volume. Following, the addition of 6 μl of a second buffer (50 mM Tris-HCl pH 6.3, 10 mM MgCl_2_, 2 mM DTT) containing either 5 U of EarI (for 5 min, ≈60–65% digestion progress) or 1.5 U of LguI (for 15 min, ≈55–65% digestion progress) completed the reaction volume to 10 µl, which was incubated at 25 °C before being directly heated at 80 °C for 20 min. After cool down, 2 μl were used to set up a standard TNT-reaction using either T4 DNA ligase buffer (Fig. 5, NoDom) or TNT-Buffer. For the initial screening and digestion curve (Fig. 5 and Supplementary Fig. 8), several incubation times for digestion were used and reactions were stopped with loading dye (NEB), loaded on agarose gel and analyzed similarly to what is described for the methylation assay. Since the control samples, which carry a non-domesticated fragment, showed some positive clones (Fig. 5e, NoDom), a partial digestion in these conditions was determined to be sufficient to generate the desired construct, reducing the time frame from ≈12 h (if incubation with oligo is performed) to ≈1 h. However, for maximum efficiency of complex targets, this affordable protocol more than doubled the ability to build 3 fragments assembly involving non-domesticated fragments.

We failed to obtain efficient inhibition with a regular 15 nt and 22 nt DNA oligos designed in both directions (15 ntW-H.TFOs1, 22 ntW-H.TFOs1, 15 ntRvH.TFOs1, 22 ntRvH.TFOs1, 15 ntW-H.TFOs2, 22 ntW-H.TFOs2, 15 ntRvH.TFOs2, 22 ntRvH.TFOs2, data not shown). However, we were able to show that a 26 nt DNA oligo designed to cover 11 nt upstream of LguI/EarI site and 8 nt downstream (which covers the cleavage site) in the same orientation as the 5′GCTCTTC3′ site (if the sense sequence gives the 5′GAAGAGC3′, use the anti-sense sequence for designing the oligo) inhibited both enzymes under appropriate buffer conditions^28^. Experiments were performed using 1 μl of 200 pmol oligo (50 μM in 4 μl reaction) or 2 μl (100 μM in 4 μl reaction) when 4 sites were tested (construct CircRep-8 m1–8 m1). Clones were checked by colony PCR (16 < n < 32) for statistical analysis and different patterns in the gel were digested and sequenced to confirm gene structure. Reagents Used and their Catalog number are provided in Supplementary Table 5.

### Statistical analysis

Statistical analysis was performed using one-way ANOVA followed by post-hoc Bonferroni or Holm corrections^41^. Letters indicate all pairs simultaneously compared. Values shown are inference based on method p-value.

## Additional Information

**How to cite this article**: Paoli, H. C. D. *et al*. An innovative platform for quick and flexible joining of assorted DNA fragments. *Sci. Rep.* **6**, 19278; doi: 10.1038/srep19278 (2016).

## Supplementary Material

Supplementary Information

Supplementary Information

## Figures and Tables

**Table 1:** Highlights between previous restriction enzyme-dependent methods and the TNT Cloning system.

Characteristics	Biobricks^14^	MoClo^31^	GB^18^	TNT-cloning
Require sequence overlap	***no***	*no*	*no*	*no*
Cloning rely on fragment restriction sites	*yes*	***no***	*no*	*no*
Take advantage of *type IIS* enzymes	*no*	***yes***	*yes*	*yes*
Support *endless assembly loop*	*no*	*no*	***yes***	***yes*** a
Fragments go from library to any assembling plasmids (*α*/*Ω*)	*n/a*	*n/a*	***yes***	*yes*
Signatures allow for both sense or anti-sense orientation of fragments	*no*	*no* b	*yes*	*yes*
Assembled fragments can be used directly or employed in new assemblies	***yes***	*no*	*yes*	*yes*
Cloning process leave scars (linkers/adaptors)	*yes*	*yes*	*yes*	***no***
Require domestication for enzymes	*yes*	*yes*	*yes*	***no***
Restrict type of fragments in library	*yes*	*yes*	*yes*	***no***
Require different signatures to clone library fragments	*n/a*	*yes*	*yes*	***no***
Require multiple PCRs for library construction	*yes*	*yes*	*yes*	***no***
Require multiple libraries to carry different fragment types	*n/a*	*yes*	*yes*	***no***
Provide a sharable platform for universal exchange of DNA segments	*no*	*no*	*no*	***yes***
Optimized buffer allow for quick and reliable one-pot digestion/ligation step	*no*	*no* c	*no* c	***yes***

Limitations firstly surpassed by each method are bold and italic.

^a^TNT-cloning loops up to three fragments at a time.

^b^A sub-library is necessary for inverting fragments.

^c^One-pot reactions are suggested but takes 1.5 h longer and are unsuited for sub-cloning efficient competent cells(10^7^ cfu/μg of puc19).

## References

[b1] Andrianantoandro E., Basu S., Karig D. K. & Weiss R. Synthetic biology: new engineering rules for an emerging discipline. Mol Syst Biol 2, 2006 0028 (2006). 10.1038/msb4100073 PMC1681505 16738572

[b2] Amit R., Garcia H. G., Phillips R. & Fraser S. E. Building enhancers from the ground up: a synthetic biology approach. Cell 146, 105–118 (2011). 21729783 10.1016/j.cell.2011.06.024 PMC3155781

[b3] Esvelt K. M. & Wang H. H. Genome-scale engineering for systems and synthetic biology. Mol Syst Biol 9, 641 (2013). 23340847 10.1038/msb.2012.66 PMC3564264

[b4] Gibson D. G. Programming biological operating systems: genome design, assembly and activation. Nat Methods 11, 521–526 (2014). 24781325 10.1038/nmeth.2894

[b5] McIsaac R. S. . Synthetic gene expression perturbation systems with rapid, tunable, single-gene specificity in yeast. Nucleic Acids Res 41, e57 (2013). 23275543 10.1093/nar/gks1313 PMC3575806

[b6] Litcofsky K. D., Afeyan R. B., Krom R. J., Khalil A. S. & Collins J. J. Iterative plug-and-play methodology for constructing and modifying synthetic gene networks. Nat Methods 9, 1077–1080 (2012). 23042452 10.1038/nmeth.2205 PMC3492501

[b7] Appleton E., Tao J., Haddock T. & Densmore D. Interactive assembly algorithms for molecular cloning. Nat Methods 11, 657–662 (2014). 24776633 10.1038/nmeth.2939

[b8] Schaerli Y. . A unified design space of synthetic stripe-forming networks. Nat Commun 5, 4905 (2014). 25247316 10.1038/ncomms5905 PMC4172969

[b9] Cameron D. E., Bashor C. J. & Collins J. J. A brief history of synthetic biology. Nat Rev Microbiol 12, 381–390 (2014). 24686414 10.1038/nrmicro3239

[b10] Smith M. T., Wilding K. M., Hunt J. M., Bennett A. M. & Bundy B. C. The emerging age of cell-free synthetic biology. FEBS Lett 588, 2755–2761 (2014). 24931378 10.1016/j.febslet.2014.05.062

[b11] Cheng A. A. & Lu T. K. Synthetic biology: an emerging engineering discipline. Annu Rev Biomed Eng 14, 155–178 (2012). 22577777 10.1146/annurev-bioeng-071811-150118

[b12] Gibson D. G. . Enzymatic assembly of DNA molecules up to several hundred kilobases. Nat Methods 6, 343–345 (2009). 19363495 10.1038/nmeth.1318

[b13] Walhout A. J. . Protein interaction mapping in *C. elegans* using proteins involved in vulval development. Science 287, 116–122 (2000). 10615043 10.1126/science.287.5450.116

[b14] Knight T. (DTIC Document, 2003).

[b15] Engler C., Kandzia R. & Marillonnet S. A one pot, one step, precision cloning method with high throughput capability. PLoS One 3, e3647 (2008). 18985154 10.1371/journal.pone.0003647 PMC2574415

[b16] DePaoli H. C., Borland A. M., Tuskan G. A., Cushman J. C. & Yang X. Synthetic biology as it relates to CAM photosynthesis: challenges and opportunities. J Exp Bot 65, 3381–3393 (2014). 24567493 10.1093/jxb/eru038

[b17] Guye P., Li Y., Wroblewska L., Duportet X. & Weiss R. Rapid, modular and reliable construction of complex mammalian gene circuits. Nucleic Acids Res 41, e156 (2013). 23847100 10.1093/nar/gkt605 PMC3763561

[b18] Sarrion-Perdigones A. . GoldenBraid 2.0: a comprehensive DNA assembly framework for plant synthetic biology. Plant Physiol 162, 1618–1631 (2013). 23669743 10.1104/pp.113.217661 PMC3707536

[b19] Patron N. J. . Standards for plant synthetic biology: a common syntax for exchange of DNA parts. New Phytol 208, 13–19 (2015). 26171760 10.1111/nph.13532

[b20] Hillson N. J., Rosengarten R. D. & Keasling J. D. j5 DNA assembly design automation software. ACS Synth Biol 1, 14–21 (2012). 23651006 10.1021/sb2000116

[b21] Roberts R. J., Vincze T., Posfai J. & Macelis D. REBASE-a database for DNA restriction and modification: enzymes, genes and genomes. Nucleic Acids Res 43, D298–299 (2015). 25378308 10.1093/nar/gku1046 PMC4383893

[b22] Boavida L. C., Qin P., Broz M., Becker J. D. & McCormick S. *Arabidopsis* tetraspanins are confined to discrete expression domains and cell types in reproductive tissues and form homo- and heterodimers when expressed in yeast. Plant Physiol 163, 696–712 (2013). 23946353 10.1104/pp.113.216598 PMC3793051

[b23] Slootweg E. . Nucleocytoplasmic distribution is required for activation of resistance by the potato NB-LRR receptor Rx1 and is balanced by its functional domains. Plant Cell 22, 4195–4215 (2010). 21177483 10.1105/tpc.110.077537 PMC3027179

[b24] Donnelly M. L. . Analysis of the aphthovirus 2A/2B polyprotein ‘cleavage’ mechanism indicates not a proteolytic reaction, but a novel translational effect: a putative ribosomal ‘skip’. J Gen Virol 82, 1013–1025 (2001). 11297676 10.1099/0022-1317-82-5-1013

[b25] Francois I. E. . Transgenic expression in *Arabidopsis* of a polyprotein construct leading to production of two different antimicrobial proteins. Plant Physiol 128, 1346–1358 (2002). 11950983 10.1104/pp.010794 PMC154262

[b26] Sawers R. J., Farmer P. R., Moffett P. & Brutnell T. P. *In planta* transient expression as a system for genetic and biochemical analyses of chlorophyll biosynthesis. Plant Methods 2, 15 (2006). 16953878 10.1186/1746-4811-2-15 PMC1578556

[b27] Praseuth D., Guieysse A. L. & Helene C. Triple helix formation and the antigene strategy for sequence-specific control of gene expression. Biochim Biophys Acta 1489, 181–206 (1999). 10807007 10.1016/s0167-4781(99)00149-9

[b28] Nikolova E. N., Goh G. B., Brooks C. L. 3rd & Al-Hashimi H. M. Characterizing the protonation state of cytosine in transient G.C Hoogsteen base pairs in duplex DNA. J Am Chem Soc 135, 6766–6769 (2013). 23506098 10.1021/ja400994e PMC3713198

[b29] Brunet E. . Intercalator conjugates of pyrimidine locked nucleic acid-modified triplex-forming oligonucleotides: improving DNA binding properties and reaching cellular activities. Nucleic Acids Res 33, 4223–4234 (2005). 16049028 10.1093/nar/gki726 PMC1181241

[b30] Ward B. Type IIS restriction enzyme footprinting I. Measurement of a triple helix dissociation constant with Eco57I at 25 degrees C. Nucleic Acids Res 24, 2435–2440 (1996). 8710518 10.1093/nar/24.12.2435 PMC145927

[b31] Weber E., Engler C., Gruetzner R., Werner S. & Marillonnet S. A modular cloning system for standardized assembly of multigene constructs. PLoS One 6, e16765 (2011). 21364738 10.1371/journal.pone.0016765 PMC3041749

[b32] Smanski M. J. . Functional optimization of gene clusters by combinatorial design and assembly. Nat Biotechnol 32, 1241–1249 (2014). 25419741 10.1038/nbt.3063

[b33] Chatterjee A., Kaznessis Y. N. & Hu W. S. Tweaking biological switches through a better understanding of bistability behavior. Curr Opin Biotechnol 19, 475–481 (2008). 18804166 10.1016/j.copbio.2008.08.010 PMC2766094

[b34] Kim J. H. . High cleavage efficiency of a 2A peptide derived from porcine teschovirus-1 in human cell lines, zebrafish and mice. PLoS One 6, e18556 (2011). 21602908 10.1371/journal.pone.0018556 PMC3084703

[b35] Varshavsky A. The N-end rule pathway and regulation by proteolysis. Protein Sci (2011). 10.1002/pro.666 PMC3189519 21633985

[b36] Galdzicki M. . The Synthetic Biology Open Language (SBOL) provides a community standard for communicating designs in synthetic biology. Nat Biotechnol 32, 545–550 (2014). 24911500 10.1038/nbt.2891

[b37] Canton B., Labno A. & Endy D. Refinement and standardization of synthetic biological parts and devices. Nat Biotechnol 26, 787–793 (2008). 18612302 10.1038/nbt1413

[b38] Sambrook J. & Russell D. W. (Cold Spring Harbor Laboratory Press, Cold Spring Harbor: , New York, , 2001).

[b39] Hajdukiewicz P., Svab Z. & Maliga P. The small, versatile pPZP family of *Agrobacterium* binary vectors for plant transformation. Plant Mol Biol 25, 989–994 (1994). 7919218 10.1007/BF00014672

[b40] Gao Y. & Zhao Y. Self-processing of ribozyme-flanked RNAs into guide RNAs *in vitro* and *in vivo* for CRISPR-mediated genome editing. J Integr Plant Biol 56, 343–349 (2014). 24373158 10.1111/jipb.12152

[b41] Aickin M. & Gensler H. Adjusting for multiple testing when reporting research results: the Bonferroni vs Holm methods. Am J Public Health 86, 726–728 (1996). 8629727 10.2105/ajph.86.5.726 PMC1380484

