# Supplementary material for: An innovative platform for quick and flexible joining of assorted DNA fragments
Source: Sci Rep. 2016 Jan 13;6:19278. doi: 10.1038/srep19278 (PMC4725820; doi:10.1038/srep19278)
Supplement: Supplementary Information [file srep19278-s1.pdf]

# Supplementary Figures and Tables

## "An innovative platform for quick and flexible joining of assorted DNA fragments"

Henrique Cestari De Paoli
Gerald A. Tuskan
Xiaohan Yang

Supplementary Figure 1

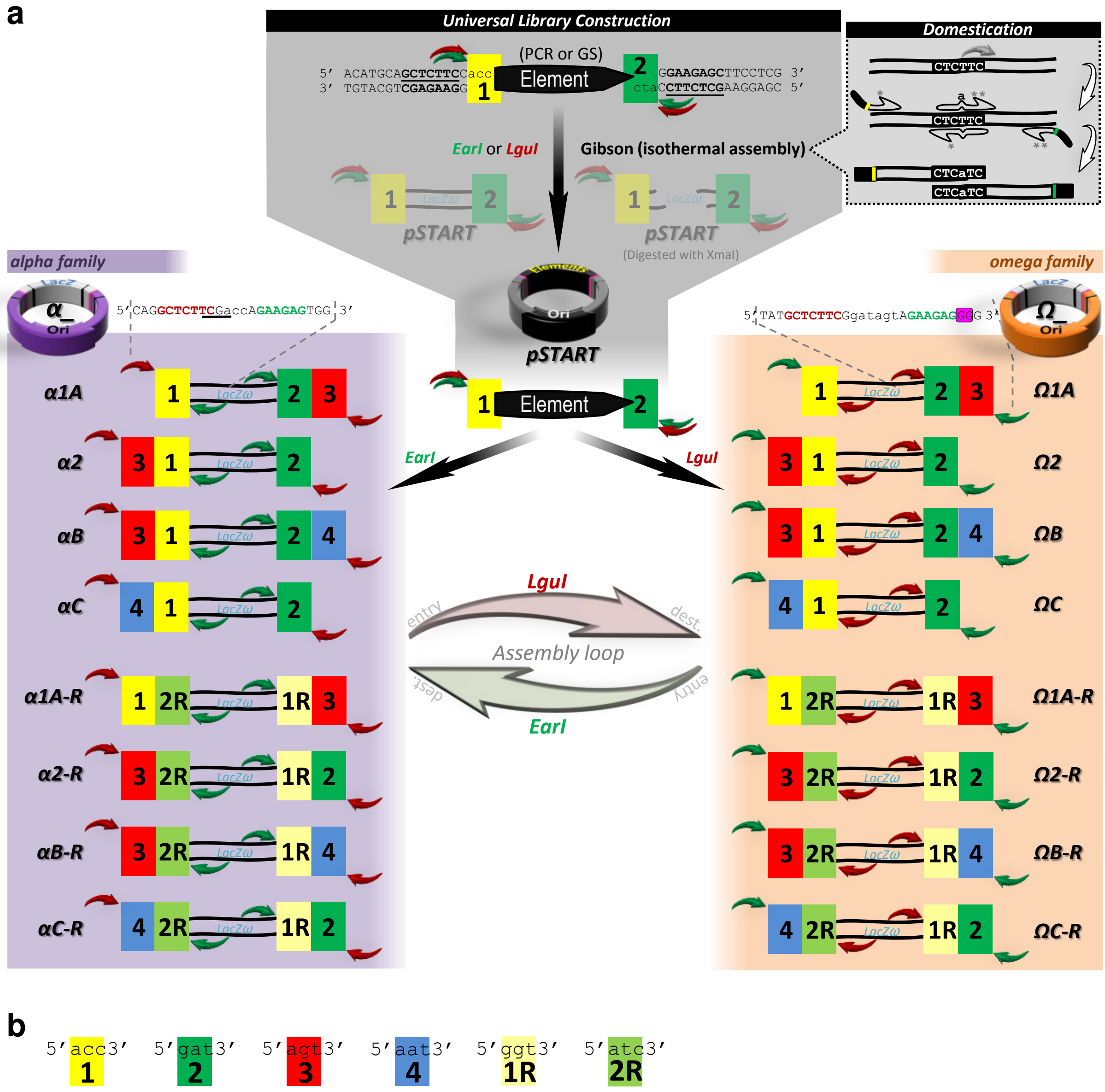


**Supplementary Figure 1 – TNT-cloning system technical details**

**(a)** Details of the library construction in the pSTART vector including the optional domestication process (top) along with the *α* family of vectors (purple, left) and *Ω* family of vectors (orange, right). Fragments of interest (element) can be produced by gene synthesis (“GS”) or amplified by PCR using the sequence shown (plus the three nucleotide code for signatures 1 and 2) to be inserted in the pSTART vector by either restriction enzymes (EarI/LguI) or “Gibson” isothermal assembly (which requires previous linearization of pSTART, as shown for XmaI). If extrusion of 5’CTCTTC3’ sites within the fragment is desired, overlapping oligos carrying a point mutation (e.g., T>A) can be used for amplification (asterisks denotes par of primers to be used) and directly used for Gibson assembly with the linearized pSTART

(Domestication, top right). pSTART carries signatures 1 (yellow) and 2 (green) used to transfer the fragment from the library to any member of either $\alpha$ family (using EarI, green arrow) or $\Omega$ family (using LguI, red arrow). Versions "R" in both families were created to allow fragment reorientation (sense or anti-sense insertion). Signatures 3 (red) and 4 (blue) will be used to join two (only signature 3) or three (both signatures 3 and 4) fragments together (Fig. 2b). Elements are also transferred from $\Omega$s to $\alpha s$ using EarI and from α*s* to $\Omega$s using LguI. The enzyme setup and the selection markers employed allow for one-pot reactions, which uses up to three plasmids carrying the "inserts" (entry) plus one destination (dest.) vector (either $\alpha$ or $\Omega$ member) and for multiple rounds of cloning alternating between $\alpha$ and $\Omega$ families (Assembly loop). If constructs are done in *E. coli* T7Express (NEB), the reporter LacZω allow for white/"blue" screening (using either "5-Bromo-4-chloro-3-indolyl β-D-galactopyranoside" or "5-Bromo-3-indolyl β-D-galactopyranoside" but not "8-Hydroxyquinoline-b-D-galactopyranoside", data not shown). Sequences at the top show the M.TaqI site (underlined, TCGA) in $\alpha$ members (absent in pSTART and $\Omega$ members). At the right side, the point mutation created upstream of EarI site to minimize LguI promiscuity is circled with pink background (see also Supplementary Fig. 7). Both, EarI and LguI sites match the arrow colors green and red, respectively. **(b)** Detailed sequence of each signature shown from 5' to 3'. Note that signatures 1R and 2R are the antisense of signatures 1 and 2, respectively. Signatures were chosen based on GC content, Adenine positioning to create M.TaqI site and to avoid stop codons as well as internal starting codons in case multiple CDS are to be joined.

Supplementary Figure 2

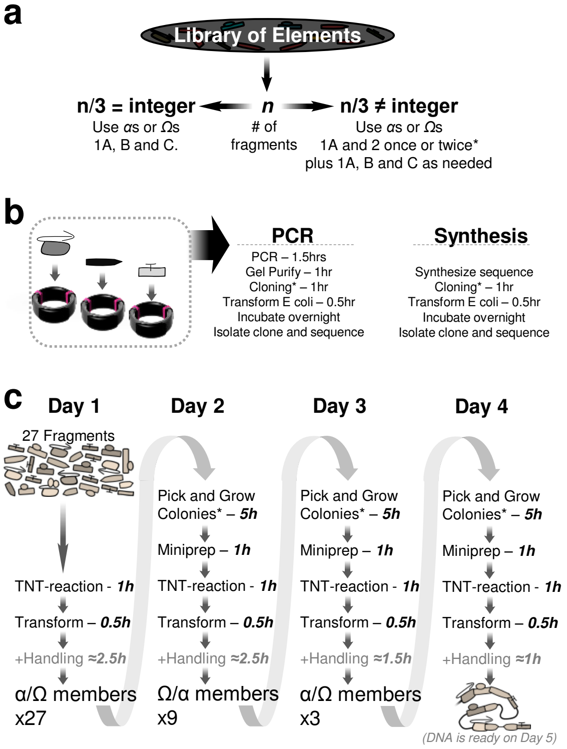


**Supplementary Figure 2 –Multi-gene assembling using the TNT-cloning system.**
**(a)** Ideogram: once cloned in the library, the number of fragments will define the vectors to be used and the number of cloning rounds necessary. If *n* number of fragments is divisible by 3 use only 1A, B and C versions. If *n-1* or *n-2* gives a number divisible by 3, versions 1A and 2 will be used twice or once, respectively, along with versions 1A, B and C as needed (asterisks). **(b)** Timeline with detailed description of each step during library construction into pSTART. Genes are either amplified or synthesized. If domestication is necessary, only one PCR is required upon use of Gibson isothermal assembly for cloning (asterisk; see also Supplementary Fig. 1). For multiple fragments assembly may be necessary to perform a regular TNT-reaction (asterisk, 50 cycles of '45sec 34 C, 4.5min 16 C'). **(c)** Timeline in days with detailed breakdown for hours necessary to accomplish each step outlined. Colonies can be picked, diluted in 10 µl of water, inoculated (5 µl) and simultaneously checked by colony PCR (1 µl), so, only positive clones are used for mini-prep and cloning (asterisk). TNT-reaction shown is for destination vectors linearized in advance (otherwise, a regular TNT-reaction of 4.5 h is necessary for binary/tertiary assembling). Growing and transformation times are shown for fast growing strains as Mach1$^{TM}$-T1$^{R}$ or T7Express. If non-domesticated fragments are present and the *BlindSpot* protocol is chosen, it has to be included separately (1-12 h). Constructs expected to be larger than 4 kb may require an extra hour for digestion and agarose gel analysis after mini-prep instead of colony PCR.

# Supplementary Figure 3

**a**

**TCAGATCCCGGGTCAATAGC**ATTCTCACCAATAAAAAACGCCCGGCGGCAACCGAGCGTTCTGAACAAATCCAGATGGAGTTCTGAGGTCATTACTGGAT
CTATCAACAGGAGTCCAAGCGGCCGCTTTTTTTACCTCCTAAAAGTTAAACAAAATTATTTCTAGAGGGAAACCGTTGTGGAATTGTGAGCGCTCACAAT
TCCACATATTATAATTGTTATCCGCTCACAAAGCAAATAAATTTTTCATGATTTCACTGTGCATGAAGCTCGTAATTGTTATCCGCTCACAATTaaacaa
Gcgctcatgagcccgaagtggcgagcccgatcttccccatcggtgatgtcggcgatataggcgccagcaaccgcacctgtggcgccggtgatgccggcca
cgatgcgtccggcgtagaggatcgagatctcgatcccgcgaaat**TAATACGACTCACTATAGG**GGAATTGTGAGCGGATAACAATTCCCCTCTAGAAATA
ATTTTGTTTAACTTTAAG***AAGGAG***ATATACC**ATGGGTCTGCCTCCGCTGCTGAGCCTGCCGAGCAATAGCGCACCGCGTAGCCTGGGTCGTGTTGAAACC**
**CCTCCGGAAGTTGTTGATTTTATGGTTAGCCTGGCCGAAGCACCGCGTGGTGGTCGTGTTCTGGAACCGGCATGTGCCCATGGTCCGTTTCTGCGTGCAT**
**TTCGTGAAGCACATGGCACCGGTTATCGTTTTGTTGGTGTTGAAATTGATCCGAAAGCACTGGATCTGCCACCGTGGGCAGAAGGTATTCTGGCAGATTT**
**TCTGCTGTGGGAACCGGGTGAAGCATTTGATCTGATTCTGGGTAATCCGCCTTATGGTATTGTTGGTGAAGCAAGCAAATATCCGATCCATGTTTTTAAA**
**GCCGTGAAAGATCTGTACAAAAAAGCCTTTAGCACCTGGAAAGGCAAATATAACCTGTATGGTGCCTTTCTGGAAAAAGCAGTTCGTCTGCTGAAACCGG**
**GTGGTGTTCTGGTTTTTGTTGTTCCGGCAACCTGGCTGGTGCTGGAAGATTTTGCACTGCTGCGTGAATTTCTGGCACGTGAAGGTAAAACCAGCGTTTA**
**TTATCTGGGTGAAGTTTTCCGCAGAAAAAAGTTAGCGCAGTGGTTATTCGTTTTCAGAAAAGCGGTAAAGGTCTGAGCCTGTGGGATACCCAAGAAAGC**
**GAAAGCGGTTTTACCCCGATTCTGTGGGCTGAATATCCGCATTGGGAAGGTGAAATTATTCGCTTTGAAACCGAAGAAACCCGCAAACTGGAAATTTCAG**
**GTATGCCGCTGGGTGACCTGTTTCATATCCGTTTTGCAGCACGTAGTCCGGAATTCAAAAAACATCCGGCAGTTCGTAAAGAACCGGGTCCGGGTCTGGT**
**TCCGGTTCTGACCGGTCGTAATCTGAAACCTGGTTGGGTTGATTATGAAAAAAATCATAGCGGTCTGTGGATGCCGAAAGAACGTGCAAAAGAACTGCGT**
**GATTTTTATGCAACACCGCATCTGGTTGTTGCACATACCAAAGGCACCCGTGTTGTTGCAGCATGGGATGAACGTGCATATCCGTGGCGTGAAGAATTTC**
**ATCTGCTGCCTAAAGAAGGTGTTCGTCTGGATCCGAGCAGCCTGGTTCAGTGGCTGAATAGCGAAGCAATGCAGAAACATGTTCGTACCCTGTATCGTGA**
**TTTTGTTCCGCATCTGACCCTGCGTATGCTGGAACGTCTGCCGGTTCGTCGTGAATATGGTTTTCATACCAGTCCGGAAAGCGCACGTAACTTTTAA**CAA
AGCCCGAAAGGAAGCTGAGTTGGCTGCTGCCACCGCTGAGCAATAACTAGCATAACCCCTTGGGGCCTCTAAACGGGTCTTGAGGGGTTTTTTGCTGAAA
GGAG**GCCACTCGAGCACCTAGGAG**

**b**

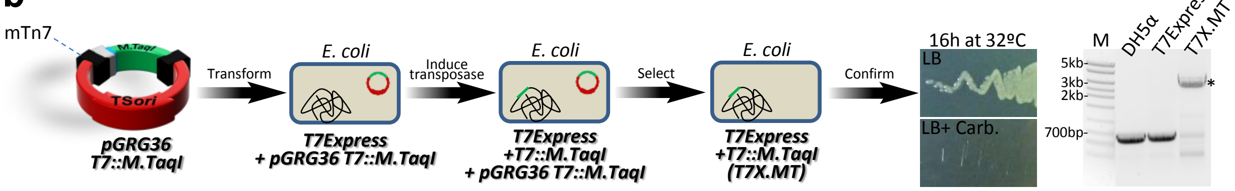


**Supplementary Figure 3 – M.TaqI gene cluster synthesized (1884 bp) and graphical representation of its insertion into the T7Express (NEB) genome.**

**(a)** M.TaqI gene was codon optimized for *E. coli* as shown (green highlight). T7 elements, promoter (light blue highlight) and terminator (dark blue highlight) plus the *lac*Operator (yellow highlight) and the overlap region for cloning in pGRG36 (dark red) at the NotI site (underlined) are shown. Ribosome biding site is bold/italic (AAGGAG), light gray is a secondary expression cassette and lower case is a linker sequence. Cloning region is flanked by the terminal repeats of Tn7 (mTn7) at the pGRG36 vector [36]. **(b)** Graphical representation for construction of T7X.MT as described in McKenzie and Craig [36]. Briefly, the synthetic fragment shown in "**a**" was cloned in pGRG36 using the Gibson assembly strategy, transformed in the T7Express (NEB) strain and selected in carbenicillin plates at 30-32°C due to a thermosensitive replication origin (TS*ori*). Five single colonies were picked and inoculated in liquid LB media in the presence of 0.1% L-arabinose to induce the transposases overnight. Cultures were diluted and plated at 42°C overnight (to block plasmid replication and allow growth of transgenes). Single colonies were again grown in liquid media and re-streaked in LB plates. Carbenicillin sensitivity tests were conducted (16 h growth at 32°C) and one sensitive strain (defined as T7X.MT) had the insertion into the genome confirmed by PCR. In the absence of a Tn7 insertion, the genomic primers will yield a ≈678 bp product (DH5α and T7Express, controls). In the presence of the Tn7insertion this product will

increase and final size should be larger than 2562 bp but smaller than 3466 bp (asterisk), depending on the strain used for engineering. The strain shown was used for our methylation tests (see Fig. 3) and hosts the TNT-system vectors. We choose the T7X strain because of its specific genotype that allows stable expression of exogenous proteins (deficient in proteases *Lon* and *OmpT*) and its ability in not restricting methylated DNA (*McrA*$^-$, *McrBC*$^-$, *EcoBr*$^-$*m*$^-$, *Mrr*$^-$). We found the M.TaqI expression significantly impairs *E. coli* growth if incubation temperature goes over 42°C or IPTG concentration goes over 0.5 mM (data not shown).

## Supplementary Figure 4

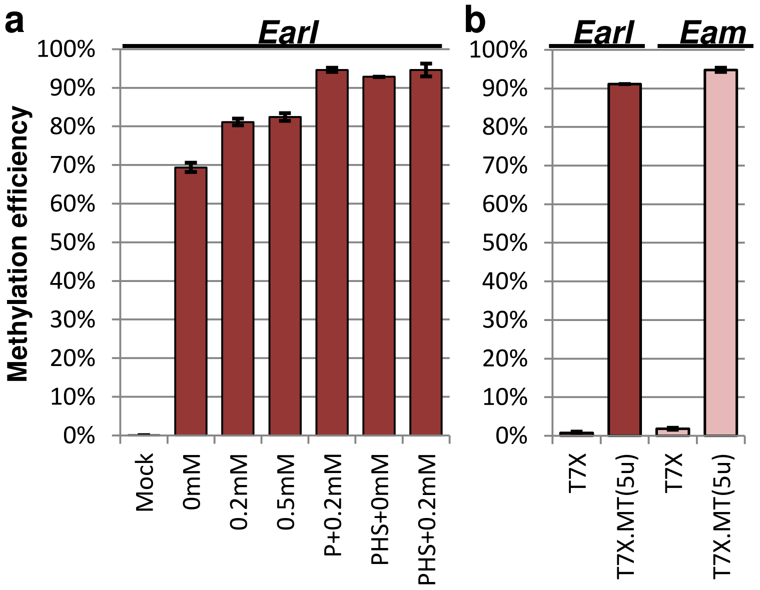


**Supplementary Figure 4 – Methylation efficiency of T7X.MT at different conditions.**

*E. coli* strains T7Express (mock) or T7X.MT were transformed with the M.Test vector, plated and two colonies were picked for overnight grown (≈18 h) in liquid LB media. Digested vector had the agarose gel bands quantified and expressed as percentage: 1-[digested/(digested+linearized)] in each tube. **(a)** Conditions shown are: IPTG concentration during the liquid growth (0 mM, 0.2 mM or 0.5 mM); presence (0.3 mM) or absence of IPTG in the plate during transformation with M.Test plasmid (*P*); presence or absence of a heat shock treatment for 1 h at 50°C right before DNA extraction (*HS*). **(b)** Stability of methylated DNA after 11 weeks subjected to both EarI (5 U) and Eam1104I (5 U) digestion. New replicates of P+0.2 mM were used (n=3). Note the minimal difference between freshly prepared DNA (shown in **A**) and 11-weeks-old DNA (5.9% SE±1.0) to EarI inhibition and the similar inhibition of Eam1104I to the same DNA.

## Supplementary Figure 5

| | Sites | Type IIS / Methylase | BspQI | LguI | SapI | EarI |
|---|---|---|---|---|---|---|
| Expected | GAGCTCTTC<br>CTCGAGAAG | M.SacI | ? | ? | ? | ? |
| | tGCTCTTCG<br>aCGAGAAGC | M.SssI | 100%* | 100% | 100% | 50% |
| | GCTCTTCGA<br>CGAGAAGCT | M.TaqI | 100%* | ? | 0%* | 0% |

| | Sites | Type IIS / Methylase | BspQI | LguI | SapI | EarI |
|---|---|---|---|---|---|---|
| Observed | GAGCTCTTC<br>CTCGAGAAG | M.SacI | ≈20% | ≈35% | ≈15% | ≈20% |
| | tGCTCTTCG<br>aCGAGAAGC | M.SssI (I) | ≈100% | ≈100% | ≈60% | ≈75% |
| | CGCTCTTCc<br>GCGAGAAGg | M.SssI (II) | ≈100% | ≈100% | ≈30% | ≈100% |
| | GCTCTTCGA<br>CGAGAAGCT | M.TaqI | ≈90% | <0.1% | <0.1% | <0.1% |

**Supplementary Figure 5 – Methylation sensitivity chart for BspQI, LguI, SapI and EarI enzymes.**

*Top*- Sensitivity chart extracted from REBASE [21]. Asterisks represent testing using synthetic oligonucleotides and question mark represents unavailable data. *Bottom*- Sensitivity observed on our assays. In our hands, M.SssI partially inhibited SapI activity. At least two replicates were performed for each pair of enzyme-methylase. Evaluation was performed using the same template used for EarI assays (see Fig. 3). Digestion ran for 1hour at 37°C (except for BspQI, where 50°C were used) using 5 U of each enzyme (except SapI, where 10 U were used) in 20 µl reaction volume. Amount of DNA was around 400 ng (PCR product) and 1 µg (plasmid DNA). Overlapped sites between methylase biding site and the *type IIS* enzymes are highlighted in black/bold. Nucleotides modified are indicated by a $CH_3$ branch.

## Supplementary Figure 6

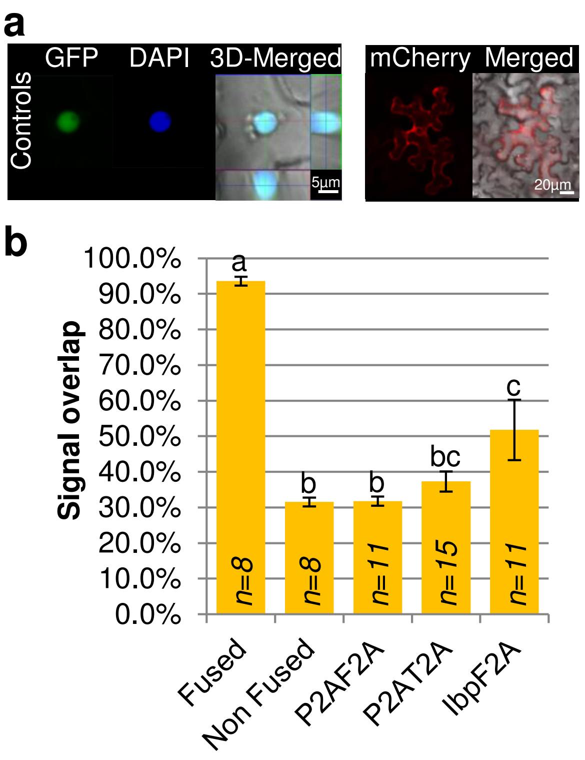


**Supplementary Figure 6 – Confocal image of controls and assessment of enhanced split efficiency of P2A clusters.**

(**a**) Representative confocal images of *35S::NLS-GFP-NLS-Term* (GFP control) localized in the nucleus and *35S::tag-PmCherry-Term* (PmCherry control) localized in the karyoteca and plasma membrane. Signals were captured at a reduced window spectrum for GFP (493-556 nm) and mCherry (578-650 nm). 3D-merge shows 12 stacked images over 6 µm. For 4',6-diamidino-2-phenylindole (DAPI) images, leaf tissue (0.5 cm2) were fixed in formalin+0.2% Triton-X for 30 min and stained (1.25 µg/ml) for 15 min right before analysis, washed in PBS and mounted in water. Scale bars are indicated for each panel. Plants were grown on standard greenhouse conditions and infiltration was performed as described previously[36]. **(b)** At least one hundred cells from confocal images were visually scored for mCherry and GFP fluorescence separation in each P2A cluster constructs and 8-15 images taken had each channel plotted through a 10-18µm section of a nucleus (Fig. 4c). Graph area for each channel were measured using ImageJ and the raw signal overlap [(PmCherry/GFP)*100] is shown. Split efficiency was then calculated related to the Non Fused control: *P2AF2A* (99.7% SE±1.2, n=11) and *P2AT2A* (94.2% SE±2.8, n=15) showed the best sub-cellular separation of signals, against the partial separation observed for *IbpF2A* (79.7% SE±8.4, n=11). Holm analysis, $p<0.01$ (a-b) and $p<0.05$ (b-c).

Supplementary Figure 7

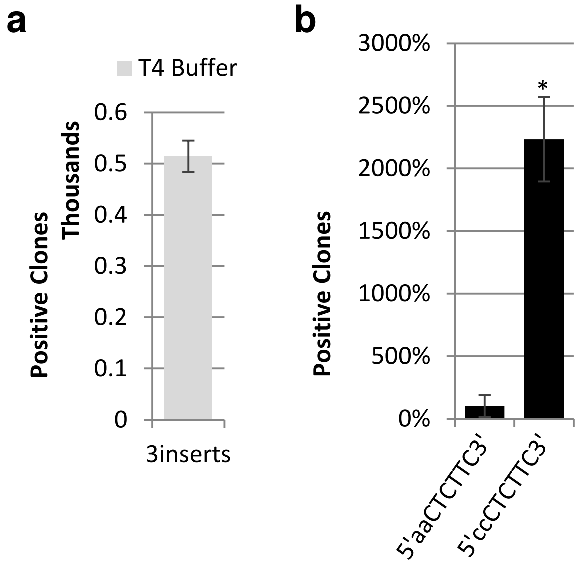


**Supplementary Figure 7 – Cloning efficiency of T4DNA Ligase buffer for 3 elements cloning and two different versions of Ω vector with upstream point mutations.**

**(a)** Three fragments assembly from both ways reactions (*α*'s to *Ω*'s and vice-versa) using T4 DNA ligase buffer (T4 Buffer) using the GoldenBraid (50 cycles of: 37°C 2min, 16°C 5min) reaction. Error bars are from 3 independent cloning reactions. Number of clones shown are positive clones confirmed by colony PCR ($16<n<32$). Same lot of competent cells was used here and for Fig. 4e. **(b)** Optimized nucleotide sequence upstream of EarI site to minimize unspecific cleavage by LguI. Most clones obtained from tertiary assembly reactions were unspecific transfer of one fragment from *α1A* to *Ω1A*, which represents unspecific cleavage of LguI at the EarI site exposing the signature number 3 of *Ω1A* rather than signature number 2 for proper cloning of the final tertiary construct (data not shown; see also Supplementary Fig. 1). Therefore, three new point mutations were tested at the 5' end of EarI site. The best result is shown here, for sequence 5'ccCTCTTC3' where more than 2000% increase in cloning efficiency were observed compared to the previous *Ω* version 5'aaCTCTTC3'. This sequence were implemented in all *Ω* members at sites that bear 2 signatures side by side and represent the final set of *Ω* plasmids (Supplementary Fig. 1). Error bars are standard error for 4 biological replicates. Bonferroni analysis are $p<0.05$(*) and $p<0.01$(**; letters).

# Supplementary Figure 8

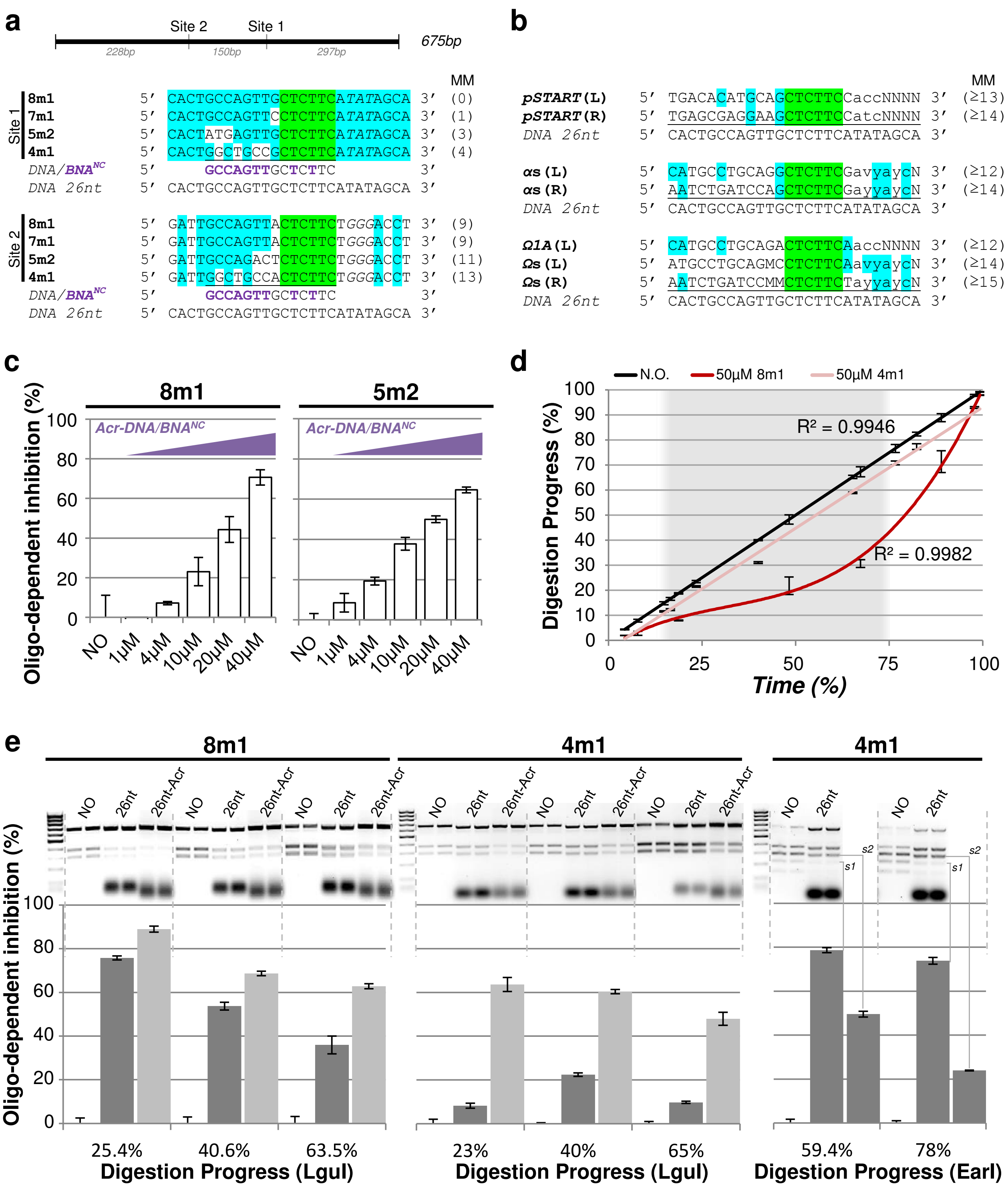


**Supplementary Figure 8 – Oligo-dependent inhibition of digestion at 5'(G)CTCTTC3' sites.**

**(a)** DNA templates studied with nomenclature, sequence and number of mismatches (MM) related to the 14nt DNA/BNA$^{NC}$ (purple) and 26-nt DNA oligonucleotides. PCR product (675 bp) was used here.

Restriction sites for LguI and EarI as well as the distance between them are indicated at sites 1 and 2. 5'CTCTTC3' sequence and matches to the oligo are indicated in green and blue, respectively. **(b)** TNT-plasmids showing the 5'CTCTTC3' flanking sequences aligned to the 26-nt DNA oligo. All the $\alpha$ and $\Omega$ members (except $\Omega$1A) share the same sequence within each family at the left (L) or at the right (R) side of the LacZω reporter, respectively. Number of mismatches is also indicated. V = A, C or G, M = A or C, Y = C or T, N = any. **(c)** Oligo-dependent inhibition of digestion at site 1 using the templates 8m1 (200 ng) and 5m2 (200 ng) during increasing amounts of 14-nt DNA/BNA$^{NC}$ linked to acridine[28] (Acr-DNA/BNA$^{NC}$). Oligos were incubated for 6 h at 37°C in Tris-Acetate buffer (50 mM pH 7.0, 20 mM NaCl, 10 mM $MgCl_2$) in 5 µl before being subjected to digestion (1 U LguI, 25 min at 34°C) in 10 µl final volume. Reaction was stopped and gel bands quantified and plotted as percentage of the control without oligo (N.O.). Digestion progress is the ratio of digested/(digested+non-digested) bands in each tube. Values when present are expressed as percentage of N.O. tube. Digestion progress in these panels was 19.7% (SE±2.2). Oligo amount is indicated. **(d)** Digestion progression curve in the absence (N.O.) or presence of the 26-nt DNA oligo (50 µM) in two different templates: 8m1 (0 mismatch, 180 ng) and 4m1 (4 mismatches, 180 ng). Oligo and templates were incubated for 45°C-12°C (every 3°C, 1 h each) in 4 µl Tris-HCl buffer (50 mM pH 5.8, 75 mM NaCl, 10 mM $MgCl_2$, 2 mM DTT) before being subjected to digestion (1.5 U LguI, 2-30 min at 25°C) in 10 µl final volume (completed with Tris-HCl 50 mM pH 6.3, 10 mM $MgCl_2$, 2mM DTT); 5, 10 and 15 min in these conditions corresponds to 24.4% (SE±0.6), 40.3% (SE±0.6) and 62.0% (SE±2.1) digestion progression, respectively. Digestion is fairly linear in the N.O. tube in the range of 2 min (14.8% SE±0.6) to 20 min (76.6% SE±1.6) ($R^2$=0.9967), gray background. $R^2$ values shown are for linear (4m1, 0.9946) and polynomial (8m1, 0.9982) trends. **(e)** Oligo-dependent inhibition of digestion at sites 1 (LguI and EarI) and 2 (EarI only) using the templates 8m1 (180ng) and 4m1 (180ng). The DNA oligos 26 nt and 26 nt fused to acridine (26nt-Acr) (always 50 µM) were incubated with each template as in **d**. Reaction was stopped and gel bands (shown) quantified and plotted as percentage of the control without oligo (N.O.) as in **c**. EarI activity on each site, s1 (4 mismatches) and s2 (13 mismatches), was evaluated independently (right panel). The 4m1, 5m2, 7m1 and 8m1 templates were originally cloned in pSTART from three GBlocks: two universal GBlocks (TFOsynt_Uni5' and TFOsynt_Uni3') along with its respective central fragment TFOsynt_4m1, TFOsynt_5m2, TFOsynt_7m1, TFOsynt_8m1 (Supplementary Table 3).

**Supplementary Table 1** - Type IIS enzymes available on Rebase (Roberts et al., 2015, Nuc. Acid. Res.)

| *Enzymes* | *Recognition Sequence* | *Suppliers* | *Reach* | *Reach* | *Extension** | *Recognition Sequence* |
|---|---|---|---|---|---|---|
| Bco5I | CTCTTC (1/4) | - | 1 | 4 | 3 nt 5' | 5' CTCTTCN^ |
| Bco116I | CTCTTC (1/4) | - | 1 | 4 | 3 nt 5' | 5' CTCTTCN^ |
| BcoKI | CTCTTC (1/4) | - | 1 | 4 | 3 nt 5' | 5' CTCTTCN^ |
| BsaXI | (9/12) ACNNNNNCTCC (10/7) | N | 10 | 7 | 3 nt 3' | 5' ACNNNNNCTCCNNNNNNNNNN^ |
| BsaXI | GGAGNNNNNGT (10/7) | N | 12 | 9 | 3 nt 3' | 5' GGAGNNNNNGTNNNNNNNNNNNN^ |
| BseZI | CTCTTC (1/4) | - | 1 | 4 | 3 nt 5' | 5' CTCTTCN^ |
| BspQI | GCTCTTC (1/4) | N | 1 | 4 | 3 nt 5' | 5' GCTCTTCN^ |
| BssIMI | GGGTC (-3/0) | - | -3 | 0 | 3 nt 5' | 5' GG^GTC |
| Bst6I | CTCTTC (1/4) | IV | 1 | 4 | 3 nt 5' | 5' CTCTTCN^ |
| Bsu6I | CTCTTC (1/4) | - | 1 | 4 | 3 nt 5' | 5' CTCTTCN^ |
| CatHI | CTCTTC (1/4) | - | 1 | 4 | 3 nt 5' | 5' CTCTTCN^ |
| Eam1104I | CTCTTC (1/4) | B | 1 | 4 | 3 nt 5' | 5' CTCTTCN^ |
| EarI | CTCTTC (1/4) | N | 1 | 4 | 3 nt 5' | 5' CTCTTCN^ |
| Ksp632I | CTCTTC (1/4) | - | 1 | 4 | 3 nt 5' | 5' CTCTTCN^ |
| LguI | GCTCTTC (1/4) | B | 1 | 4 | 3 nt 5' | 5' GCTCTTCN^ |
| PciSI | GCTCTTC (1/4) | I | 1 | 4 | 3 nt 5' | 5' GCTCTTCN^ |
| RleAI | CCCACA (12/9) | - | 12 | 9 | 3 nt 3' | 5' CCCACANNNNNNNNNNNN^ |
| SapI | GCTCTTC (1/4) | N | 1 | 4 | 3 nt 5' | 5' GCTCTTCN^ |
| SimI | GGGTC (-3/0) | - | -3 | 0 | 3 nt 5' | 5' GG^GTC |
| VpaK32I | GCTCTTC (1/4) | - | 1 | 4 | 3 nt 5' | 5' GCTCTTCN^ |

*Filter was applied to exhibit " 3 nt 3' " and " 3 nt 5' ".

**Supplementary Table 2**. List of fragments relevant for this work and their respective vectors.

| *Vector* | *Inserts* |
|---|---|
| pSTART | d35S_h-h, PmCherry, Lumio, RGR gene, P2A, T2A, Cas9*, F2A, Ibp, GFP, 35SProm, 35STerm, NLS, NosProm, rGUS, HCC (Hig-CodA, see methods), Kan-ORF, 8m1*, 7m1*, 5m2*, 4m1*, 8m2*, CircRep |
| α1A | NosProm, d35S_h-h, NLS, 35SProm, RGR gene, GFP cluster, 35SProm-L-PmCherry, CircRep-8m1-8m1* |
| α1AR | GUS, HCC cluster |
| α2 | 35STerm, GFP, RGR gene, nGFP-L-Term |
| α2R | 35SProm, Kan-ORF |
| αB | Kan-ORF, 35STerm, GFP, Lumio, P2A-T2A, P2A-F2A, Lumio, RGR gene**, Ibp-F2A |
| αBR | GFP, 8m2* |
| αC | 35STerm, P2A-F2A, NLS, PmCherry, RGR gene, nGFP-L-Term, nGFP-L-Term, 8m2* |
| αCR | 35SProm |
| Ω1A | PmCherry cluster, P2A, Ibp, P2A-T2A cluster, nGFP, 35SProm, 35SProm-L-PmCherry, PmCherry cluster, 8m1*, 7m1*, CircRep |
| Ω1AR | 35STerm |
| Ω2 | 35SProm-GUS, T2A, F2A |
| ΩB | P2A-F2A cluster, nGFP, Lumio, 8m1* |
| ΩC | Ibp-F2A cluster, PmCherry, 35STerm, 8m1* |
| ΩCR | 35SProm |

*Fragments were either partially or not domesticated.

**RGR gene, ribozyme-gRNA-ribozyme[40]

**Supplementary Table 3.** Primers (green), Gblocks (blue) and GeneSynthesis (purple) used in this study and their respective name, length(bp) and sequence.

| *Name* | *Length* | *Sequence* |
|---|---|---|
| pUPD-FW1 | 32 | GCTCTTCCACCCCGGGGCTGGCTTAACTATGC |
| pUPD-RW1 | 27 | TGGCGTAATAGCGAGGAGGCCCGCACC |
| pUPD-FW2 | 27 | GGTGCGGGCCTCCTCGCTATTACGCCA |
| pUPD-RW2 | 31 | CTCTAGAGGATCCCCTGGTACCGAGCTCGAA |
| pUPD-FW3 | 31 | TTCGAGCTCGGTACCAGGGGATCCTCTAGAG |
| pUPD-RW3 | 35 | GCTCTTCCATCCCGGGCGCCCAATACGCAAACCGC |
| pUPD-FW4 | 34 | CCCGGGATGGAAGAGCTTCCTCGCTCACTGACTC |
| pUPD-RW4 | 41 | GCTTCAATAATATTGAAAAAGGAGGAGTATGAGTATTCAAC |
| pUPD-FW5 | 41 | GTTGAATACTCATACTCCTCCTTTTTCAATATTATTGAAGC |
| pUPD-RW5 | 33 | GCCCCGGGGTGGAAGAGCTGCATGTGTCAGAGG |
| pUPD-RW3.1 | 27 | CAGCTATGACCATGATTACGGATTCAC |
| FW_adap | 67 | CTCTTCCACCCCGGGGCTGGCTTAACTATGCGGCATCAGAGCTTATTTTTGACACCAGACCAACTGG |
| pUPD-FW3.1 | 31 | CGTAATCATGGTCATAGCTGTTTCCTGTGTG |
| pUPD-RW5 | 33 | GCCCCGGGGTGGAAGAGCTGCATGTGTCAGAGG |
| KStrat2_TNT-Fw | 26 | CATTACAGCTTACGAACCGAACGAGG |
| KStrat2_TNT-Rw | 21 | GCAGCGAGTCAGTGAGCGAGG |
| Kan_to_O-FW2 | 25 | GGAATTTATGCCGCTTCCGACCATC |
| KStrat2_TOP-Rw | 21 | CCTCGCTCACTGACTCGCTGC |
| KStrat2_TOP-FW | 37 | TCGGTTCGTAAGCTGTAATGTTCCTGGCAGCTCTGGC |
| Kan_to_O-RW1 | 25 | GATGGTCGGAAGCGGCATAAATTCC |
| αΩVector_FW | 24 | TGGATCAGATTGTCGTTTCCCGCC |
| αΩVector_RW | 24 | CTGCAGGCATGCAGCTCGAATTAT |
| aO_vector-nested-RW | 27 | GCTCGAATTATCGATCATGAGCGGAGA |
| aO_vector-nested-FW | 30 | GTTTCCCGCCTTCAGTTTAAACTATCAGTG |
| PCR2_to_αVector-Fw | 24 | GCTGCATGCCTGCAGGCTCTTCGA |
| PCR2_to_αVector-Rw | 38 | GGG AAA CGA CAA TCT GATCCA GCT CTT CGA |
| α1A-Fw | 55 | GCAGGCTCTTCGACCAGAAGAGT GGC TTA ACT ATG CGG CAT CAG AGC |
| α1A-Rw | 63 | GATCCA GCT CTT CGA CTA TCT GAA GAG T ATA CGC AAA CCG CCT CTC CC |
| α2-Fw | 58 | GCAGGCTCTTCGAGTACCAGAAGAGT GGC TTA ACT ATG CGG CAT CAG AGC |
| α2-Rw | 58 | GATCCAGCT CTT CGA TCT GAA GAG T ATA CGC AAA CCG CCT CTC CC |
| αB-Fw | 58 | GCAGGCTCTTCGAGTACCAGAAGAGT GGC TTA ACT ATG CGG CAT CAG AGC |
| αB-Rw | 62 | GATCCAGCT CTT CGA TTA TCT GAA GAG T ATA CGC AAA CCG CCT CTC CC |
| αC-Fw | 58 | GCAGGCTCTTCGAATACCAGAAGAGT GGC TTA ACT ATG CGG CAT CAG AGC |
| αC-Rw | 58 | GATCCAGCT CTT CGA TCT GAA GAG T ATA CGC AAA CCG CCT CTC CC |
| α1R-Fw | 58 | GCAGGCTCTTCGACCATCAGAAGAGT GGC TTA ACT ATG CGG CAT CAG AGC |
| α1R-Rw | 62 | GATCCAGCT CTT CGA CTA CCT GAA GAG T ATA CGC AAA CCG CCT CTC CC |
| α2R-Fw | 58 | GCAGGCTCTTCGAGTATCAGAAGAGT GGC TTA ACT ATG CGG CAT CAG AGC |
| α2R-Rw | 62 | GATCCAGCT CTT CGA TCA CCT GAA GAG T ATA CGC AAA CCG CCT CTC CC |
| PCR2_to_ΩVector-Fw | 22 | GCTGCATGCCTGCAGACTCTTC |
| PCR2_to_ΩVector-Rw | 34 | GGG AAA CGA CAA TCT GATCCA ACTCTTC |
| Ω1A-Fw | 56 | GCAG ACTCTTCAACCCGAAGAGC GGC TTA ACT ATG CGG CAT CAG AGC |
| Ω1A-Rw | 63 | GATCCA ACT CTT CTA CTA TCC GAA GAG C ATA CGC AAA CCG CCT CTC CC |
| Ω2-Fw | 59 | GCAG ACTCTTCAAGTACCCGAAGAGC GGC TTA ACT ATG CGG CAT CAG AGC |
| Ω2-Rw | 59 | GATCCA ACT CTT CTA TCC GAA GAG C ATA CGC AAA CCG CCT CTC CC |
| ΩB-Fw | 59 | GCAG ACTCTTCAAGTACCCGAAGAGC GGC TTA ACT ATG CGG CAT CAG AGC |

| | | |
|---|---|---|
| ΩB-Rw | 63 | GATCCA ACT CTT CTA TTA TCC GAA GAG C ATA CGC AAA CCG CCT CTC CC |
| ΩC-Fw | 59 | GCAG ACTCTTCAAATACCCGAAGAGC GGC TTA ACT ATG CGG CAT CAG AGC |
| ΩC-Rw | 59 | GATCCA ACT CTT CTA TCC GAA GAG C ATA CGC AAA CCG CCT CTC CC |
| Ω1R-Fw | 59 | GCAG ACTCTTCAACCATCCGAAGAGC GGC TTA ACT ATG CGG CAT CAG AGC |
| Ω1R-Rw | 63 | GATCCA ACT CTT CTA CTA CCC GAA GAG C ATA CGC AAA CCG CCT CTC CC |
| Ω2R-Fw | 59 | GCAG ACTCTTCAAGTATCCGAAGAGC GGC TTA ACT ATG CGG CAT CAG AGC |
| Ω2R-Rw | 63 | GATCCA ACT CTT CTA TCA CCC GAA GAG C ATA CGC AAA CCG CCT CTC CC |
| EarI-FW1 | 31 | GTTTTTCATTACCGACGAGATCGAGGCGGAG |
| EarI-FW2 | 31 | GCGCACAGCCGACGAGCTGCAAAAAG |
| EarI-FW3 | 31 | CAGGCGCTCTTACGCTTCCTCGCTC |
| EarI-FW4 | 31 | TTCGCCGCCAAGTTCTTCAGCAATATC |
| EarI-FW5 | 31 | CGAGCCCCTGATGTTCTTCGTCCAG |
| EarI-RW1 | 31 | CTCCGCCTCGATCTCGTCGGTAATGAAAAAC |
| EarI-RW2 | 31 | CTTTTTGCAGCTCGTCGGCTGTGCGC |
| EarI-RW3 | 31 | GAGCGAGGAAGCGTAAGAGCGCCTG |
| EarI-RW4 | 31 | GATATTGCTGAAGAACTTGGCGGCGAA |
| EarI-RW5 | 31 | CTGGACGAAGAACATCAGGGGCTCG |
| TNT-αΩ-seqRW | 22 | CTCTTAGGTTTACCCGCCAATA |
| TNT-αΩ-seqFW | 22 | AACGTGACTCCCTTAATTCTCC |
| pUPD_adap_met.test-FW | 43 | CTCTTCGACCCCGGGGC TGGCTTAACTATGCGGCATCAGAGC |
| pUPD-RW5-M_Test | 33 | GCCCCGGGGTCGAAGAGCTGCATGTGTCAGAGG |
| pUPD-seqFW | 20 | GCCACCTGACGTCTAAGAAA |
| pUPD-seqRW | 25 | CCTGATTCTGTGGATAACCGTATTA |
| TNT-Lumio_FW | 72 | ACATGCAGCTCTTCCACCGGTGCTGGTGGTTGCTGCCCTGGTTGCTGCGGTGGTGATGGAAGAGCTTCCTCG |
| TNT-Lumio_RW | 72 | CGAGGAAGCTCTTCCATCACCACCGCAGCAACCAGGGCAGCAACCACCAGCACCGGTGGAAGAGCTGCATGT |
| TNT-GFP_FW | 35 | ACATGCAGCTCTTCCACCGTGAGCAAGGGCGAGGA |
| TNT-GFP_RW | 38 | CGAGGAAGCTCTTCCATCCTTGTACAGCTCGTCCATGC |
| TNT-NLS_FW | 66 | ACATGCAGCTCTTCCACCCCTAAGAAGAAGCGTAAGGTCGAGGACCCTGATGGAAGAGCTTCCTCG |
| TNT-NLS_RW | 66 | CGAGGAAGCTCTTCCATCAGGGTCCTCGACCTTACGCTTCTTCTTAGGGGTGGAAGAGCTGCATGT |
| TNT-P2A_FW | 93 | ACATGCAGCTCTTCCACCGCTACCAACTTCTCTCTCCTCAAGCAGGCTGGTGACGTCGAGGAGAACCCTGGTCCTGATGGAA GAGCTTCCTCG |
| TNT-P2A_RW | 93 | CGAGGAAGCTCTTCCATCAGGACCAGGGTTCTCCTCGACGTCACCAGCCTGCTTGAGGAGAGAGAAGTTGGTAGCGGTGG AAGAGCTGCATGT |
| TNT-T2A_FW | 96 | ACATGCAGCTCTTCCACCCGTGCTGAGGGTCGTGGTTCTCTCCTCACCTGCGGTGACGTCGAGGAGAACCCTGGTCCTGAT GGAAGAGCTTCCTCG |
| TNT-T2A_RW | 96 | CGAGGAAGCTCTTCCATCAGGACCAGGGTTCTCCTCGACGTCACCGCAGGTGAGGAGAGAACCACGACCCTCAGCACGGG TGGAAGAGCTGCATGT |
| TNT-IbP_FW | 90 | ACATGCAGCTCTTCCACCCCTTGCTCTAACGCTGCTGACGAGGTCGCTACCCCTGAGGACGTCGAGCCTGGTGATGGAAGA GCTTCCTCG |
| TNT-IbP_RW | 90 | CGAGGAAGCTCTTCCATCACCAGGCTCGACGTCCTCAGGGGTAGCGACCTCGTCAGCAGCGTTAGAGCAAGGGGTGGAAG AGCTGCATGT |
| TNT-PmCherry_FW1 | 37 | ACATGCAGCTCTTCCACCATGGCAAAGGATGTGGAAG |
| TNT-PmCherry_RW1 | 31 | GATGTATAAGAATAGGAGAGTGGCTACGAAC |
| TNT-PmCherry_FW2 | 31 | GTTCGTAGCCACTCTCCTATTCTTATACATC |
| TNT-PmCherry_RW2 | 37 | CGAGGAAGCTCTTCCATCAGATCTGTACAGCTCGTCC |
| TNT-35SPro_FW | 40 | ACATGCAGCTCTTCCACCCACAACATACGAGCCGGAAGCA |
| TNT-35SPro_RW | 42 | CGAGGAAGCTCTTCCATCCATGGCTATCGTTCGTAAATGGTG |
| TNT-35STerm_FW | 45 | ACATGCAGCTCTTCCACCTAAGTAGCTGAATCCCGCGGCCATGCT |

| | | |
|---|---|---|
| TNT-35STerm_RW | 37 | CGAGGAAGCTCTTCCATCTCGGGCTAGGCCCGACGTC |
| TNT-F2A_FW | 156 | ACATGCAGCTCTTCCACCCTCCTCGCTATCCACCCTACCGAGGCTCGTCACAAGCAGAAGATCGTCGCTCCTGTCAAGCAGACCCTCAACTTCGACCTCCTCAAGCTCGCTGGTGACGTCGAGTCTAACCCTGGTCCTGATGGAAGAGCTTCCTCG |
| TNT-F2A_RW | 156 | CGAGGAAGCTCTTCCATCAGGACCAGGGTTAGACTCGACGTCACCAGCGAGCTTGAGGAGGTCGAAGTTGAGGGTCTGCTTGACAGGAGCGACGATCTTCTGCTTGTGACGAGCCTCGGTAGGGTGGATAGCGAGGAGGGTGGAAGAGCTGCATGT |
| TNT-Cas9-FW1 | 42 | ACATGCAGCTCTTCCACCATGGATTACAAGGATGATGATGAT |
| TNT-Cas9-RW1 | 35 | GAA TCG AAA AGA AGT GCA CCG ATA AGG |
| TNT-Cas9-FW2 | 27 | CCTTATCGGTGCACTTCTTTTCGATTC |
| TNT-Cas9-RW2 | 33 | ACT CGT AAA GAA GTG AGT GCT TTG G |
| TNT-Cas9-FW3 | 25 | CCAAAGCACTCACTTCTTTACGAGT |
| TNT-Cas9-RW3 | 30 | TGC TCG TGA AGT GAA TCT CCC TG |
| TNT-Cas9-FW4 | 23 | CAGGGAGATTCACTTCACGAGCA |
| TNT-Cas9-RW4 | 31 | CTT AGA TGG AAG TGC AAG CTC GTT |
| TNT-Cas9-FW5 | 24 | AACGAGCTTGCACTTCCATCTAAG |
| TNT-Cas9-RW5 | 45 | CGAGGAAGCTCTTCCATCTTT ATG CCT GCA GGT CGC GAG |
| Ω2-lefCC-FW | 51 | CGAGCTGCATGCCTGCAGCCCTCTTCAAGTACCCGAAGAGCGGCTTAACTA |
| Ω2-lefCC-RW | 51 | TAGTTAAGCCGCTCTTCGGGTACTTGAAGAGGGCTGCAGGCATGCAGCTCG |
| ΩC-lefCC-FW | 51 | CGAGCTGCATGCCTGCAGCCCTCTTCAAATACCCGAAGAGCGGCTTAACTA |
| ΩC-lefCC-RW | 51 | TAGTTAAGCCGCTCTTCGGGTATTTGAAGAGGGCTGCAGGCATGCAGCTCG |
| Ω1R-lefCC-FW | 51 | CGAGCTGCATGCCTGCAGCCCTCTTCAACCATCCGAAGAGCGGCTTAACTA |
| Ω1R-lefCC-RW | 51 | TAGTTAAGCCGCTCTTCGGATGGTTGAAGAGGGCTGCAGGCATGCAGCTCG |
| Ω2R-lefCC-FW | 51 | CGAGCTGCATGCCTGCAGCCCTCTTCAAGTATCCGAAGAGCGGCTTAACTA |
| Ω2R-lefCC-RW | 51 | TAGTTAAGCCGCTCTTCGGATACTTGAAGAGGGCTGCAGGCATGCAGCTCG |
| ΩB-rigCC-FW | 84 | GTTTGCGTATGCTCTTCGGATAATAGAAGAGGGGGATCAGATTGTCGTTTCCCGCCTTCAGTTTAAACTATCAGTGTTTGACAG |
| ΩB-rigCC-RW | 84 | CTGTCAAACACTGATAGTTTAAACTGAAGGCGGGAAACGACAATCTGATCCCCCTCTTCTATTATCCGAAGAGCATACGCAAAC |
| Ω1R-rigCC-FW | 84 | GTTTGCGTATGCTCTTCGGGTAGTAGAAGAGGGGGATCAGATTGTCGTTTCCCGCCTTCAGTTTAAACTATCAGTGTTTGACAG |
| Ω1R-rigCC-RW | 84 | CTGTCAAACACTGATAGTTTAAACTGAAGGCGGGAAACGACAATCTGATCCCCCTCTTCTACTACCCGAAGAGCATACGCAAAC |
| Ω2R-rigCC-FW | 84 | GTTTGCGTATGCTCTTCGGGTGATAGAAGAGGGGGATCAGATTGTCGTTTCCCGCCTTCAGTTTAAACTATCAGTGTTTGACAG |
| Ω2R-rigCC-RW | 84 | CTGTCAAACACTGATAGTTTAAACTGAAGGCGGGAAACGACAATCTGATCCCCCTCTTCTATCACCCGAAGAGCATACGCAAAC |
| rGUS-FW1 | 39 | ACATGCAGCTCTTCCACCATGTTACGTCCTGTAGAAACC |
| rGUS-RW1 | 29 | CGA GCA TCT CCT CAG CGT AAG G |
| rGUS-FW2 | 29 | CCT TAC GCT GAG GAG ATG CTC G |
| rGUS-RW2 | 29 | TGA CTG CCT CCT CGC TGT ACA G |
| rGUS-FW3 | 29 | CTG TAC AGC GAG GAG GCA GTC A |
| rGUS-RW3 | 30 | ACA CTG ATA CTC CTC ACT CCA CA |
| rGUS-FW4 | 30 | TGT GGA GTG AGG AGT ATC AGT GT |
| rGUS-RW4 | 47 | CGAGGAAGCTCTTCCATCTTG TTT GCC TCC CTG CTG CGG T |
| Hig-CodA-FW1 | 39 | ACATGCAGCTCTTCCACCATGAAAAAGCCTGAACTCACC |
| Hig-CodA-RW1 | 29 | CAC AGC CCC TCC TCG CCT GGT A |
| Hig-CodA-FW2 | 29 | TAC CAG GCG AGG AGG GGC TGT G |
| Hig-CodA-RW2 | 29 | CGT AAC GCC TCC TCC AGC AAC G |
| Hig-CodA-Fw3 | 29 | CGT TGC TGG AGG AGG CGT TAC G |
| Hig-CodA-RW3 | 42 | CGAGGAAGCTCTTCCATCACGTTTGTAATCGATGGCTTCTGG |

| | | |
|---|---|---|
| Luc+_pUPD_FW1 | 42 | ACATGCAGCTCTTCCACCATGGAAGATGCCAAAAACATAAAG |
| Luc+_pUPD_RW1 | 27 | GGG CGT ATC TTT TCA TAG CCT |
| Luc+_pUPD_FW2 | 27 | AGG CTA TGA AAA GAT ACG CCC |
| Luc+_pUPD_RW2 | 33 | AAG AAT TGA AGT GAG TTT TCA CTG C |
| Luc+_pUPD_FW3 | 33 | GCA GTG AAA ACT CAC TTC AAT TCT T |
| Luc+_pUPD_RW3 | 34 | CCT CAG AAA CAG TTC TTC TTC AAA TC |
| Luc+_pUPD_FW4 | 34 | GAT TTG AAG AAG AAC TGT TTC TGA GG |
| CircRep-FW | 39 | ACATGCAGCTCTTCCACCGAGGGCGGTCCGCTGCCTTTT |
| CircRep-RW | 39 | CGAGGAAGCTCTTCCATCCCTTGTTTGCCTGGCGGCAGT |
| TaqI-Fw | 29 | AAC CGT CTA TCA GGG CGA TGG C |
| TaqI-Rw | 29 | GGC TTT CCA CTT CCC CGA AAC C |
| TaqI-Fw1.1 | 27 | CGCAAGCTTGGATCGAAGAGCTCTTAG |
| TaqI-Rw1.1 | 27 | CTAAGAGCTCTTCGATCCAAGCTTGCG |
| 15ntW-H.TFOs1 | 15 | AATTTGTCGCTTCTC |
| 22ntW-H.TFOs1 | 21 | AGCCAGAATTTGTCGCTTCTC |
| 15ntRvH.TFOs1 | 15 | CTCTTCGCTGTTTAA |
| 22ntRvH.TFOs1 | 22 | CTCTTCGCTGTTTAAGACCGAT |
| 15ntW-H.TFOs2 | 15 | GTTCACTGTCTTCTC |
| 22ntW-H.TFOs2 | 22 | CCGGCGTGTTCACTGTCTTCTC |
| 15ntRvH.TFOs2 | 15 | CTCTTCTGTCACTTG |
| 22ntRvH.TFOs2 | 22 | CTCTTCTGTCACTTGTGCGGCC |
| 26RvH.DNAsyn8mDW | 26 | CACTGCCAGTTGCTCTTCATATAGCA |
| 26RvH.DNAsyn8mDW-Acridine 3' | 26 | CACTGCCAGTTGCTCTTCATATAGCA-Acridine |
| 5'-Acridine-DNA$_{(n)}$/BNA$^{NC}_{(n+)}$ | 14 | Acridine-G+C+C+A+G+T+T+GCT+CT+TC |
| TFOsynt_Uni5’ | 225 | ACATGCAGCTCTTCCACCAAACTCATAACAGGGAACTATAATTAGGACTAAAGAAGATTCAACGTACATTGATCTGACACA GTAGATTTAGTTGTCTCTTGTACATACACAGTATCTAGGATTATTCAACGAAAACAATATCAATTGTCTCTACAGAAACCAAC GGCCAGTACTCTTTTGCCCTAAAAAGACCGTAACCCTAATTGTCACACTGAGAATCTAACG |
| TFOsynt_Uni3’ | 316 | TAGCAGATGCTACGATCTGTCAGCAACTGAGAAGTCTATTTGCTTTTGTGATTCAGGAATATGCTGAATTCCTGCACGAATT CATGTGCGCTGTAAAGCAGAACTATGGAGAGAAAGTGTTGGTTCAGGTGAGCCATAGGATACTCTCTTAAGAACTATGATT GTTGTCAGAACTACGATAAAAGATGTCCGGAATTAATATCATACACTCATCTTTTCAGTTTGAAGATTTTGCAAACCACAAT GCGTTTGACCTTTTGTCTAAGTACAGTGATAGCTTTCTGCCACTTGTTGTATCGATGGAAGAGCTTCCTCG |
| TFOsynt_8m1 | 210 | GTCACACTGAGAATCTAACGATTGCCAGTTACTCTTCTGGGACCTACGACGAAGGATGACTCCGTCCACGTTCTTCTTCACT GTTTGACAATAAGCTCCAATTTTCAGACTTTTCATTTCAAACTTGTGGGTCTCATTTTCCTCTGGCCTATATAAATCCACTATC CTCCACTGCCAGTTGCTCTTCATATAGCAGATGCTACGATCTGT |
| TFOsynt_8m2 | 210 | GTCACACTGAGAATCTAACGATTAGGTCCCGCTCTTCTGGGACCTACGACGAAGGATGACTCCGTCCACGTTCTTCTTCACT GTTTGACAATAAGCTCCAATTTTCAGACTTTTCATTTCAAACTTGTGGGTCTCATTTTCCTCTGGCCTATATAAATCCACTATC CTCCACTGCCAGTTGCTCTTCATATAGCAGATGCTACGATCTGT |
| TFOsynt_7m1 | 210 | GTCACACTGAGAATCTAACGATTGCCAGTTACTCTTCTGGGACCTACGACGAAGGATGACTCCGTCCACGTTCTTCTTCACT GTTTGACAATAAGCTCCAATTTTCAGACTTTTCATTTCAAACTTGTGGGTCTCATTTTCCTCTGGCCTATATAAATCCACTATC CTCCACTGCCAGTTCCTCTTCATATAGCAGATGCTACGATCTGT |
| TFOsynt_5m2 | 210 | GTCACACTGAGAATCTAACGATTGCCAGACTCTCTTCTGGGACCTACGACGAAGGATGACTCCGTCCACGTTCTTCTTCACT GTTTGACAATAAGCTCCAATTTTCAGACTTTTCATTTCAAACTTGTGGGTCTCATTTTCCTCTGGCCTATATAAATCCACTATC CTCCACTATGAGTTGCTCTTCATATAGCAGATGCTACGATCTGT |

| | | |
|---|---|---|
| TFOsynt_4m1 | 210 | GTCACACTGAGAATCTAACGATTGGCTGCCACTCTTCTGGGACCTACGACGAAGGATGACTCCGTCCACGTTCTTCTTCACT<br>GTTTGACAATAAGCTCCAATTTTCAGACTTTTCATTTCAAACTTGTGGGTCTCATTTTCCTCTGGCCTATATAAATCCACTATC<br>CTCCACTGGCTGCCGCTCTTCATATAGCAGATGCTACGATCTGT |
| LacZw-central-gb | 1960 | AACGCTGCTTCGGCCTGGTAATGGCCCGCCGCCTTCCAGCGTTCGACCCAGGCGTTAGGGTCAATGCGGGTCGCTTCACTT<br>ACGCCAATGTCGTTATCCAGCGGTGCACGGGTGAACTGATCGCGCAGCGGCGTCAGCAGTTGTTTTTTATCGCCAATCCAC<br>ATCTGTGAAAGAAAGCCTGACTGGCGGTTAAATTGCCAACGCTTATTACCCAGCTCGATGCAAAAATCCATTTCGCTGGTG<br>GTCAGATGCGGGATGGCGTGGGACGCGGCGGGGAGCGTCACACTGAGGTTTTCCGCCAGACGCCACTGCTGCCAGGCGCT<br>GATGTGCCCGGCTTCTGACCATGCGGTCGCGTTCGGTTGCACTACGCGTACTGTGAGCCAGAGTTGCCCGGCGCTCTCCGG<br>CTGCGGTAGTTCAGGCAGTTCAATCAACTGTTTACCTTGTGGAGCGACATCCAGAGGCACTTCACCGCTTGCCAGCGGCTTA<br>CCATCCAGCGCCACCATCCAGTGCAGGAGCTCGTTATCGCTATGACGGAACAGGTATTCGCTGGTCACTTCGATGGTTTGCC<br>CGGATAAACGGAACTGGAAAAACTGCTGCTGGTGTTTTGCTTCCGTCAGCGCTGGATGCGGCGTGCGGTCGGCAAAGACC<br>AGACCGTTCATACAGAACTGGCGATCGTTCGGCGTATCGCCAAAATCACCGCCGTAAGCCGACCACGGGTTGCCGTTTTCA<br>TCATATTTAATCAGCGACTGATCCACCCAGTCCCAGACGAAGCCGCCCTGTAAACGGGGATACTGACGAAACGCCTGCCAG<br>TATTTAGCGAAACCGCCAAGACTGTTACCCATCGCGTGGGCGTATTCGCAAAGGATCAGCGGGCGCGTCTCTCCAGGTAGC<br>GAAAGCCATTTTTTGATGGACCATTTCGGCACAGCCGGGAAGGGCTGGTCTTCATCCACGCGCGCGTACATCGGGCAAATA<br>ATATCGGTGGCCGTGGTGTCGGCTCCGCCGCCTTCATACTGCACCGGGCGGGAAGGATCGACAGATTTGATCCAGCGATAC<br>AGCGCGTCGTGATTAGCGCCGTGGCCTGATTCATTCCCCAGCGACCAGATGATCACACTCGGGTGATTACGATCGCGCTGC<br>ACCATTCGCGTTACGCGTTCGCTCATCGCCGGTAGCCAGCGCGGATCATCGGTCAGACGATTCATTGGCACCATGCCGTGG<br>GTTTCAATATTGGCTTCATCCACCACATACAGGCCGTAGCGGTCGCACAGCGTGTACCACAGCGGATGGTTCGGATAATGC<br>GAACAGCGCACGGCGTTAAAGTTGTTCTGCTTCATCAGCAGGATATCCTGCACCATCGTCTGCTCATCCATGACCTGACCAT<br>GCAGAGGATGATGCTCGTGACGGTTAACGCCTCGAATCAGCAACGGCTTGCCGTTCAGCAGCAGCAGACCATTTTCAATCC<br>GCACCTCGCGGAAACCGACATCGCAGGCTTCTGCTTCAATCAGCGTGCCGTCGGCGGTGTGCAGTTCAACCACCGCACGAT<br>AGAGATTCGGGATTTCGGCGCTCCACAGTTTCGGGTTTTCGACGTTCAGACGTAGTGTGACGCGATCGGCATAACCACCAC<br>GCTCATCGATAATTTCACCGCCGAAAGGCGCGGTGCCGCTGGCGACCTGCGTTTCACCCTGCCATAAAGAAACTGTTACCC<br>GTAGGTAGTCACGCAACTCGCCGCACATCTGAACTTCAGCCTCCAGTACAGCGCGGCTGAAATCATCATTAAAGCGAGTGG<br>CAACATGGAAATCGCTGATTTGTGTAGTCGGTTTATGCAGCAACGAGACGTCACGGAAAATGCCGCTCATCCGCCACATAT<br>CCTGATCTTCCAGATAACTGCCGTCACTCCAGCGCAGCACCATCACCGCGAGGCGGTTTTCTCCGGCGCGTAAAAATGCGCT<br>CAGGTCAAATTCAG |
| alphaBR-gb left | 696 | ATTCGAGCTGCATGCCTGCAGGCTCTTCGAGTATCAGAAGAGTGGCTTAACTATGCGGCATCAGAGCTTATTTTTGACACCA<br>GACCAACTGGTAATGGTAGCGACCGGCGCTCAGCTGGAATTCCGCCGATACTGACGGGCTCCAGGAGTCGTCGCCACCAA<br>TCCCCATATGGAAACCGTCGATATTCAGCCATGTGCCTTCTTCCGCGTGCAGCAGATGGCGATGGCTGGTTTCCATCAGTTG<br>CTGTTGACTGTAGCGGCTGATGTTGAACTGGAAGTCGCCGCGCCACTGGTGTGGGCCATAATTCAATTCGCGCGTCCCGCA<br>GCGCAGACCGTTTTCGCTCGGGAAGACGTACGGGGTATACATGTCTGACAATGGCAGATCCCAGCGGTCAAAACAGGCGG<br>CAGTAAGGCGGTCGGGATAGTTTTCTTGCGGCCCTAATCCGAGCCAGTTTACCCGCTCTGCTACCTGCGCCAGCTGGCAGTT<br>CAGGCCAATCCGCGCCGGATGCGGTGTATCGCTCGCCACTTCAACATCAACGGTAATCGCCATTTGACCACTACCATCAATC<br>CGGTAGGTTTTCCGGCTGATAAATAAGGTTTTCCCCTGATGCTGCCACGCGTGAGCGGTCGTAATCAGCACCGCATCAGCA<br>AGTGTATCTGCCGTGCACTGCAACAACGCTGCTTCGGCCTGGTAAT |

| | | |
|---|---|---|
| alphaBR-gb right | 718 | AATGCGCTCAGGTCAAATTCAGACGGCAAACGACTGTCCTGGCCGTAACCGACCCAGCGCCCGTTGCACCACAGATGAAAC<br>GCCGAGTTAACGCCATCAAAAATAATTCGCGTCTGGCCTTCCTGTAGCCAGCTTTCATCAACATTAAATGTGAGCGAGTAAC<br>AACCCGTCGGATTCTCCGTGGGAACAAACGGCGGATTGACCGTAATGGGATAGGTCACGTTGGTGTAGATGGGCGCATCG<br>TAACCGTGCATCTGCCAGTTTGAGGGGACGACGACAGTATCGGCCTCAGGAAGATCGCACTCCAGCCAGCTTTCCGGCACC<br>GCTTCTGGTGCCGGAAACCAGGCAAAGCGCCATTCGCCATTCAGGCTGCGCAACTGTTGGGAAGGGCGATCGGTGCGGGC<br>CTCGACGGCCAGTGAATCCGTAATCATGGTCATAGCTGTTTCTGTGTGAAATTGTTATCCGCTCACAATTCCACACAACATA<br>CGAGCCGGAAGCATAAAGTGTAAAGCCTGGGGTGCCTAATGAGTGAGCTAACTCACATTAATTGCGTTGCGCTCACTGCCC<br>GCTTTCCAGTCGGGAAACCTGTCGTGCCAGCTGCATTAATGAATCGGCCAACGCGCGGGGAGAGGCGGTTTGCGTATACTC<br>TTCAGGTAATCGAAGAGCTGGATCAGATTGTCGTTTCCCGCCTTCAGTTTAAACTATCAGTGTTTGACAG |
| alphaCR-gb left | 696 | ATTCGAGCTGCATGCCTGCAGGCTCTTCGAATATCAGAAGAGTGGCTTAACTATGCGGCATCAGAGCTTATTTTTGACACCA<br>GACCAACTGGTAATGGTAGCGACCGGCGCTCAGCTGGAATTCCGCCGATACTGACGGGCTCCAGGAGTCGTCGCCACCAA<br>TCCCCATATGGAAACCGTCGATATTCAGCCATGTGCCTTCTTCCGCGTGCAGCAGATGGCGATGGCTGGTTTCCATCAGTTG<br>CTGTTGACTGTAGCGGCTGATGTTGAACTGGAAGTCGCCGCGCCACTGGTGTGGGCCATAATTCAATTCGCGCGTCCCGCA<br>GCGCAGACCGTTTTCGCTCGGGAAGACGTACGGGGTATACATGTCTGACAATGGCAGATCCCAGCGGTCAAAACAGGCGG<br>CAGTAAGGCGGTCGGGATAGTTTTCTTGCGGCCCTAATCCGAGCCAGTTTACCCGCTCTGCTACCTGCGCCAGCTGGCAGTT<br>CAGGCCAATCCGCGCCGGATGCGGTGTATCGCTCGCCACTTCAACATCAACGGTAATCGCCATTTGACCACTACCATCAATC<br>CGGTAGGTTTTCCGGCTGATAAATAAGGTTTTCCCCTGATGCTGCCACGCGTGAGCGGTCGTAATCAGCACCGCATCAGCA<br>AGTGTATCTGCCGTGCACTGCAACAACGCTGCTTCGGCCTGGTAAT |
| alphaCR-gb right | 718 | AATGCGCTCAGGTCAAATTCAGACGGCAAACGACTGTCCTGGCCGTAACCGACCCAGCGCCCGTTGCACCACAGATGAAAC<br>GCCGAGTTAACGCCATCAAAAATAATTCGCGTCTGGCCTTCCTGTAGCCAGCTTTCATCAACATTAAATGTGAGCGAGTAAC<br>AACCCGTCGGATTCTCCGTGGGAACAAACGGCGGATTGACCGTAATGGGATAGGTCACGTTGGTGTAGATGGGCGCATCG<br>TAACCGTGCATCTGCCAGTTTGAGGGGACGACGACAGTATCGGCCTCAGGAAGATCGCACTCCAGCCAGCTTTCCGGCACC<br>GCTTCTGGTGCCGGAAACCAGGCAAAGCGCCATTCGCCATTCAGGCTGCGCAACTGTTGGGAAGGGCGATCGGTGCGGGC<br>CTCGACGGCCAGTGAATCCGTAATCATGGTCATAGCTGTTTCTGTGTGAAATTGTTATCCGCTCACAATTCCACACAACATA<br>CGAGCCGGAAGCATAAAGTGTAAAGCCTGGGGTGCCTAATGAGTGAGCTAACTCACATTAATTGCGTTGCGCTCACTGCCC<br>GCTTTCCAGTCGGGAAACCTGTCGTGCCAGCTGCATTAATGAATCGGCCAACGCGCGGGGAGAGGCGGTTTGCGTATACTC<br>TTCAGGTGATCGAAGAGCTGGATCAGATTGTCGTTTCCCGCCTTCAGTTTAAACTATCAGTGTTTGACAG |
| omegaBR-gb left | 697 | ATTCGAGCTGCATGCCTGCAGCCCTCTTCAAGTATCCGAAGAGCGGCTTAACTATGCGGCATCAGAGCTTATTTTTGACACC<br>AGACCAACTGGTAATGGTAGCGACCGGCGCTCAGCTGGAATTCCGCCGATACTGACGGGCTCCAGGAGTCGTCGCCACCA<br>ATCCCCATATGGAAACCGTCGATATTCAGCCATGTGCCTTCTTCCGCGTGCAGCAGATGGCGATGGCTGGTTTCCATCAGTT<br>GCTGTTGACTGTAGCGGCTGATGTTGAACTGGAAGTCGCCGCGCCACTGGTGTGGGCCATAATTCAATTCGCGCGTCCCGC<br>AGCGCAGACCGTTTTCGCTCGGGAAGACGTACGGGGTATACATGTCTGACAATGGCAGATCCCAGCGGTCAAAACAGGCG<br>GCAGTAAGGCGGTCGGGATAGTTTTCTTGCGGCCCTAATCCGAGCCAGTTTACCCGCTCTGCTACCTGCGCCAGCTGGCAG<br>TTCAGGCCAATCCGCGCCGGATGCGGTGTATCGCTCGCCACTTCAACATCAACGGTAATCGCCATTTGACCACTACCATCAA<br>TCCGGTAGGTTTTCCGGCTGATAAATAAGGTTTTCCCCTGATGCTGCCACGCGTGAGCGGTCGTAATCAGCACCGCATCAGC<br>AAGTGTATCTGCCGTGCACTGCAACAACGCTGCTTCGGCCTGGTAAT |

| | | |
|---|---|---|
| omegaBR-gb right | 718 | AATGCGCTCAGGTCAAATTCAGACGGCAAACGACTGTCCTGGCCGTAACCGACCCAGCGCCCGTTGCACCACAGATGAAAC<br>GCCGAGTTAACGCCATCAAAAATAATTCGCGTCTGGCCTTCCTGTAGCCAGCTTTCATCAACATTAAATGTGAGCGAGTAAC<br>AACCCGTCGGATTCTCCGTGGGAACAAACGGCGGATTGACCGTAATGGGATAGGTCACGTTGGTGTAGATGGGCGCATCG<br>TAACCGTGCATCTGCCAGTTTGAGGGGACGACGACAGTATCGGCCTCAGGAAGATCGCACTCCAGCCAGCTTTCCGGCACC<br>GCTTCTGGTGCCGGAAACCAGGCAAAGCGCCATTCGCCATTCAGGCTGCGCAACTGTTGGGAAGGGCGATCGGTGCGGGC<br>CTCGACGGCCAGTGAATCCGTAATCATGGTCATAGCTGTTTCTGTGTGAAATTGTTATCCGCTCACAATTCCACACAACATA<br>CGAGCCGGAAGCATAAAGTGTAAAGCCTGGGGTGCCTAATGAGTGAGCTAACTCACATTAATTGCGTTGCGCTCACTGCCC<br>GCTTTCCAGTCGGGAAACCTGTCGTGCCAGCTGCATTAATGAATCGGCCAACGCGCGGGGAGAGGCGGTTTGCGTATGCTC<br>TTCGGGTAATAGAAGAGGGGGATCAGATTGTCGTTTCCCGCCTTCAGTTTAAACTATCAGTGTTTGACAG |
| omegaCR-gb left | 697 | ATTCGAGCTGCATGCCTGCAGCCCTCTTCAAATATCCGAAGAGCGGCTTAACTATGCGGCATCAGAGCTTATTTTTGACACC<br>AGACCAACTGGTAATGGTAGCGACCGGCGCTCAGCTGGAATTCCGCCGATACTGACGGGCTCCAGGAGTCGTCGCCACCA<br>ATCCCCATATGGAAACCGTCGATATTCAGCCATGTGCCTTCTTCCGCGTGCAGCAGATGGCGATGGCTGGTTTCCATCAGTT<br>GCTGTTGACTGTAGCGGCTGATGTTGAACTGGAAGTCGCCGCGCCACTGGTGTGGGCCATAATTCAATTCGCGCGTCCCGC<br>AGCGCAGACCGTTTTCGCTCGGGAAGACGTACGGGGTATACATGTCTGACAATGGCAGATCCCAGCGGTCAAAACAGGCG<br>GCAGTAAGGCGGTCGGGATAGTTTTCTTGCGGCCCTAATCCGAGCCAGTTTACCCGCTCTGCTACCTGCGCCAGCTGGCAG<br>TTCAGGCCAATCCGCGCCGGATGCGGTGTATCGCTCGCCACTTCAACATCAACGGTAATCGCCATTTGACCACTACCATCAA<br>TCCGGTAGGTTTTCCGGCTGATAAATAAGGTTTTCCCCTGATGCTGCCACGCGTGAGCGGTCGTAATCAGCACCGCATCAGC<br>AAGTGTATCTGCCGTGCACTGCAACAACGCTGCTTCGGCCTGGTAAT |
| omegaCR-gb right | 718 | AATGCGCTCAGGTCAAATTCAGACGGCAAACGACTGTCCTGGCCGTAACCGACCCAGCGCCCGTTGCACCACAGATGAAAC<br>GCCGAGTTAACGCCATCAAAAATAATTCGCGTCTGGCCTTCCTGTAGCCAGCTTTCATCAACATTAAATGTGAGCGAGTAAC<br>AACCCGTCGGATTCTCCGTGGGAACAAACGGCGGATTGACCGTAATGGGATAGGTCACGTTGGTGTAGATGGGCGCATCG<br>TAACCGTGCATCTGCCAGTTTGAGGGGACGACGACAGTATCGGCCTCAGGAAGATCGCACTCCAGCCAGCTTTCCGGCACC<br>GCTTCTGGTGCCGGAAACCAGGCAAAGCGCCATTCGCCATTCAGGCTGCGCAACTGTTGGGAAGGGCGATCGGTGCGGGC<br>CTCGACGGCCAGTGAATCCGTAATCATGGTCATAGCTGTTTCTGTGTGAAATTGTTATCCGCTCACAATTCCACACAACATA<br>CGAGCCGGAAGCATAAAGTGTAAAGCCTGGGGTGCCTAATGAGTGAGCTAACTCACATTAATTGCGTTGCGCTCACTGCCC<br>GCTTTCCAGTCGGGAAACCTGTCGTGCCAGCTGCATTAATGAATCGGCCAACGCGCGGGGAGAGGCGGTTTGCGTATGCTC<br>TTCGGGTGATAGAAGAGGGGGATCAGATTGTCGTTTCCCGCCTTCAGTTTAAACTATCAGTGTTTGACAG |
| RGR gene | 247 | ACATGCAGCTCTTCCaccGGGTTACTGATGAGTCCGTGAGGACGAAACGAGTAAGCTCGTCTAACCCAAGGGGTGACAAGC<br>GTTTTAGAGCTAGAAATAGCAAGTTAAAATAAGGCTAGTCCGTTATCAACTTGAAAAAGTGGCACCGAGTCGGTGCTTTTG<br>GCCGGCATGGTCCCAGCCTCCTCGCTGGCGCCGGCTGGGCAACATGCTTCGGCATGGCGAATGGGACgatGGAAGAGCTTC<br>CTCG |

| | | |
|---|---|---|
| M.SacI cluster | 1924 | TCAGATCCCGGGTCAATAGCATTCTCACCAATAAAAAACGCCCGGCGGCAACCGAGCGTTCTGAACAAATCCAGATGGAGTTCTGAGGTCATTA<br>CTGGATCTATCAACAGGAGTCCAAGCGGCCGCTTTTTTTACCTCCTAAAAGTTAAACAAAATTATTTCTAGAGGGAAACCGTTGTGGAATTGTGA<br>GCGCTCACAATTCCACATATTATAATTGTTATCCGCTCACAAAGCAAATAAATTTTTCATGATTTCACTGTGCATGAAGCTCGTAATTGTTATCCGC<br>TCACAATTAAACAAGCGCTCATGAGCCCGAAGTGGCGAGCCCGATCTTCCCCATCGGTGATGTCGGCGATATAGGCGCCAGCAACCGCACCTG<br>TGGCGCCGGTGATGCCGGCCACGATGCGTCCGGCGTAGAGGATCGAGATCTCGATCCCGCGAAATTAATACGACTCACTATAGGGGAATTGTG<br>AGCGGATAACAATTCCCCTCTAGAAATAATTTTGTTTAACTTTAAGAAGGAGATATACCATGGGTCTGCCTCCGCTGCTGAGCCTGCCGAGCAAT<br>AGCGCACCGCGTAGCCTGGGTCGTGTTGAAACCCCTCCGGAAGTTGTTGATTTTATGGTTAGCCTGGCCGAAGCACCGCGTGGTGGTCGTGTTC<br>TGGAACCGGCATGTGCCCATGGTCCGTTTCTGCGTGCATTTCGTGAAGCACATGGCACCGGTTATCGTTTTGTTGGTGTTGAAATTGATCCGAAA<br>GCACTGGATCTGCCACCGTGGGCAGAAGGTATTCTGGCAGATTTTCTGCTGTGGGAACCGGGTGAAGCATTTGATCTGATTCTGGGTAATCCGC<br>CTTATGGTATTGTTGGTGAAGCAAGCAAATATCCGATCCATGTTTTTAAAGCCGTGAAAGATCTGTACAAAAAAGCCTTTAGCACCTGGAAAGG<br>CAAATATAACCTGTATGGTGCCTTTCTGGAAAAAGCAGTTCGTCTGCTGAAACCGGGTGGTGTTCTGGTTTTTGTTGTTCCGGCAACCTGGCTGG<br>TGCTGGAAGATTTTGCACTGCTGCGTGAATTTCTGGCACGTGAAGGTAAAACCAGCGTTTATTATCTGGGTGAAGTTTTTCCGCAGAAAAAAGT<br>TAGCGCAGTGGTTATTCGTTTTCAGAAAAGCGGTAAAGGTCTGAGCCTGTGGGATACCCAAGAAAGCGAAAGCGGTTTTACCCCGATTCTGTG<br>GGCTGAATATCCGCATTGGGAAGGTGAAATTATTCGCTTTGAAACCGAAGAAACCCGCAAACTGGAAATTTCAGGTATGCCGCTGGGTGACCT<br>GTTTCATATCCGTTTTGCAGCACGTAGTCCGGAATTCAAAAAACATCCGGCAGTTCGTAAAGAACCGGGTCCGGGTCTGGTTCCGGTTCTGACC<br>GGTCGTAATCTGAAACCTGGTTGGGTTGATTATGAAAAAAATCATAGCGGTCTGTGGATGCCGAAAGAACGTGCAAAAGAACTGCGTGATTTT<br>TATGCAACACCGCATCTGGTTGTTGCACATACCAAAGGCACCCGTGTTGTTGCAGCATGGGATGAACGTGCATATCCGTGGCGTGAAGAATTTC<br>ATCTGCTGCCTAAAGAAGGTGTTCGTCTGGATCCGAGCAGCCTGGTTCAGTGGCTGAATAGCGAAGCAATGCAGAAACATGTTCGTACCCTGTA<br>TCGTGATTTTGTTCCGCATCTGACCCTGCGTATGCTGGAACGTCTGCCGGTTCGTCGTGAATATGGTTTTCATACCAGTCCGGAAAGCGCACGTA<br>ACTTTTAACAAAGCCCGAAAGGAAGCTGAGTTGGCTGCTGCCACCGCTGAGCAATAACTAGCATAACCCCTTGGGGCCTCTAAACGGGTCTTGA<br>GGGGTTTTTTGCTGAAAGGAGGCCACTCGAGCACCTAGGAG |

**Supplementary Table 4.** Insert sequences for pSTART clones.

| *Name (5'->3' description)* | *Length* | *Sequence* |
|---|---|---|
| d35S_h-h (2x35S Promoter antisense, 35S Promoter sense) | 2001 | CATGGCTATCGTTCGTAAATGGTGAAAATTTTCAGAAAATTGCTTTTGCTTTAAAAGAAATGATTTAAATTGCTGCAATAGAAGTAGAATGCTTGATTGCTT<br>GAGATTCGTTTGTTTTGTATATGTTGTGTTGAGAATTCTCGAGGTCCGATGACTGGTGATTTCAGCGTGTCCTCTCCAAATGAAATGAACTTCCTTATATAGA<br>GGAAGGGTCTTGCGAAGGATAGTGGGATTGTGCGTCATCCCTTACGTCAGTGGAGATATCACATCAATCCACTTGCTTTGAAGACGTGGTTGGAACGTCTT<br>CTTTTTCCACGATGCTCCTCGTGGGTGGGGGTCCATCTTTGGGACCACTGTCGGCAGAGGCATCTTGAACGATAGCCTTTCCTTTATCGCAATGATGGCATT<br>TGTAGGTGCCACCTTCCTTTTCTACTGTCCTTTTGATGAAGTGACAGATAGCTGGGCAATGGAATCCGAGGAGGTTTCCCGATATTACCCTTTGTTGAAAAG<br>TCTCAATAGCCCTTTGGTCTTCTGAGACTGTATCTTTGATATTCTTGGAGTAGACGAGAGTGTCGTGCTCCACCATGTTCACATCAATCCACTTGCTTTGAAG<br>ACGTGGTTGGAACGTCTTCTTTTTCCACGATGCTCCTCGTGGGTGGGGGTCCATCTTTGGGACCACTGTCGGCAGAGGCATCTTGAACGATAGCCTTTCCTT<br>TATCGCAATGATGGCATTTGTAGGTGCCACCTTCCTTTTCTACTGTCCTTTTGATGAAGTGACAGATAGCTGGGCAATGGAATCCGAGGAGGTTTCCCGATA<br>TTACCCTTTGTTGAAAAGTCTCAATAGCCCTTTGGTCTTCTGAGACTGTATCTTTGATATTCTTGGAGTAGACGAGAGTGTCGTGCTCCACCATGTTGGCAAG<br>CTGCTCTAGCCAATACGCAAACCGCCTCTCCCCGCGCGTTGGCCGATTCATTAATGCAGCTGGCACGACAGGTTTCCCGACTGGAAAGCGGGCAGTGAGCG<br>CAACGCAATTAATGTGAGTTAGCTCACTCATTAGGCACCCCAGGCTTTACACTTTATGCTTCCGGCTCGTATGTTGTGTGGAATTGTGAGCGGATAACAATT<br>TCACACAGGAAACAGCTATGACATGATTACGAATTCGGTCCCCAGATTAGCCTTTTCAATTTCAGAAAGAATGCTAACCCACAGATGGTTAGAGAGGCTTA<br>CGCAGCAGGTCTCATCAAGACGATCTACCCGAGCAATAATCTCCAGGAAATCAAATACCTTCCCAAGAAGGTTAAAGATGCAGTCAAAAGATTCAGGACTA<br>ACTGCATCAAGAACACAGAGAAAGATATATTTCTCAAGATCAGAAGTACTATTCCAGTATGGACGATTCAAGGCTTGCTTCACAAACCAAGGCAAGTAATA<br>GAGATTGGAGTCTCTAAAAAGGTAGTTCCCACTGAATCAAAGGCCATGGAGTCAAAGATTCAAATAGAGGACCTAACAGAACTCGCCGTAAAGACTGGCG<br>AACAGTTCATACAGAGTCTCTTACGACTCAATGACAAGAAGAAAATCTTCGTCAACATGGTGGAGCACGACACACTTGTCTACTCCAAAAATATCAAAGATA<br>CAGTCTCAGAAGACCAAAGGGCAATTGAGACTTTTCAACAAAGGGTAATATCCGGAAACCTCCTCGGATTCCATTGCCCAGCTATCTGTCACTTTATTGTGA<br>AGATAGTGGAAAAGGAAGGTGGCTCCTACAAATGCCATCATTGCGATAAAGGAAAGGCCATCGTTGAAGATGCCTCTGCCGACAGTGGTCCCAAAGATG<br>GACCCCCACCCACGAGGAGCATCGTGGAAAAAGAAGACGTTCCAACCACGTCTTCAAAGCAAGTGGATTGATGTGATATCTCCACTGACGTAAGGGATGA<br>CGCACAATCCCACTATCCTTCGCAAGACCCTTCCTCTATATAAGGAAGTTCATTTCATTTGGAGAGAACACG |
| PIP2A-mCherry (Plasm membrane protein PIP2A fused to mCherry) | 1579 | ATGGCAAAGGATGTGGAAGCCGTTCCCGGAGAAGGATTTCAGACAAGAGACTATCAAGATCCGCCACCAGCTCCGTTTATTGATGGAGCGGAG<br>CTAAAGAAGTGGTCTTTCTACAGAGCAGTTATCGCAGAGTTCGTAGCCACTCTCCTATTCTTATACATCACCGTTTTGACAGTCATCGGTTACAAG<br>ATTCAGTCCGATACTGATGCCGGTGGCGTAGATTGCGGCGGAGTTGGAATCCTCGGTATCGCTTGGGCCTTTGGTGGTATGATCTTCATCCTCGT<br>CTACTGCAC CGCCGGTATCTCTGGTGGTCACATTAACCCAGCGGTGACATTTGGGCTATTCTTGGCACGTAAAGTGTCGTTACCTAGGGCCCTAT<br>TGTACATAATCGCTCAGTGTTTGGGTGCGATTTGTGGAGTTGGTTTTGTCAAAGCCTTCCAAAGCTCTTACTACACCCGTTACGGAGGTGGAGCC<br>AACTCTCTAGCCGATGGCTACAGCACAGGGACCGGTCTAGCCGCAGAGATCATTGGTACTTTCGTTCTTGTCTACACCGTCTTCTCTGCCACTGA<br>CCCCAAACGTAGTGCCAGAGACTCCCACGTTCCGGTGTTGGCGCCACTTCCAATCGGATTTGCCGTGTTCATGGTACATTTGGCTACCATTCCCA<br>TTACCGGAACCGGAATTAACCCGGCAAGGAGTTTCGGAGCTGCCGTAATCTACAACAAGAGCAAGCCATGGGATGACCACTGGATATTTTGGGT<br>TGGACCATTCATTGGAGCTGCGATAGCTGCATTCTACCACCAATTCGTTCTAAGAGCTTCAGGTTCTAAGTCTCTTGGATCATTCAGAAGTGCTGC<br>CAACGTCGGATCCATGGTGAGCAAGGGCGAGGAGGATAACATGGCCATCATCAAGGAGTTCATGCGCTTCAAGGTGCACATGGAGGGCTCCGT<br>GAACGGCCACGAGTTCGAGATCGAGGGCGAGGGCGAGGGCCGCCCCTACGAGGGCACCCAGACCGCCAAGCTGAAGGTGACCAAGGGTGGC<br>CCCCTGCCCTTCGCCTGGGACATCCTGTCCCCTCAGTTCATGTACGGCTCCAAGGCCTACGTGAAGCACCCCGCCGACATCCCCGACTACTTGAA<br>GCTGTCCTTCCCCGAGGGCTTCAAGTGGGAGCGCGTGATGAACTTCGAGGACGGCGGCGTGGTGACCGTGACCCAGGACTCCTCCCTGCAGGA<br>CGGCGAGTTCATCTACAAGGTGAAGCTGCGCGGCACCAACTTCCCCTCCGACGGCCCCGTAATGCAGAAGAAGACCATGGGCTGGGAGGCCTC<br>CTCCGAGCGGATGTACCCCGAGGACGGCGCCCTGAAGGGCGAGATCAAGCAGAGGCTGAAGCTGAAGGACGGCGGCCACTACGACGCTGAGG<br>TCAAGACCACCTACAAGGCCAAGAAGCCCGTGCAGCTGCCCGGCGCCTACAACGTCAACATCAAGTTGGACATCACCTCCCACAACGAGGACTA<br>CACCATCGTGGAACAGTACGAACGCGCCGAGGGCCGCCACTCCACCGGCGGCATGGACGAGCTGTACAGATCT |
| Lumio (Invitrogen Tag) | 36 | GGTGCTGGTGGTTGCTGCCCTGGTTGCTGCGGTGGT |
| P2A (PTV1; viral protein) | 57 | GCTACCAACTTCTCTCTCCTCAAGCAGGCTGGTGACGTCGAGGAGAACCCTGGTCCT |
| T2A (TAV;viral protein) | 60 | CGTGCTGAGGGTCGTGGTTCTCTCCTCACCTGCGGTGACGTCGAGGAGAACCCTGGTCCT |
| F2A (FMDV;viral protein) | 120 | CTCCTCGCTATCCACCCTACCGAGGCTCGTCACAAGCAGAAGATCGTCGCTCCTGTCAAGCAGACCCTCAACTTCGACCTCCTCAAGCTCGCTGG<br>TGACGTCGAGTCTAACCCTGGTCCT |

| | | |
|---|---|---|
| Cas9* (CRISPR associated protein 9) | 4755 | ATGGATTACAAGGATGATGATGATAAGGATTACAAGGATGATGATGATAAGATGGCTCCAAAGAAGAAGAGAAAGGTTGGAATCCACGGAGTTCCAGCTGCTGATAAGAAGTACTCTATCGGA<br>CTTGACATCGGAACCAACTCTGTTGGATGGGCTGTTATCACCGATGAGTACAAGGTTCCATCTAAGAAGTTCAAGGTTCTTGGAAACACCGATAGACACTCTATCAAGAAGAACCTTATCGGTGC<br>aCTTCTTTTCGATTCTGGAGAGACCGCTGAGGCTACCAGATTGAAGAGAACCGCTAGAAGAAGATACACCAGAAGAAAGAACAGAATCTGCTACCTTCAGGAAATCTTCTCTAACGAGATGGCT<br>AAGGTTGATGATTCTTTCTTCCACAGACTTGAGGAGTCTTTCCTTGTTGAGGAGGATAAGAAGCACGAGAGACACCCAATCTTCGGAAACATCGTTGATGAGGTTGCTTACCACGAGAAGTACCC<br>AACCATCTACCACCTTAGAAAGAAGTTGGTTGATTCTACCGATAAGGCTGATCTTAGACTTATCTACCTTGCTCTTGCTCACATGATCAAGTTCAGAGGACACTTCCTTATCGAGGGAGACCTTAAC<br>CCAGATAACTCTGATGTTGATAAGTTGTTCATCCAGCTTGTTCAGACCTACAACCAGCTTTTCGAGGAGAACCCAATCAACGCTTCTGGAGTTGATGCTAAGGCTATCCTTTCTGCTAGACTTTCTA<br>AGTCTCGTAGACTTGAGAACCTTATCGCTCAGCTTCCAGGAGAGAAGAAGAACGGACTTTTCGGAAACCTTATCGCTCTTTCTCTTGGACTTACCCCAAACTTCAAGTCTAACTTCGATCTTGCTGA<br>GGATGCTAAGTTGCAGCTTTCTAAGGATACCTACGATGATGATCTTGATAACCTTCTTGCTCAGATCGGAGATCAGTACGCTGATCTTTTCCTTGCTGCTAAGAACCTTTCTGATGCTATCCTTCTTT<br>CTGACATCCTTAGAGTTAACACCGAGATCACCAAGGCTCCACTTTCTGCTTCTATGATCAAGAGATACGATGAGCACCACCAGGATCTTACCCTTTTGAAGGCTCTTGTTAGACAGCAGCTTCCAG<br>AGAAGTACAAGGAAATCTTCTTCGATCAGTCTAAGAACGGATACGCTGGATACATCGATGGAGGAGCTTCTCAGGAGGAGTTCTACAAGTTCATCAAGCCAATCCTTGAGAAGATGGATGGAAC<br>CGAGGAGCTTCTTGTTAAGTTGAACAGAGAGGATCTTCTTAGAAAGCAGAGAACCTTCGATAACGGATCTATCCCACACCAGATCCACCTTGGAGAGCTTCACGCTATCCTTCGTAGACAGGAGG<br>ATTTCTACCCATTCTTGAAGGATAACAGAGAGAAGATCGAGAAGATCCTTACCTTCAGAATCCCATACTACGTTGGACCACTTGCTAGAGGAAACTCTCGTTTCGCTTGGATGACCAGAAAGTCTG<br>AGGAGACCATCACCCCTTGGAACTTCGAGGAGGTAAGTTTCTGCTTCTACCTTTGATATATATATAATAATTATCATTAATTAGTAGTAATATAATATTTCAAATATTTTTTTCAAAATAAAAGAATG<br>TAGTATATAGCAATTGCTTTTCTGTAGTTTATAAGTGTGTATATTTTAATTTATAACTTTTCTAATATATGACCAAAATTTGTTGATGTGCAGGTTGTTGATAAGGGAGCTTCTGCTCAGTCTTTCATC<br>GAGAGAATGACCAACTTCGATAAGAACCTTCCAAACGAGAAGGTTCTTCCAAAGCACTCaCTTCTTTACGAGTACTTCACCGTTTACAACGAGCTTACCAAGGTTAAGTACGTTACCGAGGGAAT<br>GAGAAAGCCAGCTTTCCTTTCTGGAGAGCAGAAGAAGGCTATCGTTGATCTTCTTTTCAAGACCAACAGAAAGGTTACCGTTAAGCAGTTGAAGGAGGATTACTTCAAGAAGATCGAGTGCTTCG<br>ATTCTGTTGAAATCTCTGGAGTTGAGGATAGATTCAACGCTTCTCTTGGAACCTACCACGATCTTTTGAAGATCATCAAGGATAAGGATTTCCTTGATAACGAGGAGAACGAGGACATCCTTGAG<br>GACATCGTTCTTACCCTTACCCTTTTCGAGGATAGAGAGATGATCGAGGAGAGACTCAAGACCTACGCTCACCTTTTCGATGATAAGGTTATGAAGCAGTTGAAGAGAAGAAGATACACCGGAT<br>GGGGTAGACTTTCTCGTAAGTTGATCAACGGAATCAGAGATAAGCAGTCTGGAAAGACCATCCTTGATTTCTTGAAGTCTGATGGATTCGCTAACAGAAACTTCATGCAGCTTATCCACGATGATT<br>CTCTTACCTTCAAGGAGGACATCCAGAAGGCTCAGGTTTCTGGACAGGGAGATTCaCTTCACGAGCACATCGCTAACCTTGCTGGATCTCCAGCTATCAAGAAGGGAATCCTTCAGACCGTTAAG<br>GTTGTTGATGAGCTTGTTAAGGTTATGGGTAGACACAAGCCAGAGAACATCGTTATCGAGATGGCTAGAGAGAACCAGACCACCCAGAAGGGACAGAAGAACTCTCGTGAGAGAATGAAGAG<br>AATCGAGGAGGGAATCAAGGAGCTTGGATCTCAAATCTTGAAGGAGCACCCAGTTGAGAACACCCAGCTTCAGAACGAGAAGTTGTACCTTTACTACCTTCAGAACGGAAGAGATATGTACGTT<br>GATCAGGAGCTTGACATCAACAGACTTTCTGATTACGATGTTGATCACATCGTTCCACAGTCTTTCTTGAAGGATGATTCTATCGATAACAAGGTTCTTACCCGTTCTGATAAGAACAGAGGAAAG<br>TCTGATAACGTTCCATCTGAGGAGGTTGTTAAGAAGATGAAGAACTACTGGAGACAGCTTCTTAACGCTAAGTTGATCACCCAGAGAAAGTTCGATAACCTTACCAAGGCTGAGAGAGGAGGAC<br>TTTCTGAGCTTGATAAGGCTGGATTCATCAAGAGACAGCTTGTTGAGACCAGACAGATCACCAAGCACGTTGCTCAGATCCTTGATTCTCGTATGAACACCAAGTACGATGAGAACGATAAGTTG<br>ATCAGAGAGGTTAAGGTTATCACCTTGAAGTCTAAGTTGGTTTCTGATTTCAGAAAGGATTTCCAGTTCTACAAGGTTAGAGAGATCAACAACTACCACCACGCTCACGATGCTTACCTTAACGCT<br>GTTGTTGGAACCGCTCTTATCAAGAAGTACCCAAAGTTGGAGTCTGAGTTCGTTTACGGAGATTACAAGGTTTACGATGTTAGAAAGATGATCGCTAAGTCTGAGCAGGAGATCGGAAAGGCTA<br>CCGCTAAGTACTTCTTCTACTCTAACATCATGAACTTCTTCAAGACCGAGATCACCCTTGCTAACGGAGAGATCAGAAAGAGACCACTTATCGAGACCAACGGAGAGACCGGAGAGATCGTTTGG<br>GATAAGGGAAGAGATTTCGCTACCGTTAGAAAGGTTCTTTCTATGCCACAGGTTAACATCGTTAAGAAAACCGAGGTTCAGACCGGAGGATTCTCTAAGGAGTCTATCCTTCCAAAGAGAAACTC<br>TGATAAGTTGATCGCTAGAAAGAAGGATTGGGACCCAAAGAAGTACGGAGGATTCGATTCTCCAACCGTTGCTTACTCTGTTCTTGTTGTTGCTAAGGTTGAGAAGGGAAAGTCTAAGAAGTTGA<br>AGTCTGTTAAGGAGCTTCTTGGAATCACCATCATGGAGCGTTCTTCTTTCGAGAAGAACCCAATCGATTTCCTTGAGGCTAAGGGATACAAGGAGGTTAAGAAGGATCTTATCATCAAGTTGCCA<br>AAGTACTCTCTTTTCGAGCTTGAGAACGGAAGAAAGAGAATGCTTGCTTCTGCTGGAGAGCTTCAGAAGGGAAACGAGCTTGCaCTTCCATCTAAGTACGTTAACTTCCTTTACCTTGCTTCTCACT<br>ACGAGAAGTTGAAGGGATCTCCAGAGGATAACGAGCAGAAGCAGCTTTTCGTTGAGCAGCACAAGCACTACCTTGATGAGATCATCGAGCAAATCTCTGAGTTCTCTAAGAGAGTTATCCTTGC<br>TGATGCTAACCTTGATAAGGTTCTTTCTGCTTACAACAAGCACAGAGATAAGCCAATCAGAGAGCAGGCTGAGAACATCATCCACCTTTTCACCCTTACCAACCTTGGTGCTCCAGCTGCTTTCAA<br>GTACTTCGATACCACCATCGATAGAAAAAGATACACCTCTACCAAGGAGGTTCTTGATGCTACCCTTATCCACCAGTCTATCACCGGACTTTACGAGACCAGAATCGATCTTTCTCAGCTTGGAGG<br>AGATAAGAGACCAGCTGCTACCAAGAAGGCTGGACAGGCTAAGAAGAAGAAGTGACTGCAGATCGTTCAAACATTTGGCAATAAAGTTTCTTAAGATTGAATCCTGTTGCCGGTCTTGCGATGA<br>TTATCATATAATTTCTGTTGAATTACGTTAAGCATGTAATAATTAACATGTAATGCATGACGTTATTTATGAGATGGGTTTTTATGATTAGAGTCCCGCAATTATACATTTAATACGCGATAGAAAAC<br>AAAATATAGCGCGCAACTAGGATAAATTATCGCGCGCGGTGTCATCTATGTTACTAGATCAGGCCTGTCGACGCCCGGGCGGTACCGCGATCGCTCGCGACCTGCAGGCATAAA |
| Ibp (Impatiens balsamina peptide) | 54 | CCTTGCTCTAACGCTGCTGACGAGGTCGCTACCCCTGAGGACGTCGAGCCTGGT |
| GFP (Green fluorescent protein) | 714 | GTGAGCAAGGGCGAGGAGCTGTTCACCGGGGTGGTGCCCATCCTGGTCGAGCTGGACGGCGACGTAAACGGCCACAAGTTCAGCGTGTCCGG<br>CGAGGGCGAGGGCGATGCCACCTACGGCAAGCTGACCCTGAAGTTCATCTGCACCACCGGCAAGCTGCCCGTGCCCTGGCCCACCCTCGTGAC<br>CACCCTGACCTACGGCGTGCAGTGCTTCAGCCGCTACCCCGACCACATGAAGCAGCACGACTTCTTCAAGTCCGCCATGCCCGAAGGCTACGTC<br>CAGGAGCGCACCATCTTCTTCAAGGACGACGGCAACTACAAGACCCGCGCCGAGGTGAAGTTCGAGGGCGACACCCTGGTGAACCGCATCGAG<br>CTGAAGGGCATCGACTTCAAGGAGGACGGCAACATCCTGGGGCACAAGCTGGAGTACAACTACAACAGCCACAACGTCTATATCACCGCCGAC<br>AAGCAGAAGAACGGCATCAAGGCCAACTTCAAGATCCGCCACAACATCGAGGACGGCGGCGTGCAGCTCGCCGACCACTACCAGCAGAACACC<br>CCCATCGGCGACGGCCCCGTGCTGCTGCCCGACAACCACTACCTGAGCTACCAGTCCGCCCTGAGCAAAGACCCCAACGAGAAGCGCGATCACA<br>TGGTCCTGCTGGAGTTCGTGACCGCCGCCGGGATCACTCTCGGCATGGACGAGCTGTACAAG |
| 35SProm (35SPromoter sense) | 1099 | CACAACATACGAGCCGGAAGCATAAAGTGTAAAGCCTGGGGTGCCTAATGAGTGAGCTAACTCACATTAATTGCGTTGCGCTCACTGCCCGCTT<br>TCCAGTCGGGAAACCTGTCGTGCCAGCTGCATTAATGAATCGGCCAACGCGCGGGGAGAGGCGGTTTGCGTATTGGCTAGAGCAGCTTGCCAA<br>CATGGTGGAGCACGACACTCTCGTCTACTCCAAGAATATCAAAGATACAGTCTCAGAAGACCAAAGGGCTATTGAGACTTTTCAACAAAGGGTA<br>ATATCGGGAAACCTCCTCGGATTCCATTGCCCAGCTATCTGTCACTTCATCAAAAGGACAGTAGAAAAGGAAGGTGGCACCTACAAATGCCATC<br>ATTGCGATAAAGGAAAGGCTATCGTTCAAGATGCCTCTGCCGACAGTGGTCCCAAAGATGGACCCCCACCCACGAGGAGCATCGTGGAAAAAG<br>AAGACGTTCCAACCACGTCTTCAAAGCAAGTGGATTGATGTGAACATGGTGGAGCACGACACTCTCGTCTACTCCAAGAATATCAAAGATACAG<br>TCTCAGAAGACCAAAGGGCTATTGAGACTTTTCAACAAAGGGTAATATCGGGAAACCTCCTCGGATTCCATTGCCCAGCTATCTGTCACTTCATC<br>AAAAGGACAGTAGAAAAGGAAGGTGGCACCTACAAATGCCATCATTGCGATAAAGGAAAGGCTATCGTTCAAGATGCCTCTGCCGACAGTGGT<br>CCCAAAGATGGACCCCCACCCACGAGGAGCATCGTGGAAAAAGAAGACGTTCCAACCACGTCTTCAAAGCAAGTGGATTGATGTGATATCTCCA<br>CTGACGTAAGGGATGACGCACAATCCCACTATCCTTCGCAAGACCCTTCCTCTATATAAGGAAGTTCATTTCATTTGGAGAGGACACGCTGAAAT<br>CACCAGTCATCGGACCTCGAGAATTCTCAACACAACATATACAAAACAAACGAATCTCAAGCAATCAAGCATTCTACTTCTATTGCAGCAATTTAA<br>ATCATTTCTTTTAAAGCAAAAGCAATTTTCTGAAAATTTTCACCATTTACGAACGATAGCCATG |

| | | |
|---|---|---|
| 35STerm (35S Terminator sense) | 291 | TAAGTAGCTGAATCCCGCGGCCATGCTAGAGTCCGCAAAAATCACCAGTCTCTCTCTACAAATCTATCTCTCTCTATTTTTCTCCAGAATAATGTG<br>TGAGTAGTTCCCAGATAAGGGAATTAGGGTTCTTATAGGGTTTCGCTCATGTGTTGAGCATATAAGAAACCCTTAGTATGTATTTGTATTTGTAA<br>AATACTTCTATCAATAAAATTTCTAATTCCTAAAACCAAAATCCAGTGACCTGCAGGCATGCGACGTCGGGCCTAGCCCGAGACGTCGGGCCTAG<br>CCCGA |
| NLS (Nuclear localization signal) | 30 | CCTAAGAAGAAGCGTAAGGTCGAGGACCCT |
| NosProm (Nos Promoter sense) | 321 | TGATCATGAGCGGAGAATTAAGGGAGTCACGTTATGACCCCCGCCGATGACGCGGGACAAGCCGTTTTACGTTTGGAACTGACAGAACCGCAA<br>CGATTGAAGGAGCCACTCAGCCGCGGGTTTCTGGAGTTTAATGAGCTAAGCACATACGTCAGAAACCATTATTGCGCGTTCAAAAGTCGCCTAA<br>GGTCACTATCAGCTAGCAAATATTTCTTGTCAAAAATGCTCCACTGACGTTCCATAAATTCCCCTCGGTATCCAATTAGAGTCTCATATTCACTCTC<br>AATCCAAATAATCTGCACCGGATCTGGATCGTTTCGC |
| rGUS ( β-glucuronidase) | 1806 | ATGTTACGTCCTGTAGAAACCCCAACCCGTGAAATCAAAAAACTCGACGGCCTGTGGGCATTCAGTCTGGATCGCGAAAACTGTGGAATTGATC<br>AGCGTTGGTGGGAAAGCGCGTTACAAGAAAGCCGGGCAATTGCTGTGCCAGGCAGTTTTAACGATCAGTTCGCCGATGCAGATATTCGTAATTA<br>TGCGGGCAACGTCTGGTATCAGCGCGAAGTCTTTATACCGAAAGGTTGGGCAGGCCAGCGTATCGTGCTGCGTTTCGATGCGGTCACTCATTAC<br>GGCAAAGTGTGGGTCAATAATCAGGAAGTGATGGAGCATCAGGGCGGCTATACGCCATTTGAAGCCGATGTCACGCCGTATGTTATTGCCGGG<br>AAAAGTGTACGTATCACCGTTTGTGTGAACAACGAACTGAACTGGCAGACTATCCCGCCGGGAATGGTGATTACCGACGAAAACGGCAAGAAA<br>AAGCAGTCTTACTTCCATGATTTCTTTAACTATGCCGGAATCCATCGCAGCGTAATGCTCTACACCACGCCGAACACCTGGGTGGACGATATCAC<br>CGTGGTGACGCATGTCGCGCAAGACTGTAACCACGCGTCTGTTGACTGGCAGGTGGTGGCCAATGGTGATGTCAGCGTTGAACTGCGTGATGC<br>GGATCAACAGGTGGTTGCAACTGGACAAGGCACTAGCGGGACTTTGCAAGTGGTGAATCCGCACCTCTGGCAACCGGGTGAAGGTTATCTCTAT<br>GAACTGTGCGTCACAGCCAAAAGCCAGACAGAGTGTGATATCTACCCGCTTCGCGTCGGCATCCGGTCAGTGGCAGTGAAGGGCCAACAGTTC<br>CTGATTAACCACAAACCGTTCTACTTTACTGGCTTTGGTCGTCATGAAGATGCGGACTTACGTGGCAAAGGATTCGATAACGTGCTGATGGTGCA<br>CGACCACGCATTAATGGACTGGATTGGGGCCAACTCCTACCGTACCTCGCATTACCCTTACGCTGAGGAGATGCTCGACTGGGCAGATGAACAT<br>GGCATCGTGGTGATTGATGAAACTGCTGCTGTCGGCTTTAACCTCTCTTTAGGCATTGGTTTCGAAGCGGGCAACAAGCCGAAAGAACTGTACA<br>GCGAGGAGGCAGTCAACGGGGAAACTCAGCAAGCGCACTTACAGGCGATTAAAGAGCTGATAGCGCGTGACAAAAACCACCCAAGCGTGGTG<br>ATGTGGAGTATTGCCAACGAACCGGATACCCGTCCGCAAGTGCACGGGAATATTTCGCCACTGGCGGAAGCAACGCGTAAACTCGACCCGACG<br>CGTCCGATCACCTGCGTCAATGTAATGTTCTGCGACGCTCACACCGATACCATCAGCGATCTCTTTGATGTGCTGTGCCTGAACCGTTATTACGG<br>ATGGTATGTCCAAAGCGGCGATTTGGAAACGGCAGAGAAGGTACTGGAAAAAGAACTTCTGGCCTGGCAGGAGAAACTGCATCAGCCGATTAT<br>CATCACCGAATACGGCGTGGATACGTTAGCCGGGCTGCACTCAATGTACACCGACATGTGGAGTGAGGAGTATCAGTGTGCATGGCTGGATAT<br>GTATCACCGCGTCTTTGATCGCGTCAGCGCCGTCGTCGGTGAACAGGTATGGAATTTCGCCGATTTTGCGACCTCGCAAGGCATATTGCGCGTTG<br>GCGGTAACAAGAAAGGGATCTTCACTCGCGACCGCAAACCGAAGTCGGCGGCTTTTCTGCTGCAAAAACGCTGGACTGGCATGAACTTCGGTG<br>AAAAACCGCAGCAGGGAGGCAAACAA |
| HCC (Hygromycin resistance and CodA genes fused by F2A viral protein) | 2390 | CCACCATGAAAAAGCCTGAACTCACCGCGACGTCTGTCGAGAAGTTTCTGATCGAAAAGTTCGACAGCGTCTCCGACCTGATGCAGCTCTCGGAGGGCGAAGAATCTC<br>GTGCTTTCAGCTTCGATGTAGGAGGGCGTGGATATGTCCTGCGGGTAAATAGCTGCGCCGATGGTTTCTACAAAGATCGTTATGTTTATCGGCACTTTGCATCGGCCGC<br>GCTCCCGATTCCGGAAGTGCTTGACATTGGGGAGTTTAGCGAGAGCCTGACCTATTGCATCTCCCGCCGTGCACAGGGTGTCACGTTGCAAGACCTGCCTGAAACCGAA<br>CTGCCCGCTGTTCTACAACCGGTCGCGGAGGCTATGGATGCGATCGCTGCGGCCGATCTTAGCCAGACGAGCGGGTTCGGCCCATTCGGACCGCAAGGAATCGGTCAA<br>TACACTACATGGCGTGATTTCATATGCGCGATTGCTGATCCCCATGTGTATCACTGGCAAACTGTGATGGACGACACCGTCAGTGCGTCCGTCGCGCAGGCTCTCGATG<br>AGCTGATGCTTTGGGCCGAGGACTGCCCCGAAGTCCGGCACCTCGTGCACGCGGATTTCGGCTCCAACAATGTCCTGACGGACAATGGCCGCATAACAGCGGTCATTG<br>ACTGGAGCGAGGCGATGTTCGGGGATTCCCAATACGAGGTCGCCAACATCTTCTTCTGGAGGCCGTGGTTGGCTTGTATGGAGCAGCAGACGCGCTACTTCGAGCGGA<br>GGCATCCGGAGCTTGCAGGATCGCCACGACTCCGGGCGTATATGCTCCGCATTGGTCTTGACCAACTCTATCAGAGCTTGGTTGACGGCAATTTCGATGATGCAGCTTG<br>GGCGCAGGGTCGATGCGACGCAATCGTCCGATCCGGAGCCGGGACTGTCGGGCGTACACAAATCGCCCGCAGAAGCGCGGCCGTCTGGACCGATGGCTGTGTAGAAG<br>TACTCGCCGATAGTGGAAACCGACGCCCCAGCACTCGTCCGAGGGCAAAGAAAGCGGCCGCAGTGAAGCAGACCCTTAATTTTGATCTTTTGAAGCTCGCTGGTGATG<br>TTGAGTCTAATCCAGGTCCTGGATCCATGTCGAATAACGCTTTACAAACAATTATTAACGCCCGGTTACCAGGCGAGGAGGGGCTGTGGCAGATTCATCTGCAGGACG<br>GAAAAATCAGCGCCATTGATGCGCAATCCGGCGTGATGCCCATAACTGAAAACAGCCTGGATGTCGAACAAGGTTTAGTTATACCGCCGTTTGTGGAGCCACATATTCA<br>CCTGGACACCACGCAAACCGCCGGACAACCGAACTGGAATCAGTCCGGCACGCTGTTTGAAGGCATTGAACGCTGGGCCGAGCGCAAAGCGTTATTAACCCATGACGA<br>TGTGAAACAACGCGCATGGCAAACGCTGAAATGGCAGATTGCCAACGGCATTCAGCATGTGCGTACCCATGTCGATGTTTCGGATGCAACGCTAACTGCGCTGAAAGC<br>AATGCTGGAAGTGAAGCAGGAAGTCGCGCCGTGGATTGATCTGCAAATCGTCGCCTTCCCTCAGGAAGGGATTTTGTCGTATCCCAACGGTGAAGCGTTGCTGGAGGA<br>GGCGTTACGCTTAGGGGCAGATGTAGTGGGGGCGATTCCGCATTTTGAATTTACCCGTGAATACGGCGTGGAGTCGCTGCATAAAACCTTCGCCCTGGCGCAAAAATA<br>CGACCGTCTCATCGACGTTCACTGTGATGAGATCGATGACGAGCAGTCGCGCTTTGTCGAAACCGTTGCTGCCCTGGCGCACCATGAAGGCATGGGCGCGCGAGTCAC<br>CGCCAGCCACACCACGGCAATGCACTCCTATAACGGGGCGTATACCTCACGCCTGTTCCGCTTGCTGAAAATGTCCGGTATTAACTTTGTCGCCAACCCGCTGGTCAATA<br>TTCATCTGCAAGGACGTTTCGATACGTATCCAAAACGTCGCGGCATCACGCGCGTTAAAGAGATGCTGGAGTCCGGCATTAACGTCTGCTTTGGTCACGATGATGTCTT<br>CGATCCGTGGTATCCGCTGGGAACGGCGAATATGCTGCAAGTGCTGCATATGGGGCTGCATGTTTGCCAGTTGATGGGCTACGGGCAGATTAACGATGGCCTGAATTT<br>AATCACCCACCACAGCGCAAGGACGTTGAATTTGCAGGATTACGGCATTGCCGCCGGAAACAGCGCCAACCTGATTATCCTGCCGGCTGAAAATGGGTTTGATGCGCT<br>GCGCCGTCAGGTTCCGGTACGTTATTCGGTACGTGGCGGCAAGGTGATTGCCAGCACACAACCGGCACAAACCACCGTATATCTGGAGCAGCCAGAAGCCATCGATTA<br>CAAACGT |

| | | |
|---|---|---|
| Kan-ORF (Kanamycin resistance gene CDS) | 792 | ATGATTGAACAAGATGGATTGCACGCAGGTTCTCCGGCCGCTTGGGTGGAGAGGCTATTCGGCTATGACTGGGCACAACAGACAATCGGCTGC<br>TCTGATGCCGCCGTGTTCCGGCTGTCAGCGCAGGGGCGCCCGGTTCTTTTTGTCAAGACCGACCTGTCCGGTGCCCTGAATGAACTGCAGGACG<br>AGGCAGCGCGGCTATCGTGGCTGGCCACGACGGGCGTTCCTTGCGCAGCTGTGCTCGACGTTGTCACTGAAGCGGGAAGGGACTGGCTGCTAT<br>TGGGCGAAGTGCCGGGGCAGGATCTCCTGTCATCTCACCTTGCTCCTGCCGAGAAAGTATCCATCATGGCTGATGCAATGCGGCGGCTGCATAC<br>GCTTGATCCGGCTACCTGCCCATTCGACCACCAAGCGAAACATCGCATCGAGCGAGCACGTACTCGGATGGAAGCCGGTCTTGTCGATCAGGAT<br>GATCTGGACGAGGAACATCAGGGGCTCGCGCCAGCCGAACTGTTCGCCAGGCTCAAGGCGCGCATGCCCGACGGCGAGGATCTCGTCGTGACC<br>CATGGCGATGCCTGCTTGCCGAATATCATGGTGGAAAATGGCCGCTTTTCTGGATTCATCGACTGTGGCCGGCTGGGTGTGGCGGACCGCTATC<br>AGGACATAGCGTTGGCTACCCGTGATATTGCTGAGGAACTTGGCGGCGAATGGGCTGACCGCTTCCTCGTGCTTTACGGTATCGCCGCTCCCGA<br>TTCGCAGCGCATCGCCTTCTATCGCCTTCTTGACGAGTTCTTC |
| 8m1* (synthetic DNA) | 675 | AAACTCATAACAGGGAACTATAATTAGGACTAAAGAAGATTCAACGTACATTGATCTGACACAGTAGATTTAGTTGTCTCTTGTACATACACAGT<br>ATCTAGGATTATTCAACGAAAACAATATCAATTGTCTCTACAGAAACCAACGGCCAGTACTCTTTTGCCCTAAAAAGACCGTAACCCTAATTGTCA<br>CACTGAGAATCTAACGATTGCCAGTTACTCTTCTGGGACCTACGACGAAGGATGACTCCGTCCACGTTCTTCTTCACTGTTTGACAATAAGCTCCA<br>ATTTTCAGACTTTTCATTTCAAACTTGTGGGTCTCATTTTCCTCTGGCCTATATAAATCCACTATCCTCCACTGCCAGTTGCTCTTCATATAGCAGAT<br>GCTACGATCTGTCAGCAACTGAGAAGTCTATTTGCTTTTGTGATTCAGGAATATGCTGAATTCCTGCACGAATTCATGTGCGCTGTAAAGCAGAA<br>CTATGGAGAGAAAGTGTTGGTTCAGGTGAGCCATAGGATACTCTCTTAAGAACTATGATTGTTGTCAGAACTACGATAAAAGATGTCCGGAATT<br>AATATCATACACTCATCTTTTCAGTTTGAAGATTTTGCAAACCACAATGCGTTTGACCTTTTGTCTAAGTACAGTGATAGCTTTCTGCCACTTGTTG<br>TATC |
| 8m2*(synthetic DNA) | 675 | AAACTCATAACAGGGAACTATAATTAGGACTAAAGAAGATTCAACGTACATTGATCTGACACAGTAGATTTAGTTGTCTCTTGTACATACACAGT<br>ATCTAGGATTATTCAACGAAAACAATATCAATTGTCTCTACAGAAACCAACGGCCAGTACTCTTTTGCCCTAAAAAGACCGTAACCCTAATTGTCA<br>CACTGAGAATCTAACGATTAGGTCCCGCTCTTCTGGGACCTACGACGAAGGATGACTCCGTCCACGTTCTTCTTCACTGTTTGACAATAAGCTCC<br>AATTTTCAGACTTTTCATTTCAAACTTGTGGGTCTCATTTTCCTCTGGCCTATATAAATCCACTATCCTCCACTGCCAGTTGCTCTTCATATAGCAGA<br>TGCTACGATCTGTCAGCAACTGAGAAGTCTATTTGCTTTTGTGATTCAGGAATATGCTGAATTCCTGCACGAATTCATGTGCGCTGTAAAGCAGA<br>ACTATGGAGAGAAAGTGTTGGTTCAGGTGAGCCATAGGATACTCTCTTAAGAACTATGATTGTTGTCAGAACTACGATAAAAGATGTCCGGAAT<br>TAATATCATACACTCATCTTTTCAGTTTGAAGATTTTGCAAACCACAATGCGTTTGACCTTTTGTCTAAGTACAGTGATAGCTTTCTGCCACTTGTT<br>GTATC |
| 7m1*(synthetic DNA) | 675 | AAACTCATAACAGGGAACTATAATTAGGACTAAAGAAGATTCAACGTACATTGATCTGACACAGTAGATTTAGTTGTCTCTTGTACATACACAGT<br>ATCTAGGATTATTCAACGAAAACAATATCAATTGTCTCTACAGAAACCAACGGCCAGTACTCTTTTGCCCTAAAAAGACCGTAACCCTAATTGTCA<br>CACTGAGAATCTAACGATTGCCAGTTACTCTTCTGGGACCTACGACGAAGGATGACTCCGTCCACGTTCTTCTTCACTGTTTGACAATAAGCTCCA<br>ATTTTCAGACTTTTCATTTCAAACTTGTGGGTCTCATTTTCCTCTGGCCTATATAAATCCACTATCCTCCACTGCCAGTTCCTCTTCATATAGCAGAT<br>GCTACGATCTGTCAGCAACTGAGAAGTCTATTTGCTTTTGTGATTCAGGAATATGCTGAATTCCTGCACGAATTCATGTGCGCTGTAAAGCAGAA<br>CTATGGAGAGAAAGTGTTGGTTCAGGTGAGCCATAGGATACTCTCTTAAGAACTATGATTGTTGTCAGAACTACGATAAAAGATGTCCGGAATT<br>AATATCATACACTCATCTTTTCAGTTTGAAGATTTTGCAAACCACAATGCGTTTGACCTTTTGTCTAAGTACAGTGATAGCTTTCTGCCACTTGTTG<br>TATC |
| 5m2*(synthetic DNA) | 675 | AAACTCATAACAGGGAACTATAATTAGGACTAAAGAAGATTCAACGTACATTGATCTGACACAGTAGATTTAGTTGTCTCTTGTACATACACAGT<br>ATCTAGGATTATTCAACGAAAACAATATCAATTGTCTCTACAGAAACCAACGGCCAGTACTCTTTTGCCCTAAAAAGACCGTAACCCTAATTGTCA<br>CACTGAGAATCTAACGATTGCCAGACTCTCTTCTGGGACCTACGACGAAGGATGACTCCGTCCACGTTCTTCTTCACTGTTTGACAATAAGCTCCA<br>ATTTTCAGACTTTTCATTTCAAACTTGTGGGTCTCATTTTCCTCTGGCCTATATAAATCCACTATCCTCCACTATGAGTTGCTCTTCATATAGCAGAT<br>GCTACGATCTGTCAGCAACTGAGAAGTCTATTTGCTTTTGTGATTCAGGAATATGCTGAATTCCTGCACGAATTCATGTGCGCTGTAAAGCAGAA<br>CTATGGAGAGAAAGTGTTGGTTCAGGTGAGCCATAGGATACTCTCTTAAGAACTATGATTGTTGTCAGAACTACGATAAAAGATGTCCGGAATT<br>AATATCATACACTCATCTTTTCAGTTTGAAGATTTTGCAAACCACAATGCGTTTGACCTTTTGTCTAAGTACAGTGATAGCTTTCTGCCACTTGTTG<br>TATC |
| 4m1*(synthetic DNA) | 675 | AAACTCATAACAGGGAACTATAATTAGGACTAAAGAAGATTCAACGTACATTGATCTGACACAGTAGATTTAGTTGTCTCTTGTACATACACAGT<br>ATCTAGGATTATTCAACGAAAACAATATCAATTGTCTCTACAGAAACCAACGGCCAGTACTCTTTTGCCCTAAAAAGACCGTAACCCTAATTGTCA<br>CACTGAGAATCTAACGATTGGCTGCCACTCTTCTGGGACCTACGACGAAGGATGACTCCGTCCACGTTCTTCTTCACTGTTTGACAATAAGCTCC<br>AATTTTCAGACTTTTCATTTCAAACTTGTGGGTCTCATTTTCCTCTGGCCTATATAAATCCACTATCCTCCACTGGCTGCCGCTCTTCATATAGCAG<br>ATGCTACGATCTGTCAGCAACTGAGAAGTCTATTTGCTTTTGTGATTCAGGAATATGCTGAATTCCTGCACGAATTCATGTGCGCTGTAAAGCAG<br>AACTATGGAGAGAAAGTGTTGGTTCAGGTGAGCCATAGGATACTCTCTTAAGAACTATGATTGTTGTCAGAACTACGATAAAAGATGTCCGGAA<br>TTAATATCATACACTCATCTTTTCAGTTTGAAGATTTTGCAAACCACAATGCGTTTGACCTTTTGTCTAAGTACAGTGATAGCTTTCTGCCACTTGT<br>TGTATC |

| | | |
|---|---|---|
| CircRep (synthetic DNA) | 777 | GAGGGCGGTCCGCTGCCTTTTGCATTCGACATCCTGGCCCCGTGCTGTATGTACGGCTCTAAGACCTTCATTAAACACGTGAGCGGTATCCCGGA<br>TTACTTTAAAGAGTCCTTTCCAGAGGGCTTCACTTGGGAACGTACCCAGATTTTTGAGGACGGTGGTGTTCTGACCGCGCACCAAGACACCAGCC<br>TGGAAGGTAATTGCCTGATCTATAAAGTGAAGGTTCTGGGTACCAATTTCCCGGCGAATGGTCCGGTGATGCAAAAGAAAACCGCGGGTTGGG<br>AGCCGTGCGTCGAGATGCTGTATCCGCGTGACGGCGTCTTGTGTGGTCAGAGCTTGATGGCGCTGAAGTGCACCGATGGCAATCATCTGACCAG<br>CCACCTGCGCACGACGTATCGTAGCCGTAAACCGAGCAACGCCGTTAACATGCCGGAGTTCCATTTTGGTGACCATCGCATCGAAATCCTGAAA<br>GCTGAGCAGGGCAAATTCTACGAACAATACGAATCGGCTGTCGCACGTTACAGCGATGTGCCGGAAAAAGCGACGTAATGAGAATTCTGTACA<br>CTCGAGGGTCTCACCCCAAGGGCGACACCCCCTAATTAGCCCGGGCGAAAGGCCCAGTCTTTCGACTGAGCCTTTCGTTTTATTTGATGCCTGGC<br>AGTTCCCTACTCTCGCATGGGGAGTCCCCACACTACCATCGGCGCTACGGCGTTTCACTTCTGAGTTCGGCATGGGGTCAGGTGGGACCACCGC<br>GCTACTGCCGCCAGGCAAACAAGG |

Except for Cas9*, coding sequences have no STOP codons and Promoters end with ATG (d35S_h-h require a second ATG to be included if both directions are to be used for ORFs).

**Supplementary Table 5 -** Vendor, catalog number and specific reagents used in this study.

| *Vendor* | *Cat. No.* | *Product Description* |
|---|---|---|
| Fisher Scientific | FERER0231 | 10U/µL, 300U Thermo Scientific Eam1104I (EarI) |
| Sigma | B2904-100MG | 5-Bromo-3-indolyl β-D-galactopyranoside |
| Sigma | B4252-1G | 5-Bromo-4-chloro-3-indolyl β-D-galactopyranoside |
| Sigma | B4252-1G | 5-Bromo-4-chloro-3-indolyl β-D-galactopyranoside |
| Santa Cruz Biotechnology | sc-281503 | 8-Hydroxyquinoline-b-D-galactopyranoside |
| NEB | P0756S | Adenosine 5'-Triphosphate (ATP) |
| NEB | R0712L | BspQI |
| Qiagen | 19063 | Buffer QG (250 ml) |
| Life Technologies | 10177-012 | Carbenicillin, Disodium Salt |
| NEB | M0226S | CpG Methyltransferase M.SssI |
| GenScript | C01577-1 | dATP (100 mM) |
| GenScript | C01579-1 | dCTP (100 mM) |
| GenScript | C01578-1 | dGTP (100 mM) |
| Epoch Life Science Inc | 1920-250 | DNA purification colums |
| GenScript | C01580-1 | dTTP (100 mM) |
| NEB | R0528L | EarI |
| 5PRIME | 2300010 | FastPlasmid Mini Kit |
| FroggaBio | DF300 | Gel/PCR DNA Fragments Extraction Kit |
| Sigma | I6758-1G | IPTG |
| Life Technologies | 11815-032 | Kanamycin Sulfate |
| Sigma | A3256-25G | L-(+)-Arabinose |
| Thermo Scientific | # ER1932 | Lgu I |
| NEB | B7024S | Gel Loading Dye, Purple (6x) |
| LABREPCO | 11608031 | Micro Disposable Electroporation Chambers |
| NEB | B9007S | NAD |
| Sigma | N7878-25G | Nitrofurantoin |
| NEB | R3193S | NcoI-HF |
| NEB | R3189S | NotI-HF |
| Sigma | 435406 | PEG-PPG-PEG (PPG) |
| addgene | Plasmid 16666 | pGRG36 (Plasmid DNA) |
| Thermo Scientific | F-530L | Phusion High-Fidelity DNA Polymerase (2 U/µL) |
| NEB | R0560S | PmeI |
| Sigma | 76293 | Polyethylene glycol solution |
| FroggaBio | DFL100 | Presto™ Max Gel/PCR Kit |
| NEB | R3140S | PstI-HF |
| Thermo Scientific | EN0531 | RNAse |
| NEB | R3138S | SalI-HF |
| NEB | R0569S | SapI |
| Sigma | 85555-5G | Spectinomycin Dihydrochloride |
| NEB | M0202L | T4 DNA Ligase (NEB) |
| Epicentre | T5E4111K | T5 exonuclease (Epicentre) |
| NEB | C2566I | T7 Express Competent E. coli (High Efficiency) |
| Qiagen | 201203 | Taq DNA Polymerase |
| NEB | M0219S | TaqI Methyltransferase |
| NEB | R0180S | XmaI |
