# Supplementary figures and images for: An innovative platform for quick and flexible joining of assorted DNA fragments
Source: Sci Rep. 2016 Jan 13;6:19278. doi: 10.1038/srep19278 (PMC4725820; doi:10.1038/srep19278)

Created with SnapGene®

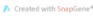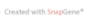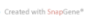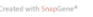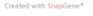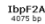

Supplement: Supplementary Information [file srep19278-s2.zip › SnapGene graphical view example of annotated constructs.pdf]
